# Expert consensus-based clinical practice guidelines management of intravascular catheters in the intensive care unit

**DOI:** 10.1186/s13613-020-00713-4

**Published:** 2020-09-07

**Authors:** Jean-François Timsit, Julien Baleine, Louis Bernard, Silvia Calvino-Gunther, Michael Darmon, Jean Dellamonica, Eric Desruennes, Marc Leone, Alain Lepape, Olivier Leroy, Jean-Christophe Lucet, Zied Merchaoui, Olivier Mimoz, Benoit Misset, Jean-Jacques Parienti, Jean-Pierre Quenot, Antoine Roch, Matthieu Schmidt, Michel Slama, Bertrand Souweine, Jean-Ralph Zahar, Walter Zingg, Laetitia Bodet-Contentin, Virginie Maxime

**Affiliations:** 1 APHP/Hopital Bichat-Medical and Infectious Diseases ICU (MI2), 46 rue Henri Huchard, 75018 Paris, France; 2 UMR 1137-IAME Team 5-DeSCID: Decision SCiences in Infectious Diseases, Control and Care Inserm/Université de Paris, Sorbonne Paris Cité, 75018 Paris, France; 3 Department of Neonatal Medicine and Pediatric Intensive Care, Arnaud de Villeneuve University Hospital, 371 Avenue Doyen G Giraud, 34295 Montpellier Cedex 5, France; 4 Infectious Diseases Unit, University Hospital Tours, Nîmes 2 Boulevard, 37000 Tours, France; 5 CHU Grenoble Alpes, Réanimation Médicale Pôle Urgences Médecine Aiguë, 38000 Grenoble, France; 6 Medical ICU, Saint-Louis University Hospital, AP-HP, Paris, France; 7 Centre Hospitalier Universitaire de Nice, Médecine Intensive Réanimation, Archet 1, UR2CA Unité de Recherche Clinique Côte d’Azur, Université Cote d’Azur, Nice, France; 8 Clinique d’anesthésie pédiatrique, Hôpital Jeanne-de-Flandre, avenue Eugène-Avinée, CHU Lille, 59000 Lille, France; 9 Unité accès vasculaire, Centre Oscar Lambret, 3 rue Frédéric Combemale, 59000 Lille, France; 10 Anesthésie Réanimation, Hôpital Nord, 13015 Marseille, France; 11 Service d’Anesthésie et de Réanimation, Hospices Civils de Lyon, Groupement Hospitalier Sud, Lyon, France; 12 UMR CNRS 5308, Inserm U1111, Laboratoire des Pathogènes Émergents, Centre International de Recherche en Infectiologie, Lyon, France; 13 Medical ICU, Chatilliez Hospital, Tourcoing, France; 14 U934/UMR3215, Institut Curie, PSL Research University, 75005 Paris, France; 15 AP-HP, Infection Control Unit, Bichat-Claude Bernard University Hospital, 46 rue Henri Huchard, 75877 Paris Cedex, France; 16 INSERM IAME, U1137, Team DesCID, University of Paris, Paris, France; 17 Pediatric Intensive Care, Paris South University Hospitals AP-HP, Le Kremlin Bicêtre, France; 18 Services des Urgences Adultes and SAMU 86, Centre Hospitalier Universitaire de Poitiers, 86021 Poitiers, France; 19 Université de Poitiers, Poitiers, France; 20 Inserm U1070, Poitiers, France; 21 Department of Intensive Care, Sart-Tilman University Hospital, and University of Liège, Liège, Belgium; 22 Department of Biostatistics and Clinical Research and Department of Infectious Diseases, Caen University Hospital, 14000 Caen, France; 23 EA2656 Groupe de Recherche sur l’Adaptation Microbienne (GRAM 2.0) UNICAEN, CHU Caen Medical School Université Caen Normandie, Caen, France; 24 Department of Intensive Care, François Mitterrand University Hospital, Dijon, France; 25 Lipness Team, INSERM Research Center LNC-UMR1231 and LabExLipSTIC, University of Burgundy, Dijon, France; 26 INSERM CIC 1432, Clinical Epidemiology, University of Burgundy, Dijon, France; 27 Assistance Publique - Hôpitaux de Marseille, Hôpital Nord, Service des Urgences, 13015 Marseille, France; 28 Centre d’Etudes et de Recherches sur les Services de Santé et qualité de vie EA 3279, Faculté de médecine, Aix-Marseille Université, 13005 Marseille, France; 29 Assistance Publique-Hôpitaux de Paris (APHP), Pitié-Salpêtrière Hospital, Medical Intensive Care Unit, 75651 Paris, France; 30 INSERM, UMRS_1166-ICAN, Institute of Cardiometabolism and Nutrition, Pitié-Salpêtrière Hospital, Medical Intensive Care Unit, Sorbonne Universités, 75651 Paris Cedex 13, France; 31 Medical Intensive Care Unit, CHU Sud Amiens, Amiens, France; 32 Medical ICU, Gabriel-Montpied University Hospital, Clermont-Ferrand, France; 33 IAME, UMR 1137, Université Paris 13, Sorbonne Paris Cité, Paris, France; 34 Service de Microbiologie Clinique et Unité de Contrôle et de Prévention Du Risque Infectieux, Groupe Hospitalier Paris Seine Saint-Denis, AP-HP, 125 Rue de Stalingrad, 93000 Bobigny, France; 35 Infection Control Programme and WHO Collaborating Centre on Patient Safety, University of Geneva Hospitals and Faculty of Medicine, Geneva, Switzerland; 36 Medical Intensive Care Unit, INSERM CIC 1415, CRICS-TriGGERSep Network, CHRU de Tours and Université de Tours, Tours, France; 37 Surgical and Medical Intensive Care Unit Hôpital, Raymond Poincaré, 9230 Garches, France

**Keywords:** Catheter, Critically ill, Sepsis, Infection, Bacteremia, Prevention

## Abstract

The French Society of Intensive Care Medicine (SRLF), jointly with the French-Speaking Group of Paediatric Emergency Rooms and Intensive Care Units (GFRUP) and the French-Speaking Association of Paediatric Surgical Intensivists (ADARPEF), worked out guidelines for the management of central venous catheters (CVC), arterial catheters and dialysis catheters in intensive care unit. *For adult patients*: Using GRADE methodology, 36 recommendations for an improved catheter management were produced by the 22 experts. Recommendations regarding catheter-related infections’ prevention included the preferential use of subclavian central vein (GRADE 1), a one-step skin disinfection(GRADE 1) using 2% chlorhexidine (CHG)-alcohol (GRADE 1), and the implementation of a quality of care improvement program. Antiseptic- or antibiotic-impregnated CVC should likely not be used (GRADE 2, for children and adults). Catheter dressings should likely not be changed before the 7th day, except when the dressing gets detached, soiled or impregnated with blood (GRADE 2− adults). CHG dressings should likely be used (GRADE 2+). For adults and children, ultrasound guidance should be used to reduce mechanical complications in case of internal jugular access (GRADE 1), subclavian access (Grade 2) and femoral venous, arterial radial and femoral access (Expert opinion). For children, an ultrasound-guided supraclavicular approach of the brachiocephalic vein was recommended to reduce the number of attempts for cannulation and mechanical complications. Based on scarce publications on diagnostic and therapeutic strategies and on their experience (expert opinion), the panel proposed definitions, and therapeutic strategies.

## Background

Central venous catheters (CVC), arterial catheters and dialysis catheters are inserted in 3 out of 4 critically ill patients’ intensive care unit (ICU). Complications included local insertion site complications, infections and thrombosis [1, 2]. These adverse events are responsible for heavy morbidity and mortality and additional costs, although they can be avoided in the great majority of cases. Healthcare improvement programs and quality improvement strategies have been shown to be effective to prevent complications related to intravascular catheters [3], especially when there the local compliance with the measures.

## Purpose of the guidelines

The purpose of these catheters’ practice guidelines is to address the main issues involved in the management of the vascular access devices used in the ICU, based on the available data on the prevention, diagnosis and management of catheter-related complications.

## Methods

These recommendations are the result of the work conducted by a French Society of Intensive Care Medicine (SRLF) expert committee according to a predefined calendar. The Steering Committee, jointly with the coordinator, initially defined the questions to be addressed, and specific experts addressed each question. After the first expert committee meeting, questions were developed according to a *Patient Intervention Comparison Outcome* (PICO) format. Then, review of the literature and development of recommendations were conducted according to the GRADE methodology (*Grade of Recommendation Assessment, Development and Evaluation*). A level of evidence was defined for each article, according to the study design and the quality of the methodology. A global level of evidence was then determined for each endpoint by taking into account the levels of evidence of each study, the consistency of the results between the various studies, the direct or indirect nature of the evidence, and the cost analysis. A “high” global level of evidence enabled to issue a “strong” recommendation (an intervention must or must not be used… GRADE 1+ or 1−). When the global level of evidence was moderate, low or very low, the recommendation was accordingly lighter (an intervention should probably or probably not be used… GRADE 2+ or 2−). When no or insufficient findings were published, the related recommendation was based on the experts’ opinion (the experts suggest…) (Table 1). Proposed recommendations were discussed one by one. The purpose of the present document is to present the expert opinions and to highlight the items that raised agreement, disagreement or indecision. The collective score was established according to a Delphi methodology. Proposed recommendations were individually scored and were submitted to each expert through a standardized computer form. Experts scored the various items using a discrete 1–9 numerical scale, 1 considered to reflect “full disagreement” and 9, “full agreement”. Three score zones were defined: 1 to 3 reflected a disagreement with the recommendation, 4 to 6 indecision, and 7 to 9, an agreement with the recommendation. A recommendation was validated when at least 50% of experts expressed an opinion (agreement or disagreement) and less than 20% of experts were in disagreement. A strong recommendation could be set out if at least 70% of experts agreed with the recommendation. In the absence of agreement, the recommendations were revised and reviewed again for scoring, up to reaching a consensus.

**Table 1:**
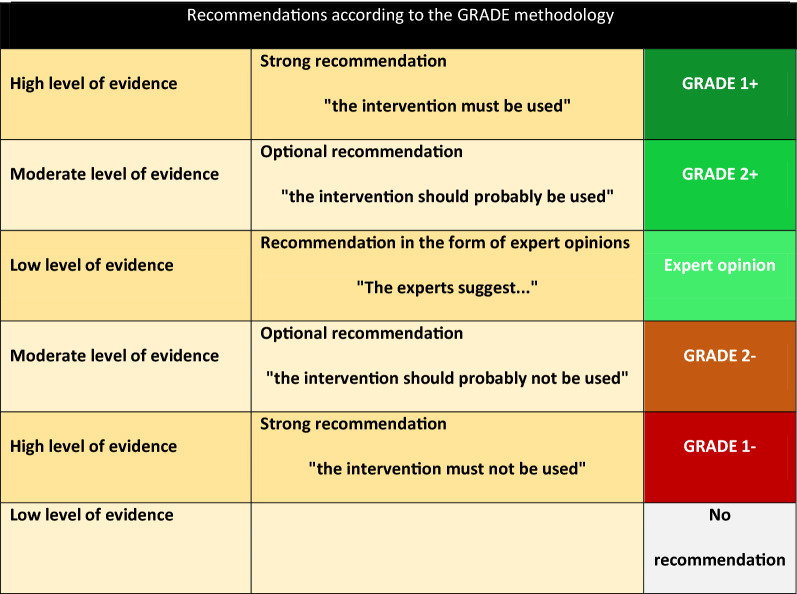
Recommendations according to the GRADE methodology

## Scope of recommendations

Three fields were defined: prevention, surveillance, and treatment. A literature search was performed using the MEDLINE database via PubMed and the Cochrane database for the period from 1980 to 2018. Articles written in French or English were included in the analysis. The literature review focused on recent data according to an order of assessment ranging from meta-analyses and randomised trials to observational studies. Sample sizes and the relevance of the research were considered for each study.

## Summary of the results

For adult patients: Analysis of the publications by the experts and application of the GRADE methodology allowed to issue 36 recommendations. Five of these 36 formal recommendations had a high level of evidence (GRADE 1+/−) and 7 had a low level of evidence (GRADE 2+/−) (see Additional file 1 for detailed scorings). The GRADE methodology could not be applied for 23 recommendations, resulting in expert opinions. After three scoring rounds, a consensus was reached for 35 of the 36 recommendations.

For children: Analysis of the publications by the experts and application of the GRADE methodology allowed to issue 9 recommendations. One of these 9 formal recommendations had a high level of evidence (GRADE 1+/−) and 3 had a low level of evidence (GRADE 2+/−) (see Additional file 1 for detailed scorings). The GRADE methodology could not be applied for 4 recommendations, resulting in expert opinions. After three scoring rounds, a consensus was reached for 8 of the 9 recommendations.

### Definitions

#### Catheter colonization

Catheter colonization is defined by a semi-quantitative culture ≥ 15 CFU, according to Maki, or a quantitative culture ≥ 10^3^ CFU/mL, according to Brun-Buisson.

#### Suspicion of catheter-related infection (CRI): (EXPERT OPINION)

In the presence of a central venous catheter (CVC) or arterial catheter (AC), a CRI is suspected based on the presence of at least one of the following criteria:

1. Onset or worsening of systemic signs of acute inflammation (fever or organ dysfunction) following catheter placement, with no other site of infection, and with likely no other non-infectious cause (drug-related adverse reaction, venous thromboembolism, etc.).
2. Onset of local signs around the catheter (cellulitis, tunnel infection, purulent discharge or abscess at the insertion site).
3. Positive blood culture with no confirmed other source than the presence of CVC or AC line.*

NB: If the blood culture is drawn from an AC, a negative catheter culture is required to confirm the diagnosis.

NB*: In the case of bacteraemia due to skin commensal bacteria (such as coagulase-negative staphylococci, *Corynebacterium* spp. and *Cutibacterium* spp.), two blood cultures with identical antibiotic susceptibility test results are required to confirm the diagnosis.

The definition used for suspicion of catheter-related infection was not specified in studies and recommendations on the management of catheter-related infections [2, 4]. To propose standardised therapeutic decision trees and recommendations, we clarified the criteria that should lead to a catheter removal and culture.

#### Risk factors for complications or signs of infection severity

The risk factors for complications or signs of infection severity that should probably be investigated in the presence of suspected CRI for deciding appropriate therapy (EXPERT OPINION):

- Haemodynamic instability: systolic blood pressure (SBP) < 90 mmHg (or 40 mmHg decrease in SBP compared to baseline) or mean blood pressure < 65 mmHg in the absence of another cause of hypotension, or need for vasopressors or inotropic agents to maintain adequate blood pressure during the previous 12 h.
- Neutropenia (< 500/mm^3^)
- Organ transplantation and other forms of immunosuppression
- Intravascular devices (pacemaker, prosthetic heart valve, vascular prosthesis, etc.)
- Suppuration or frank induration/erythema (> 0.5 cm in diameter) at the involved vascular access site.

This definition of the infection severity is based on the results of a study published in 2004 [5]. Patients with suspected CVC infection were randomised to an “immediate catheter removal” group versus a “watchful waiting” group. Patients were excluded if they had the signs of severity reported above. Of 144 screened patients with suspected CRI, 80 had at least one exclusion criterion, among which 25% were diagnosed with catheter-related bloodstream infection. A CRI was diagnosed in 2/32 patients randomised in the “immediate removal” group, and 3/32 patients randomised in the “watchful waiting” group, i.e., an overall CRI rate of 7.8%. In the watchful waiting group, 37% of central venous catheters were removed between inclusion and day 10 due to persistent sepsis, haemodynamic instability, catheter dysfunction, or protocol violation. The authors concluded that the results of their study supported the Infectious Disease Society of America (IDSA) recommendation for the diagnosis and management of catheter-related infections stating that “non-tunnelled central venous catheters should not be routinely removed in patients with moderately severe disease” [6].

#### Non-bacteraemia catheter-related infection (EXPERT OPINION)

In the absence of bacteraemia, the diagnosis of CRI is based on a combination of

- catheter culture ≥ 10^3^ CFU/mL (quantitative method) or ≥ 15 CFU (semi-quantitative method)
- and (a) signs of local infection (purulent discharge from the catheter insertion site or tunnel infection); *and/or* (b) systemic signs, with complete or partial resolution of systemic signs of infection within 48 h after catheter removal.

#### Uncomplicated catheter-related infection

The “uncomplicated” nature of a catheter-related infection is defined by a favourable clinical (apyrexia) and bacteriological course (negative blood cultures) after 72 h of treatment, in the absence of metastatic infection site, endocarditis or suppurative thrombophlebitis.

NB: The term “uncomplicated CRI” excludes any infection related to a permanent intravascular device at increased risk of complications, such as pacemaker, prosthetic valve, etc.

#### Catheter-related bacteraemia or fungaemia

Catheter-related bacteraemia or fungaemia is defined as

1. The occurrence of either bacteraemia or fungaemia during the 48-h period surrounding catheter removal (or a suspected diagnosis of CRI when the catheter is not removed immediately)
2. And
  either a positive culture with the same microorganism on one of the following samples: insertion site culture, or catheter culture ≥ 10^3^ CFU/mL
  or positive central and peripheral blood cultures with the same microorganism, with a central/peripheral quantitative blood culture ratio > 5, or a central/peripheral positive blood culture time-lag > 2 h, with central blood cultures being positive earlier than the peripheral ones.

#### Persistent catheter-related bacteraemia or fungaemia

Persistent catheter-related bacteraemia or fungaemia is defined as the persistence of positive blood cultures after 3 days (72 h) of a well-conducted antibiotic or antifungal therapy.

### First field: prevention

**R1.1—To decrease the risk of central venous catheter-related infection, the subclavian vein should be used rather than the jugular or femoral vein, in the absence of contraindication. This recommendation does not apply to venous catheters used for renal replacement therapy.**

**GRADE 1+ STRONG CONSENSUS**

Many non-randomised studies compared the risk of infection according to the site of catheter insertion. Four meta-analyses were conducted on these studies, resulting in various conclusions [7–10].

The randomised trial conducted by Merrer et al. [11] concluded to the superiority of the subclavian site compared to the femoral site (Hazard Ratio (HR: 4.83), 95% confidence interval (CI) [1.96–11.93]). However, this old study did not use the currently available preventive measures. A recent multicentre, randomised trial [12] demonstrated again the superiority of the subclavian site compared to the femoral site (CR-BSI: HR: 3.4, 95% CI [1.0–11.1]); the intention-to-treat analysis showed a trend towards superiority of the subclavian site compared to the internal jugular site (HR: 2.3, 95% CI [0.8–6.2]), while the per protocol analysis showed a significant superiority of the subclavian site over the internal jugular site (HR: 3.8, 95% CI [1.0–14.1]). Finally, a meta-analysis [7] that compared the subclavian site to the internal jugular site demonstrated a higher risk of infection with the internal jugular site (RR: 1.8 [1.0–3.4]), but with marked heterogeneity and a large proportion of non-randomised studies. Of note, the inferior (or posterior) internal jugular site appeared to be associated with a lower risk of infection [13] compared to the superior (or central) internal jugular site, but with a low level of evidence (single-centre non-randomised study) [8].

NB: The generally accepted contraindications to subclavian vein cannulation are severe primary or secondary clotting disorders (platelet count < 50 × 10^9^/L or PT < 30% [INR > 2]), a PaO_2_/FiO_2_ ratio < 200 mmHg, or any situation in which the respiratory status is precarious or unstable, associated with a high risk of barotrauma.

**R1.2—The internal jugular vein is probably not preferable to the femoral vein for central venous catheter insertion to decrease the infection rate.**

**GRADE 2− STRONG CONSENSUS**

Four meta-analyses based on a total of 25,047 catheters did not yield any significant differences in terms of infection rates between internal jugular and femoral sites [8, 9, 14, 15]. Two of these meta-analyses suggested a lower infection rate in favour of the internal jugular site (RR 0.55; 95% CI [0.34–0.89]; *I*^2^ = 61% and RR 1.90; 95% CI 1.21–2.97; *I*^2^ = 35%), but this difference was no longer observed when the analysis was confined to randomised trials [8, 9]. Observational studies that included critically ill patients tend to show equivalence of the two insertion sites, or even a slight advantage in favour of the internal jugular site [16–20]. Randomised trials did not demonstrate the superiority of the internal jugular site compared to the femoral site [12, 21, 22], apart from a difference in terms of catheter tip colonization rates in only one study, in favour of the internal jugular site (RR 1.6; 95% CI 1.2–2.0) [12]. The internal jugular insertion site has been suggested to be superior in patients with a high body mass index (BMI) (BMI > 28.4 kg/m^2^) [14, 15, 21]. The effect of time on infectious complications according to the insertion site remains debated. Timsit et al. suggested a benefit of the internal jugular site when the catheter was left in place for more than 5 days [15], while no time effect was investigated in the other studies [22].

**R1.3—2% chlorhexidine-alcohol rather than povidone-iodine/alcohol should be used for skin disinfection before intravascular catheter insertion to decrease the infection rate.**

**GRADE 1+ STRONG CONSENSUS**

Although the superiority of aqueous chlorhexidine solution compared to aqueous povidone-iodine solution has been demonstrated for many years [23], studies in favour of the use of chlorhexidine-alcohol were limited by the lack of appropriate comparator, usually aqueous povidone-iodine [24, 25].

Two recent studies confirmed the superiority of chlorhexidine-alcohol compared to povidone-iodine/alcohol. A multicentre randomised controlled single-blind trial that compared the two products for all types of vascular access showed a 79% reduction of catheter-related bacteraemia rates (HR, 0.21; 95% CI [0.07–0.59]), catheter-related infection and catheter colonisation, as well as skin colonisation at catheter removal [26]. However, severe catheter site cutaneous adverse effects were significantly more frequent with chlorhexidine than with povidone-iodine.

The other study used data from a multicentre study that compared the infectious risk according to the catheter insertion site [27]. Using a propensity score method, the risk of catheter infection (but not the risk of CVC-related bacteraemia) was reduced by 50% in the 2% chlorhexidine-alcohol group compared to the povidone-iodine/alcohol group. However, this quasi-experimental ancillary study using a propensity score was associated with a risk of bias.

**R1.4—A one-step disinfection should be performed before intravascular catheter insertion.**

**GRADE 1+ STRONG CONSENSUS**

Only one high-powered European randomised controlled trial [26] evaluated the efficacy of skin disinfection modalities, i.e., four-step disinfection with scrubbing of the skin (detergent, rinsing, drying and disinfection) versus one-step disinfection (only one application of disinfectant on macroscopically clean skin), with two different antiseptics, povidone-iodine/alcohol versus chlorhexidine-alcohol. Cleaning the skin with detergent is one of the French recommendations devised to reduce the number of microorganisms on the skin and improve the efficacy of disinfection [28, 29]. Regardless of the antiseptic used, this study showed that this cleansing step in no way decreased the infection, bacteraemia or catheter colonisation rates.

A systematic review was published by the Cochrane group [30] on the various types of antisepsis devised to reduce catheter-related infections, including 13 randomised controlled trials designed to evaluate all types of skin antiseptics, used alone or in combination, compared to one or several other skin antiseptics, placebo, or complete absence of antisepsis, in patients with a central venous catheter in place. The authors of this analysis concluded that the potential benefit of skin disinfection on the infectious risk compared to absence of disinfection was not demonstrated. However, the evidence obtained was of very poor quality (single-centre low-powered studies with numerous biases). No study analysed the modalities of antisepsis. In the CLEAN study an applicator was only used for the CHG disinfection. Therefore, the impact of applicator use on the superiority of chlorhexidine-alcohol remains to be evaluated [31].

**R1.5—Antimicrobial (antiseptic or antibiotic)-impregnated central venous catheters should probably not be used to decrease the incidence of bacteraemia.**

**GRADE 2− STRONG CONSENSUS**

One of the measures proposed to reduce the rate of CVC-related infections is the use of antimicrobial-impregnated catheters, using either antiseptics (chlorhexidine, silver sulphadiazine) or antibiotics (minocycline–rifampin combination). A meta-analysis of randomised controlled trials comparing antimicrobial-impregnated CVC versus standard CVC [32] showed (1) a decreased risk of CVC-related bacteraemia in the antimicrobial-impregnated CVC group using a combination of chlorhexidine/silver sulfadiazine (RR 0.73; 95% CI [0.57–0.94]) or minocycline–rifampin (RR 0.26; 95% CI [0.13–0.49]); (2) no reduction of the risk of CVC-related bacteraemia expressed per 1000 catheter-days regardless of the antimicrobial used; and (3) no reduction of the risk of local infection regardless of the antimicrobial used.

**R1.6—Published data in adults are insufficient to formulate a recommendation concerning the use of a heparin-bonded catheter to decrease the thrombosis rate.**

**NO RECOMMENDATION**

The review of the literature failed to identify any studies concerning this issue in adult critically ill patients. However, Shah et al. published in 2014 [33], a Cochrane review of the literature designed to determine the effects of heparin-bonded CVC on the risk of catheter-related thrombosis, occlusion, bloodstream infection and side effects in children. CVC are just as useful in children as in adults (for parenteral nutrition, drug infusion, haemodynamic monitoring, etc.) and heparin bonding could decrease the thrombogenic risk of the device and therefore platelet aggregation, but could also reduce adhesion of bacteria such as staphylococci. By pooling data from the only two randomised controlled trials comparing heparin-bonded versus standard CVC, Shah et al. showed a relative risk of thrombosis (clinical or on Doppler ultrasound) of 0.31 (95% CI 0.01–7.68) and a risk of occlusion of 0.22 (95% CI 0.07–0.72) (only one study analysed).

**R1.7—Chlorhexidine-impregnated dressings should probably be used to decrease arterial or central venous catheter-related infection rates.**

**GRADE 2+ STRONG CONSENSUS**

Studies specifically conducted in critically ill patients [34–37] failed to demonstrate any reduction of catheter colonisation or catheter-related bacteraemia rates with the use of chlorhexidine-impregnated sponges placed over the central venous catheter insertion site compared to a standard dressing [34, 35]. Of note, these studies included a small number of patients with rather high catheter colonisation rates. A multicentre randomised controlled trial including 1636 adults [36] showed that the use of chlorhexidine-impregnated sponges placed over the arterial or central venous catheter insertion site decreased the catheter-related bacteraemia rate from 1.3 to 0.5 (OR 0.4; 95% CI [0.19–0.87]) infections per 1000 catheter-days and the catheter colonization rate from 10.9 to 4.3 (OR 0.41; 95% CI [0.31–0.56]) per 1000 catheter-days compared to a standard dressing. Another trial conducted by the same team on 1879 patients [37] showed that the use of a chlorhexidine gel-impregnated dressing placed over the arterial or central venous catheter insertion site decreased the catheter-related bacteraemia rate from 1.3 to 0.5 (OR 0.4; 95% CI [0.19–0.87]) infection per 1000 catheter-days, and the catheter colonisation rate from 10.9 to 4.3 (OR 0.41; 95% CI [0.31–0.56]) per 1000 catheter-days, compared to a standard dressing. These studies did not demonstrate any correlation between the infection rate per centre in the control group and the efficacy of the dressing. No significance difference in terms of efficacy was observed between arterial and venous catheters. The limitations of these studies included the absence of double-blind design, skin antisepsis with povidone-iodine/alcohol at the time of catheter insertion and when changing dressings (first study) and a manufacturer’s financial participation, especially in the second study [37]. Of the various meta-analyses [39–41], the most recent [41], that combined four studies including three studies performed in intensive care units, showed that the use of chlorhexidine-impregnated sponges or dressings decreased the catheter-related bacteraemia rate (OR 0.51; 95% CI [0.33–0.78]) and the catheter colonization rate (OR 0.58; 95% CI [0.47–0.73). However, these studies showed significantly higher rates of dermatitis with chlorhexidine dressings (1.5% and 2.3%, respectively) than with standard dressings (1% in the two studies [36, 37], the need to stop impregnated dressings in 1.1% of patients [37], and cases of serious dermatitis in patients with pre-existing skin diseases [42] and in children [35, 43]. Several studies [44–48] have suggested that the use of chlorhexidine-impregnated dressings had a favourable cost–benefit ratio, with a cost reduction of USD 100 to 964 per catheter inserted.

**R1.8—Catheter dressings should probably not be changed before the 7th day, except when the dressing has become detached, contaminated or impregnated with blood.**

**GRADE 2− STRONG CONSENSUS**

Four randomised trials [36, 49–51] conducted in Europe between 1995 and 2009 evaluated less-frequent dressing changes (once vs. twice weekly [50, 51], 15 vs. 4 days [49] and 7 vs. 3 days [36]). Three small studies [49–51] (including one study in children [49]) included patients with a cancer or haematological malignancy requiring a central venous catheter. Only one study [36] was conducted in the intensive care setting and included 1636 adults with a central venous and/or arterial catheter. These studies showed that the risk of local or systemic catheter-related infection, skin lesions or mortality was not increased by a longer dressing change interval. Nevertheless, detachment or the presence of soiling or bleeding under the dressing required earlier than planned dressing change in the “long dressing change interval” arms. In the only study conducted in critically ill patients [36], the median time to dressing changes per catheter was 3 [2–5] days in the “7-day” arm versus 4 [3–6] days in the “3-day” arm (*p* < 0.001).

**R1.9—Internal jugular venous catheters should be inserted with ultrasound guidance to reduce the mechanical complication rate.**

**GRADE 1+ STRONG CONSENSUS**

Internal jugular vein catheterisation using anatomical landmarks is associated with complications, essentially arterial puncture and haematoma, in about 20% of cases. The overall success rate is about 86%, and the first attempt success rate using anatomical landmarks is 55% [52–58]. Ultrasound guidance decreases the internal jugular vein catheterisation time, and increases both the overall success rate and the first attempt success rate. Ultrasound guidance also decreases the complication rate, essentially arterial puncture and haematoma, which can be severe in patients with clotting disorders. Several meta-analyses [59, 60] have been published and have confirmed these conclusions on larger populations. These results are steadily yielded regardless of the patient subpopulations studied (children, adults) or the operators (experienced or inexperienced), although these subgroups are small, with a low level of evidence. Only a few studies have analysed distant infectious complications, and ultrasound guidance does not appear to be associated with any particular risk, although the small sample sizes hampered to draw any definitive conclusions [53]. Most studies have used axial scan (with an out-of-plane approach) rather than longitudinal scans of the internal jugular vein (with an in-plane approach). No recommendation can be proposed on this subject based on the contradictory data.

**R1.10—Subclavian venous catheters should probably be inserted with ultrasound guidance to decrease the mechanical complication rate.**

**GRADE 2+ STRONG CONSENSUS**

A meta-analysis published by the Cochrane review in 2014 analysed data derived from nine studies and more than 2000 patients [59]. This meta-analysis showed a significant reduction of the number of accidental arterial punctures (RR 0.21; 95% CI [0.06–0.82]) and the number of haematomas during the procedure (RR 0.26; 95% CI [0.09–0.76]). In contrast, this meta-analysis did not find any difference in terms of the total number of complications, the number of catheterisation attempts required, the first attempt success rate or the duration of the procedure [59]. However, the heterogeneity and lack of precision of the results of the studies included in this meta-analysis should be highlighted. Another meta-analysis published in 2015 analysed data derived from 10 studies and 2168 patients [61]. Although these studies presented the same limitations as those of the Cochrane review, the authors nevertheless observed a significant reduction of the total number of complications with the use of ultrasound guidance (OR 0.53; 95% CI [0.41–0.69]). Based on the study populations, their sample sizes, and the presence of several limitations (see details in Additional file 1), the benefit of systematically performing subclavian vein catheterisation with ultrasound guidance could not obtain a Grade 1 [56, 62, 63]. Finally, the ultrasound-guided subclavian vein catheterisation technique also remains a subject of debate: in-plane or out-of-plane catheterisation, systematic use of Doppler ultrasound during the procedure [62, 64, 65]. Due to the small sample sizes, the limited number of published studies and the limitations described above, the superiority of one strategy over another cannot be clearly demonstrated.

**R1.11—The experts suggest that femoral vein catheterisation should be performed with ultrasound guidance to decrease the mechanical complication rate.**

**EXPERT OPINION**

The most recent meta-analysis on femoral venous catheters was based on four randomised prospective studies, including one study conducted in the perioperative setting, but no studies in the intensive care setting [59]. The studies included in this meta-analysis, although presenting a low level of quality, showed a reduction of failure rates, complication rates, catheterisation time and number of attempts [62, 66, 67].

**R1.12—The experts suggest that femoral and radial artery catheterisation should be performed with ultrasound guidance to decrease the mechanical complication rate.**

**EXPERT OPINION**

Few studies have tried to evaluate the value of ultrasound guidance for radial or femoral artery catheterisation in adults. The theoretical advantages of ultrasound guidance are a lower complication rate during the procedure (puncture site haematoma), a higher success rate and a shorter cannulation time. Femoral artery catheterisation has essentially been evaluated only in interventional radiology rooms [68]. A meta-analysis published in 2015, based on four studies including 1422 patients, suggested a lower complication rate (accidental venous puncture or puncture site haematoma) with ultrasound guidance (RR 0.51; 95% CI 0.28–0.91) [68].

Several studies have evaluated ultrasound guidance for radial artery catheterisation. A meta-analysis published in 2016 included data derived from five randomised studies [69] conducted in adults, usually in the operating room [70–73]. The authors of this meta-analysis only observed a higher first attempt success rate (RR 1.4; 95% CI 1.28–1.64) and therefore a lower number of attempts before successfully cannulating the radial artery. The magnitude of the benefit of systematically using ultrasound guidance appeared to depend on the operator’s experience: ultrasound guidance was less beneficial for the most experienced operators [72]. The various studies reported contradictory results regarding the decrease of the procedure duration or complication rate when using ultrasound guidance.

### Second field: surveillance

**R2.1—The experts suggest that the incidence of catheter-related infections is decreased when the intensive care unit is part of a surveillance network.**

**EXPERT OPINION**

Surveillance networks have a positive impact on several aspects. Common definitions allow within- and between-unit comparability. The main definitions are those of the American CDC and the European ECDC [74, 75], used in France with variable consistency [76, 77]. Apart from classical studies concerning the relationship between surveillance and the course of ICU-acquired infection rates [78], two studies showed that network surveillance [79, 80] was associated with a decrease in infection rates in quasi-experimental studies. Several examples of the setting up of prevention programmes have been published in the literature in the USA [81], and also in Europe (Spanish Bacteraemia Zero programme [82]). At the request of the European Commission, the ECDC has set up measurement of structure and process indicators [83], which were also used in France [84]. Study of risk factors for infection benefits from network surveillance, which enables to evaluate and validate putative risk factors on a large number of patients, to guide prevention measures [85]. It is still very difficult to obtain relevant aggregated bacterial ecology data that reflect French ecology, and aggregated antibiotic consumption data [86]. Finally, network surveillance might also be useful for less objective indicators, such as membership of a network, and the possibility of comparisons to identify outliers [87]. The panel recognises that the information bias due to increased awareness and peer pressure may have amplified the benefit of surveillance network participation.

**R2.2—A quality of care improvement programme should be set up in intensive care units to reduce catheter-related bloodstream infection rates.**

**GRADE 1+ STRONG CONSENSUS**

The use of check-lists for catheterisation and catheter care and the use of “all inclusive” trolleys and kits facilitate compliance with the quality improvement programme by standardizing central venous catheterisation and catheter care practices. A total of 48 studies (published in or after 2006) were reviewed to address this issue [3, 70, 81, 82, 88–131]. Three good-quality randomised controlled trials showed that applying a “best practice” programme elicited a significant reduction of CVC-related bacteraemia rates [3, 109, 115]. A reduction of infection rates as a result of improved compliance with clinical practice guidelines was clearly shown in two studies [3, 122]. The majority of published studies used a quasi-experimental methodology without a control group, and were therefore considered to present a low level of quality according to the GRADE methodology [92]. Overall, these studies showed a reduction of catheter-related bloodstream infection by about 55% in adult critically ill patients, and about 42% in paediatric critically ill patients [132]. A multimodal strategy [133] and simulation centre-based training were shown to be effective in the improvement of clinical practices (Table 2) [93, 109].

**Table 2:** Strategies proposed by experts to allow a reduction of catheter-related infection

For the catheter insertion	During catheter care
Hand hygiene	Hand hygiene
Maximum hygiene and asepsis measures (cap, mask, sterile gown, sterile gloves, large sterile fields)	Regular inspection of dressings Change semipermeable transparent dressings every 7 days (except in the case of detachment, soiling or bleeding)
2% Chlorhexidine-alcohol for skin antisepsis	Change of tubing after 96 h (or after 24 h in the case of lipids or blood products).
	Disinfect valves before accessing or manipulating open systems on a sterile compress or an alcohol compress
	Remove the catheter as soon as it is no longer necessary

**R2.3—The experts suggest culture of central venous or arterial catheters only when there is a suspicion of catheter-related infection.**

**EXPERT OPINION**

The value of systematic culture of central venous or arterial catheters in the intensive care unit was not assessed through any randomised trials, neither for CRI prevention (i.e., reduction of the incidence of CRI), nor treatment (i.e., identification of colonised catheters that may require secondary treatment).

A systematic review including 29 studies published between 1990 and 2002 suggested a correlation between CVC colonisation (predominantly diagnosed using the Maki method) and catheter-related bloodstream infection [134].

Four (2 retrospective and 2 prospective) observational cohort studies were identified, that included predominantly critically ill patients whom CVC or AC were systematically cultured at removal [135–138]. Various culture methods were used (Brun–Buisson and Maki). The indications for catheter withdrawal were systematic in two studies, and based on multiple criteria, including suspicion of catheter-related infection, in the other two studies. The catheter colonisation rate fluctuated between 6 and 15%. The catheter-related infection rate ranged between 1.3 and 4%, and the species most frequently responsible for catheter-related bloodstream infection occurring after catheter removal were *Staphylococcus aureus*, enterobacteria and *Candida* spp.

Two international guidelines recommend catheter culture if and only if catheter-related bloodstream infection is suspected. However, almost two patients out of three are febrile. Signs of infection are commonly present on the day of catheter removal in critically ill patients [139].

### Third field: diagnosis and treatment of catheter-related infections

#### Diagnosis of catheter-related infection

**R3.1—Peripheral blood cultures should probably be performed as soon as** ***S. aureus*** **catheter colonisation is detected.**

**GRADE 2+ STRONG CONSENSUS**

Catheter-related infection with blood cultures positive for *Staphylococcus aureus* is a serious complication, associated with a high potential for metastatic infection through haematogenous spread [140]. *S. aureus* catheter colonisation is frequently associated with concomitant *S. aureus* bacteraemia [141], especially when the catheter is removed for suspicion of infection [142]. The presence of *S. aureus* catheter colonisation is associated with a high incidence of bacteraemia or haematogenous metastatic infection, commonly occurring within 4 days after removal of the colonised catheter [143, 144].

In patients with suspected CRI and with catheter culture positive for *S. aureus,* only 20% had a positive blood culture within the 48 h following catheter removal. The 6-month risk of developing *S. aureus* bacteraemia or metastatic infection was higher when specific antimicrobial therapy was not immediately administered [142].

**R3.2—In the presence of central venous catheter-related infection with persistent bacteraemia or fungaemia, the experts suggest to assess local and systemic complications using at the very least vascular ultrasound examinations and/or CT scan for thrombosis and/or embolism diagnosis.**

**EXPERT OPINION**

Persistent bacteraemia (or fungaemia) is defined as the persistence of positive blood cultures after 3 days of a well-conducted antibiotic therapy. CVC infections can elicit local and/or systemic complications. Local complications include suppuration at the catheter insertion site, tunnel infection and suppurative thrombophlebitis. Systemic complications are related to bacteraemia that may lead to endocarditis and/or septic emboli (especially in the retina). Pulmonary circulation is more commonly involved, especially in the context of *S. aureus* and *Candida* spp. infections [145–147]. This issue has not yet been specifically investigated in critically ill patients. Randomised controlled trials on the clinical impact of procedures identifying local and systemic complications due to CVC are lacking.

**R3.3a—The experts suggest that transoesophageal echocardiography should be performed as soon as possible in all patients with persistent fungaemia or bacteraemia with** ***Staphylococcus aureus*** **or** ***Enterococcus*** **spp.**

**EXPERT OPINION**

**R3.3b—In the presence of central venous catheter-related infection with persistent bacteraemia, regardless of the involved microorganism, the experts suggest that transoesophageal echocardiography should be performed as soon as possible in patients at high risk of endocarditis: haemodialysis, embolism, intravenous drug use, implantable port, intracardiac electronic device, prosthetic valve, valvular and structural heart disease.**

**EXPERT OPINION**

The risk of infective endocarditis depends on the aetiological agent responsible for bacteraemia. Persistent *S. aureus* bacteraemia is associated with a higher recurrence rate and a higher mortality rate during the 12 weeks following an episode of bacteraemia [148]. In a study with repeated blood cultures every 3 days following an episode of *S. aureus* bacteraemia, the rate of complicated infection was 5% when bacteraemia lasted less than 3 days, but increased to 25% in the presence of confirmed bacteraemia persisting more than 10 days [140]. Persistent candidaemia was also associated with a high mortality rate with an adjusted risk of 2.5 (95% CI [1.33–4.72]). The incidence of endocarditis in patients with candidaemia has been less extensively evaluated. In a recent study, trans-thoracic echocardiography (TTE) allowed to diagnose endocarditis in 2.9% of patients and transoesophageal echocardiography (TOE) in 11.5% of patients [149].

The need to systematically exclude the presence of endocarditis in patients with documented *Enterococcus* spp. CRI remains a subject of debate. In a recent study on 1515 patients with *Enterococcus* bacteraemia, 65 (4.29%) patients had documented endocarditis and 16.7% of bacteraemia patients were diagnosed by TTE and 35.5% were diagnosed by TOE.

Insufficient data are available concerning Gram-negative bacilli.

The risk of infective endocarditis also depends on the patient’s predisposing conditions, especially the presence or absence of pre-existing valvular heart disease. Several recent studies [150–155] suggest that, because of the very low risk of infective endocarditis, TOE should only be performed in the presence of the following risk factors: persistent bacteraemia, haemodialysis, community-acquired infection, septic emboli, immunological or embolic phenomena, intravenous drug use, implantable port, intracardiac device, prosthetic valve, history of infective endocarditis, or structural cardiac abnormalities. [patients with any prosthetic valve, including a transcatheter valve, or those in whom any prosthetic material was used for cardiac valve repair; patients with a previous episode of infective endocarditis (IE); patients with cardiomyopathy heart disease (CHD) (any type of cyanotic CHD, any type of CHD treated with a prosthetic material, whether placed surgically or by percutaneous techniques, up to 6 months after the procedure, or lifelong if residual shunt or valvular regurgitation remains)].

**R3.4—The experts suggest that blood cultures should be taken simultaneously from the catheter and by peripheral venous puncture using differential quantitative blood cultures and/or differential time to positivity methods.**

**EXPERT OPINION**

A meta-analysis showed that concomitant quantitative blood cultures allowed the diagnosis of catheter-related bloodstream infection with a sensitivity of 79% and a specificity of 94% [156]. Other studies have been performed since this initial publication [157–159], but they used different limits of significance and variable catheter culture methods and combined different types of catheters.

With the same variations in terms of diagnostic methods and catheters, seven studies [158–164] assessed the validity of a differential time-to-positivity of cultures of at least 2 h in the bottle taken from the catheter compared to that taken from a peripheral vein for the diagnosis of catheter-related bloodstream infection.

The results in terms of sensitivity, specificity, negative predictive value (NPV) and positive predictive value (PPV) were as follows:

**Table:** .

	Sensitivity (%)	Specificity (%)	PPV (%)	NPV (%)
Quantitative blood cultures	71–93	95–99	83–100	95–99
Time-to-positivity	44–96	90–100	61–94	89–99

#### Clinical settings

**R3.5—In a patient with fever and** ***with no signs of severity*, no local signs,** fever not due to **a non-infectious cause, and no other suspected site of infection, the experts suggest that the catheter should be removed**

**EXPERT OPINION**

This recommendation is justified by two findings reported in the literature: (1) more than 20% of central catheters in place on a given day are not justified, even in the intensive care unit [165], and (2) the maintenance of a catheter in the presence of proven infection is always hazardous and associated with mortality excess [166, 167], particularly in the case of multidrug-resistant bacteria.

**R3.6—In a patient with fever and** ***with no signs of severity*, if the catheter cannot be replaced without a major risk, the experts suggest that, rather than immediately removing the catheter, simultaneous blood culture should be obtained by peripheral venous puncture and directly from the suspected catheter using differential quantitative blood cultures and/or differential time to positivity methods.**

**EXPERT OPINION**

This recommendation is based on the results of a study published in 2004 [5]. Patients with suspected central venous catheter-related infection were randomised to an immediate catheter removal group or a watchful waiting group. This study excluded patients with the signs of severity here-above reported. Eighty of the 144 screened patients had at least one exclusion criterion that required the removal of the CVC. A CRI was diagnosed in 25% of these patients. Among the 64 randomised patients, the CVC was immediately removed in 32 patients and a CRI was diagnosed in 2 of them. In the watchful waiting group, 37% of the CVCs were eventually removed between inclusion and Day 10 due to persistent sepsis, haemodynamic instability, catheter dysfunction, or protocol violation, and 3 CRIs were diagnosed. Therefore, 8% of the included patients had a CRI.

NB: signs of severity are defined in the introduction.

**R3.7—In a critically ill patient with suspected CRI and** ***with signs of severity* and no other sites of infection, the experts suggest that the catheter should be removed after taking blood cultures from a peripheral vein and from the catheter.**

**N.B.: When the blood culture is taken from an arterial catheter (AC), the catheter culture must be negative to validate the diagnosis of bacteraemia.**

**EXPERT OPINION**

CRIs are the main cause of sepsis, in about 5% of patients [168, 169]. A systematic review of the literature published in 2018 considered catheter removal to be the instrumental intervention that must always be recommended, especially in the presence of sepsis or shock [2]. This recommendation also applies to neutropenic patients [170]. This recommendation is based on a globally low level of evidence comprising indirect factors, such as the excess mortality of neutropenic patients with septic shock in whom the catheter is not removed [171]. Technological progress, particularly the systematic use of ultrasound for CVC insertion, has facilitated venous cannulation by increasing the success rate by 10 to 80% and reducing the rate of mechanical complications by 50%, especially for subclavian and internal jugular veins [59]. This progress must encourage systematic change of catheter site in the presence of infection. Taking blood cultures before initiating antibiotic therapy in patients with suspected infection is associated with decreased mortality [169].

**R3.8—In critically ill patients** ***with CRI*, the experts suggest that the duration of antibiotic therapy should take into account the microorganism identified, the types of microbiological samples and the possible complications** (Table 3).

**Table 3:** Unexplained fever, catheter removed and positive microbiology (EXPERT OPINION)

Catheter removed in a context of fever and positive microbiology	Antibiotics and duration
*Staphylococcus aureus*, *Candida* spp.	
Negative blood culture	3–5 days
Positive blood culture with no remote complications	14 days
Positive blood culture with remote complications	4 to 6 weeks
*Enterobacteriaceae*, enterococci, coagulase-negative *Staphylococcus*	
Negative blood culture	No antibiotics^a^
Positive blood culture with no distant complications	7 days
Positive blood culture with remote complications	4 to 6 weeks
*Pseudomonas aeruginosa*, *Acinetobacter baumannii*	
Negative blood culture	3–5 days^a^
Positive blood culture with no distant complications	7 days
Positive blood culture with distant complications	4 to 6 weeks

^a^These proposals are based on poor-quality epidemiological data and are only presented as a guide. They must be modulated according to the presence of signs of clinical sepsis, intravascular devices, and underlying immunosuppression

**EXPERT OPINION**

The duration of treatment of catheter-related infections depends on two factors: the microbial species involved and the presence or absence of local or distant complications. No randomised trial is available to define the adequate duration of treatment. By convention, and probably due to the risk of secondary sites of infection and/or endovascular tropism, the experts recommend a prolonged duration of antibiotic therapy (14 days) in the presence of *Staphylococcus aureus* and *Candida* spp. infection, while the duration of treatment is 7 days in other settings.

In the case of secondary sites of infection, the duration of treatment is adapted to the site. Only suppurative thrombophlebitis, defined by the discovery of thrombosis and persistence of bacteraemia after 72 h of effective treatment, requires a minimum duration of treatment of at least 4 weeks, possibly pursued for 6 weeks.

The duration of treatment of suppurative thrombophlebitis is based on expert opinions. A recent retrospective study on patients with *S. aureus* suppurative thrombophlebitis showed that a duration of antibiotic therapy shorter than 28 days and the absence of anticoagulant therapy were associated with rates of higher treatment failure [172].

The duration of treatment of catheter-related infections depends on two factors: the microbial species involved and the presence or absence of local or distant complications. No randomised trial is available to define the adequate duration of treatment. By convention, and probably due to the risk of secondary sites of infection and/or endovascular tropism, the experts recommend a prolonged duration of antibiotic therapy (14 days) in the presence of *S. aureus* and *Candida* spp. infection, while the duration of treatment is 7 days in other settings.

In the case of secondary sites of infection, the duration of treatment is adapted to the site. Only suppurative thrombophlebitis, defined by the discovery of thrombosis and persistence of bacteraemia after 72 h of effective treatment, requires a minimum duration of treatment of at least 4 weeks, possibly pursued for 6 weeks.

The duration of treatment of suppurative thrombophlebitis is based on expert opinions. A recent retrospective study on patients with *S. aureus* suppurative thrombophlebitis showed that a duration of antibiotic therapy shorter than 28 days and the absence of anticoagulant therapy were associated with higher rates of treatment failure [172].

**R3.9—In critically ill patients** ***with catheter*-*related bloodstream infection demonstrated by comparative blood cultures*, with bacteraemia or local complications, the experts suggest that the catheter should be removed as soon as possible, regardless of the micro-organism or the clinical context.**

**EXPERT OPINION**

In the case of catheter-related bloodstream infection, the non-removal of the catheter is associated with mortality excess [148, 166], particularly if multidrug-resistant bacteria are involved. Indeed, the removal of the source of infection is one of the instrumental recommendations for the treatment of bacteraemia. This recommendation also applies to the treatment of candidaemia [173].

#### Initial antibiotic therapy

**R3.10—In the case of empirical antibiotic therapy for suspected CRI in a critically ill patient, the experts suggest prescription of an antibiotic (or a combination of antibiotics) targeting Gram-negative bacilli including** ***Pseudomonas aeruginosa,*** **in combination with treatment targeting Gram-positive cocci.**

**EXPERT OPINION**

The suspicion of CRI requires the administration of empirical antimicrobials before obtaining any results of microbiological culture. The treatment must comply with four principles: (a) the source of the infection should be controlled (i.e., catheter removal, and replacement by another catheter in another site if necessary), (b) the antimicrobials should be selected according to the local epidemiology and the patient’s colonisation, (c) the severity of the infectious episode and the presence of comorbidities should be taken into account, and (d) the treatment modalities should target effective plasma levels.

In 2016, data from French RÉA-RAISIN network showed that *S. aureus* (including 20% of methicillin-resistant strains), coagulase-negative staphylococci, *Enterobacteriaceae* (including 18.9% of strains producing extended spectrum beta-lactamases) and *Pseudomonas aeruginosa* were observed in 6%, 35%, 28% and 9.6% of positive CVC cultures, respectively.

When a CRI is suspected in an intensive care patient, the experts suggest of the administration of an antibiotic (or a combination of antibiotics) that targets Gram-negative bacilli including *P. aeruginosa*, and also Gram-positive cocci. Features such as shock, immunosuppression (particularly neutropenia or organ transplant), high prevalence of antibiotic resistance in the unit concerned and prior colonisation with a multi-drug resistant bacteria should be taken into account to select the appropriate antibiotic spectrum efficient against multi-drug resistant bacteria.

**R3.11—When a CRI is suspected in a critically ill patient, the experts do not recommend systematic initiation of empirical antifungal therapy.**

**EXPERT OPINION**

*Candida* spp. is involved in around 5% of CRI (RÉA-RAISIN 2017 report) and the risk of fungemia due to *Candida* spp. CRI is estimated around 1.3 per 1000 stays in intensive care patients [174]. Based on these very low percentages, the experts consider that this risk should be considered only in defined high-risk populations (i.e., circulatory shock, multiple organ dysfunction, prior treatment with broad-spectrum antibiotic therapy, and pre-existing fungal colonization) [2, 175–181].

**R3.12—The duration of the antibiotic therapy for documented catheter colonisation without bacteraemia depends on the species identified and the clinical setting in which the catheter was removed. The experts suggest the following** (Table 3):

- **no treatment is required in the absence of signs of infection. However, the clinical surveillance, with blood cultures even in the absence of fever, is required in the case of colonisation by** ***Staphylococcus aureus***, ***Candida*** **spp., and** ***Pseudomonas aeruginosa*** **and other non-fermenting Gram-negative bacilli.**
- **When the catheter was removed in a context of unexplained sepsis:**
  1. **In the case of colonisation by** ***S. aureus***, ***Candida*** **spp. or non-fermenting Gram-negative bacilli, the total duration of treatment should be 3 to 5** **days, in the absence of bacteraemia or complications**.
  2. **In the case of colonisation by coagulase-negative** ***S. aureus*** **or enterobacteria: no antibiotic therapy is required.**

**EXPERT OPINION**

Only 17% of patients with colonised catheters subsequently develop bacteraemia [2]. The risk of bacteraemia in patients in whom a colonised catheter was removed depends on several factors, including the patient’s underlying immunity, the presence or absence of thrombosis of the catheterized vein, the microbial species and probably the magnitude of the inoculum.

No randomised trial has identified those critically ill patients at increased risk of secondary bacteraemia after removal of a colonised catheter. Review of the literature [2] is limited by the observational nature of the studies, the absence of data concerning the conditions of catheter removal (presence or absence of fever, presence or absence of another site of infection) and the absence of systematic follow-up of patients after catheter removal. Three microbial species appear to be associated with an increased risk of secondary bacteraemia: *S. aureus*, *P. aeruginosa*, *A. baumannii*, and to lesser extent, *Candida* spp.

In this specific setting, the experts recommend to undertake systematically a treatment only in patients whom catheter was removed in the presence of unexplained fever, and those for whom the catheter culture identified significant levels of one of the 3 species indicated, at ≥ 10^3^ CFU/mL when the Brun–Buisson technique is used. As there are no data for defining the optimal duration of treatment, the experts propose at least 5 days of antibiotic therapy in the absence of bacteraemia or complications.

In patients with a confirmed catheter colonisation by *S. aureus*, without any concomitant positive peripheral blood culture, it is recommended to administer an anti-staphylococcal antibiotic therapy for 5 to 7 days [6]. This recommendation is supported only by a limited number of observational, retrospective studies with small sample sizes, rarely performed in intensive care units, and usually not controlled [142–144, 182–185]. In many cases, the definition of secondary bacteraemia used by several authors (> 24 h after catheter removal) does not allow catheter-related bloodstream infection to be distinguished from secondary bacteraemia. No data are available regarding arterial catheters colonised by *S. aureus* without a positive concomitant peripheral blood culture.

Three retrospective [136, 182, 186] and two prospective observational studies [187, 188] on bacterial colonisation recommended a duration of at least 3 days of antibiotic therapy, with at least one of the antibiotics adapted to antibiotic susceptibility testing. Three out of five studies, including two prospective studies, each targeting a particular microorganism, *P. aeruginosa* and *A. baumannii*, showed a marked reduction of bacteraemia rates.

Five retrospective studies [182, 189–192] focused on fungal colonisation were included in a meta-analysis [193] published in 2014. Particularly high mortality rates were reported in these studies (42%). These studies did not show any difference between the effect of antifungal therapy and of a simple surveillance of the patients on the development of fungaemia or deep candidiasis (5 studies), or on mortality (3 studies). Randomised therapeutic trials are required to further investigate this issue.

#### Treatment with bacterial documentation

**R3.13—The adequate duration of treatment of catheter-related bloodstream infection is 7** **days. However, the experts suggest a longer duration of treatment in the following settings:**

- **In** ***Staphylococcus aureus*** **or** ***Candida albicans*** **bloodstream infection, the total duration of treatment may be extended to 14** **days in the absence of secondary sites of infection or complications.**
- **In the presence of secondary sites of infection (endocarditis, septic metastases, osteomyelitis), or complications (i.e.**, **suppurative thrombophlebitis defined by the discovery of thrombosis in the catheterised vein and persistence of a bacteraemia for more than 72** **h), the total duration of treatment should be 4 to 6** **weeks** (Table 3).

**EXPERT OPINION**

No randomised trial has evaluated the impact of short-course antibiotic therapy (< 14 days) vs. prolonged treatment (> 14 days) on the recurrence rate, complication rate, and mortality in non-immunosuppressed patients. Available data, mostly derived from small-scale retrospective studies, are mainly focused on “uncomplicated” *S. aureus* central catheter-related infections [194–196]. The risk of secondary sites and recurrence appears to be increased in the case of short-course treatment (5–10%) [146, 194, 196–198]. Short-course treatment can be considered only in the absence of complication and after catheter removal. The duration of treatment for CRI due to Gram-negative bacilli, enterococci, or coagulase-negative staphylococci was not evaluated in reported studies.

**R3.14a—The experts suggest empirical antibiotic therapy including vancomycin when there is a suspicion of CRI, or when the patient or the unit’s ecological setting indicates a high risk of methicillin-resistant** ***Staphylococcus aureus*** **(MRSA) infection.**

**EXPERT OPINION**

**R3.14b—The experts suggest that teicoplanin should not be used as empirical antibiotic therapy for CRI.**

**EXPERT OPINION**

**R3.14c—Daptomycin should probably be used in the case of CRI with septic shock, acute renal failure and/or recent exposure to vancomycin (> 1** **week during the last 3** **months) or in the presence of a high local prevalence of methicillin-resistant** ***Staphylococcus aureus*** **(MRSA) with a vancomycin minimum inhibitory concentration (MIC) ≥ 1.5** **µg/mL.**

**GRADE 2+ STRONG CONSENSUS**

**R3.14d—The experts suggest that linezolid should not be used in the case of central venous catheter-related infection with septic shock.**

**EXPERT OPINION**

Patients with *S. aureus* CRI are at a high risk of haematogenous emboli, particularly when the catheter cannot be removed and/or when antibiotic therapy is inappropriate. Vancomycin is the antibiotic most commonly prescribed for infections due to methicillin-resistant *S. aureus* and/or coagulase-negative staphylococci. Various studies that compared the efficacy and safety of glycopeptides (vancomycin vs. teicoplanin) in *Staphylococcus* spp. bacteraemia (including MRSA) did not show any significant differences between both anti-infective agents [199, 200], although clinical isolates of *S. epidermidis* and *S. haemolyticus* with decreased susceptibility to teicoplanin have been reported [201].

Vancomycin is associated with lower clinical success rates for MRSA bacteraemia when strains have an MIC ≥ 1.5 mg/L [202, 203]. In a case–control study of cases of MRSA infection with a vancomycin MIC ≥ 1.5 mg/L, a higher survival rate was observed in the group of patients treated with daptomycin [204]. Multivariate analysis confirmed that renal failure and previous treatment with vancomycin were associated with significantly higher clinical failure rates. The impact on the results of treatment of bacteraemia due to coagulase-negative staphylocci with a vancomycin MIC ≥ 1.5 mg/L (measured by E test) remains poorly defined. Previous studies indicated that the efficacy of vancomycin is inferior to that of beta-lactam antibiotics (cefazolin or oxacillin) for the treatment of methicillin-susceptible *Staphylococcus aureus* bacteraemia [205–207], which would justify the inclusion of a beta-lactam antibiotic in the empirical treatment of all suspected cases of CRI. The mortality rate in a cohort of 5787 patients from 122 hospitals [208] treated with a beta-lactam antibiotic was 35% lower than that of patients treated with vancomycin (HR 0.65; 95% CI 0.52–0.80).

Given their absence of bactericidal activity, oxazolidinone such as linezolid could not be recommended.

Daptomycin is a lipopeptide antibiotic presenting a higher in vitro bactericidal activity against Gram-positive bacteria than vancomycin [209, 210]. The only randomised trial comparing daptomycin vs. vancomycin or a beta-lactam concluded that daptomycin was not less effective than vancomycin [211]. In a cohort study including 579 episodes of MRSA bacteraemia, no significant differences in terms of mortality were observed between patients treated with vancomycin or daptomycin (OR 1.42; 95% CI 0.83–2.44) [212]. However, in a recent study analysing the efficacy of daptomycin in 40 cancer patients with Gram-positive CRI (including *S. aureus*), and compared to a historical control group of 40 patients treated with vancomycin, negative cultures and clinical cure were achieved more rapidly in the group treated with daptomycin [213–215].

#### Complications

**R3.15—The experts suggest catheter removal and initiation of curative anticoagulant treatment in case of deep vein thrombosis associated with catheter-related infection.**

**EXPERT OPINION**

Catheter-related thrombosis is frequent and often asymptomatic. The pathogenesis of catheter-related thrombosis involves the activation of clotting pathways by the foreign material present in the bloodstream, vascular endothelial lesions, and the activation of endothelial cells [6]. Infection can also stimulate thrombus formation by aggravating clotting disorders. The presence of a mass of thrombus around the catheter increases the risk of microbial colonisation and bacteraemia [216]. CVC infection and thrombosis therefore appear to be bidirectionally related.

No randomised trial has evaluated the combination of treatment with both anticoagulant and antibiotic agents in the treatment of infected catheter-related thrombosis.

The treatment of non-infected catheter-related thrombosis requires the catheter removal [217] (which may be sufficient to allow resolution of the thrombus [218]) associated with anticoagulant therapy. In the case of infected catheter-related thrombosis, the Infectious Diseases Society of America (IDSA) guidelines (2009) [6] propose a treatment with heparin, based on a review of suppurative thrombophlebitis published in 2007 [219]. However, only one of the studies included in this meta-analysis involved infected catheter-related thrombosis [220].

By analogy with the treatment of non-catheter-related deep vein thrombosis [217, 221], anticoagulant therapy is proposed to treat infected catheter-related deep vein thrombosis. Treatment modalities according to the size and the impact of the thrombus have not been clearly defined.

### Paediatrics

**Paediatrics R.1—In paediatric intensive care, by analogy with adults, the experts suggest the use of 2% chlorhexidine-alcohol for skin disinfection prior to central venous catheter insertion in infants older than 1** **month and children.**

**EXPERT OPINION**

In 2007, the French Society for Hospital Hygiene (*Société Française d’Hygiène Hospitalière*) recommended a chlorhexidine–benzalkonium mixture in weak alcohol solution (Biseptine^®^) or chlorinated derivatives, for the antisepsis of healthy skin prior to CVC insertion in infants under the age of 1 month, and chlorhexidine-alcohol, rather than povidone-alcohol, in infants between the ages of 1 and 30 months. Clinical data showing the superiority of chlorhexidine-alcohol over povidone-alcohol and the optimal dose strength of chlorhexidine (0.5 or 2%) are not available in children. In adults, a multicentre randomised controlled trial demonstrated the superiority of 2% chlorhexidine compared to povidone alcohol solutions in terms of reduction of the bacteraemia, infection and colonization rates for all central venous and arterial catheters [26]. The cutaneous toxicity of chlorhexidine has mainly been observed during repeated applications in neonates (impregnated dressings) [222]. In a study of chlorhexidine dressings in a population of adults and children, the 2% concentration was shown to be more aggressive than 0.5% [223]. Finally, in the study conducted by Mimoz et al. higher rates of skin reactions were observed with chlorhexidine than with povidone [26]. More studies are therefore required to determine the safety of chlorhexidine, particularly in infants.

**Paediatrics R.2—An ultrasound-guided supraclavicular approach of the brachiocephalic vein should probably be preferred for central venous catheter insertion in infants and children, except in neonatology, to decrease the number of attempted cannulations and the immediate mechanical complications.**

**GRADE 2+ STRONG CONSENSUS**

In neonates, infants and children, the ultrasound-guided supraclavicular approach of the brachiocephalic vein (BCV) was recently proposed as an alternative to the classical infraclavicular approach of the subclavian vein, as it was associated with a very high success rate, a very low incidence of accidental arterial puncture and almost no cases of pneumothorax [224–226]. Ultrasound-guided cannulation of the BCV is associated with a higher rate of first attempt success and fewer cannulation attempts compared to other approaches [231, 232]. The accidental arterial puncture rate was not significantly different between internal jugular and subclavian vein cannulation [227, 228], but was lower for BCV cannulation [229]. The study by Lu et al. was not included in this analysis, as BCV cannulation was not ultrasound-guided in 2001 [230]. Very low pneumothorax rates were reported in the various studies (all cases of pneumothorax were observed with classical subclavian vein cannulation); no cases of pneumothorax were observed with the BCV in two case series and one comparative study [225, 226, 229]. Subclavian vein cannulation was associated with higher rates of guidewire misplacement and catheter malposition with a high level of evidence [227, 228, 231, 232], as access is more direct for femoral veins, right jugular veins and both brachiocephalic veins. Discordant results have been reported for catheter-related infections between jugular, subclavian and femoral veins. A retrospective study reported fewer infections (expressed per catheter-days) and fewer venous thromboses in the BCV group, but no details were provided for the other cannulation sites [229]. Femoral catheters are associated with a higher rate of venous thrombosis, with a low level of evidence, and a higher rate of catheter obstruction with a high level of evidence [228, 232, 233].

**Paediatrics R.3—In children, the experts prefer the radial artery to the femoral artery for arterial catheter insertion to decrease the risk of thrombosis.**

**EXPERT OPINION**

Femoral artery catheterisation is the reference technique for continuous blood pressure (BP) monitoring, as it reflects central BP (aorta) [234]. BP measured via a radial artery catheter is closely correlated with BP measured via a femoral artery catheter in the operating room and in paediatric intensive care, except in the particular setting of aortic clamping [235–237]. Catheter dysfunction was more frequent with radial artery catheters in children under the age of 13 months and weighing less than 8 kg in the study by Shin et al. [236]. However, the risk of thrombosis is significantly higher with femoral artery catheters in children [238]. This risk increases with increasing catheter bore and cannulation duration. The neonatal period is the only identified independent risk factor for thrombosis [238]. No paediatric study has compared the infectious risk of the two sites.

**Paediatrics R.4—In children, ultrasound-guided central venous catheter and arterial catheter insertion should be preferred to the use of anatomical landmarks.**

**GRADE 1+ STRONG CONSENSUS**

Randomised [239], observational [240–242] paediatric studies and one meta-analysis (including 8 randomised controlled trials (RCTs) [243] have reported homogeneous results that confirm the benefit of ultrasound-guided cannulation on the reduction of the cannulation failure rate or multiple attempts [239–243], the puncture rate [239, 242, 243] and the time required for cannulation [239, 242–244]. Complications related to cannulation (arterial puncture or haematoma) are generally decreased by a factor of 2 to 4 [239, 242–244], except in one study conducted by Leyvi which did not observe any improvement of the immediate complication rate with ultrasound guidance [241]. Similar improved results have been reported for arterial cannulation [245–247], with an even greater (four to fivefold) reduction of mechanical complications (haematoma or ischaemia). Several studies have evaluated out-of-plane ultrasound guidance for radial artery cannulation [245, 246]. Ultrasound guidance tends to be more beneficial in infants and young children (*p* = 0.07) [246]. No data are available in the literature to determine the value of ultrasound guidance for prevention of complications related to maintenance of central venous and arterial catheters.

**Paediatrics R.5—Antimicrobial (antiseptic or antibiotic)-impregnated CVCs should probably not be used in children to decrease the incidence density of bacteraemia (expressed per 1000 catheter-days) when standard preventive measures are sufficient to obtain low incidence densities of catheter-related infection.**

**GRADE 2− STRONG CONSENSUS**

A randomised trial showed that the use of rifampin- and minocycline-impregnated catheters improved the incidence density (ID) of catheter-related infections in English paediatric intensive care units (8.24 vs. 3.31/1000 catheter-days, HR 0.4; 95% CI [0.17–0.97]) [248]. This benefit was also confirmed in a retrospective study of burns patients (14 vs. 8/1000 catheter-days) [249]. Incidence densities were particularly high in these 2 studies, as reflected by the values observed in control groups. In contrast, two other studies failed to demonstrate this benefit, an observational study [250] and a randomised trial [251] using the same type of catheter with a lower ID in the control group (3.46 vs. 3.62/1000 catheter-days, *p* > 0.99) [251]. The small number of CRI reported in these studies does not suggest the emergence of resistance of the bacteria concerned during the use of antibiotic-impregnated catheters. Further randomised trials are necessary to more precisely target the population likely to benefit from these catheters.

**Paediatrics R.6—Heparin-bonded CVCs should probably not be used in children to reduce the risk of occlusion or thrombosis.**

**GRADE 2− STRONG CONSENSUS**

Heparin-bonded CVCs could decrease the thrombogenic risk of the device and therefore reduce platelet aggregation, as well as the adhesion of bacteria such as staphylococci. The Cochrane group published a review of the literature in 2014 to determine the effects of heparin-bonded CVCs on the risk of thrombosis, occlusion, infection and local risks [33]. By pooling the results of two randomised controlled trials comparing heparin-bonded CVCs versus standard CVCs, Shah et al. showed a non-significant RR of thrombosis (determined clinically or by Doppler ultrasound) of 0.31 (95% CI [0.01–7.68]) and a risk of occlusion (preventing injection) of 0.22 (0.07–0.72) only for catheters maintained in place for more than 7 days (only one study was analysed). In a more recent randomised clinical trial, Gilbert et al. did not find any difference between heparin-bonded CVCs and standard CVCs for prevention of thrombosis (25 vs. 21%, *p* = 0.36) or infection (8.78 vs. 8.24 CRI/1000 catheter-days), despite that more than one-half of cases in both groups used the femoral site, known to be the most thrombogenic [248].

**Paediatrics R.7—The experts propose the creation of a quality improvement programme in children to decrease the rate of catheter-related infections.**

**EXPERT OPINION**

The Institute for Healthcare Improvement recommends a five-step programme for the prevention of CRI: hand hygiene, maximal barrier precautions upon insertion, chlorhexidine skin antisepsis, optimal catheter site selection, and daily review of line necessity, with prompt removal of unnecessary lines. Although the individual value of these measures appears to be less obvious in children, the existence of a quality improvement programme combining these measures allows a reduction of the incidence density (ID) of CRI in paediatric intensive care units. In the meta-analysis performed by Ista et al. the ID in paediatric intensive care units decreased from a median of 5.9/1000 catheter-days (range: 2.6–31.1) to 4.3/1000 catheter-days (range: 0–16.5). An increased effect was observed in the case of an initial ID greater than or equal to 5/1000 catheter-days [132]. A programme including recommendations on catheter placement, maintenance and education may contribute to achieving a “Zero infection” target [252]. Future studies should address the various cannulation methods of the IHI bundles and the impact of compliance with the prevention programme on the course of CRI and healthcare-related infections.

**Paediatrics R.8a—Chlorhexidine-impregnated dressings can be used on central venous catheter sites in children when standard prevention measures are not sufficient to decrease the catheter-related infection rate. These dressings are not recommended in preterm neonates.**

**EXPERT OPINION**

**Paediatrics R.8b—No paediatric recommendation can be issued concerning the use of chlorhexidine-impregnated dressings on arterial catheter sites.**

**NO RECOMMENDATION**

Six studies have evaluated the benefits and safety of chlorhexidine-impregnated dressings compared to standard dressings of CVC in children. Four of these trials were randomised controlled trials [35, 253–255], one was an observational study [256] and one was a retrospective study [257]. They were conducted in various units, such as polyvalent paediatric intensive care units [253, 256], cardiovascular intensive care units [35], neonatal intensive care units [255], oncology and haematology units [254] or haemodialysis units [257]. They sometimes included a mixed paediatric and neonatal population [35, 256]. Apart from several cases of contact dermatitis that resolved spontaneously after removal of the impregnated dressing, these dressings were well tolerated, locally and systemically [35, 254, 255]. More specifically, no systemic effects were observed. The majority of local reactions were observed in neonates [35], and serious necrotic lesions were reported in very low birthweight (< 1500 g) preterm neonates [255]. Some trials [253, 254] showed a tendency towards lower central line-associated bloodstream infection or local infection rates with chlorhexidine-impregnated dressings. However, these decreased rates never reached the statistical significance [35, 253–257]. Chlorhexidine-impregnated dressings were associated with lower CVC colonisation rates in all trials in which this effect was investigated [35, 253, 255], bearing in mind that CVC colonisation is strongly associated with CRI [134]. Further and more powerful studies are required to demonstrate a significant reduction of the CRI rate, as well as the direct and indirect costs of impregnated dressings in children and the unscheduled dressing change rate, which have not yet been studied in children. No published data are available concerning chlorhexidine-impregnated dressings for arterial catheters.

## Supplementary information

**Additional file 1.** Pediatrics R1 Chlorhexidine-alcohol disinfection. Pediatrics R2 and R4 US and site of insertion. Pediatrics R3 radial vs. femoral artery access. Pediatrics R5 impregnated impregnated CVCs. Pediadrics R6 heparin bonded CVCs. Pediatrics R7 continuous quality improvement program. Pediatrics R8 CHG dressings CVCs and arterial catheters. Adults and pediatrics Prevention R 1-1 and 1-2 subclavian vs. Internal jugular vs. femoral. R 1-3 alc-CHG vs. alc-PVI. R 1-4 1 step vs. 4 steps desinfections. R 1-5 antiseptic and antibiotic impregnated catheters. R1-6 heparin bonded CVCs. R 1-7 CHG dressings vs. transparent dressings. R 1-8 dressing change frequencies. R 1-9 R1-10 R 1-11 R1-12 US and site of insertion. Surveillance R 2.1 R2.2 surveillance network. R 2-2 quality improvement program. R2-3 culture of catheters Catheter related infection R3-1 R3-4 blood culture. R3-2 R3-3a R3-3b persistent bacteraemia : R 3-5 R3-6 R3-7 R3-9 R3-15 Catheter removed. R3-8 R3-10 R3-12 R3-13 duration of antibiotics. R3-11 antifungal therapy R3-14 antibiotic therapy.

## Data Availability

Not applicable.

## Abbreviations

AC: Arterial catheter
ADARPEF: Association Des Anesthésistes Réanimateurs Pédiatriques d’Expression Française
BCV: Brachiocephalic vein
BMI: Body mass index
BP: Blood pressure
CDC: Center for Disease Prevention and Control
CFU: Colony-forming units
CHD: Cardiomyopathy Heart Disease
CHG: Chlorhexidine
CoNS: Coagulase-negative *Staphylococci*
GPC: Gram Positive Cocci
CRI: Catheter-related infection
CVC: Central Venous Catheter
ECDC: European Centre for Disease and Control
ESBLPE: Extended-spectrum beta-lactamase-producing Enterobacteriaceae
ESBLs: Extended-spectrum beta-lactamases
GRADE methodology: *Grade of Recommendation Assessment, Development and Evaluation methodology*
GFRUP: Groupement Francophone de Réanimation et Urgences Pédiatriques
ID: Incidence density
IDSA: Infectious Diseases Society of America
IE: Infective endocarditis
HR: Hazard Ratio
IHI: Institute for Healthcare Improvement
MDR: Multidrug-resistant bacteria
MIC: Minimum Inhibitory Concentration
MRSA: Methicillin-resistant *Staphylococcus aureus*
PICO: *Patient Intervention Comparison Outcome*
RCT: Randomized controlled trial
SBP: Systolic blood pressure
SIRS: Systemic Inflammatory Response Syndrome
SRLF: Société de Réanimation de Langue Française
THC: Temporary haemodialysis catheter
TOE: Transoesophageal echocardiography
TTE: Transthoracic echocardiography
