# Supplementary material for: Expert consensus-based clinical practice guidelines management of intravascular catheters in the intensive care unit
Source: Ann Intensive Care. 2020 Sep 7;10:118. doi: 10.1186/s13613-020-00713-4 (PMC7477021; doi:10.1186/s13613-020-00713-4)
Supplement: Supplementary file 1 — Additional file 1. Pediatrics R1 Chlorhexidine-alcohol disinfection. Pediatrics R2 and R4 US and site of insertion. Pediatrics R3 radial vs. femoral artery access. Pediatrics R5 impregnated impregnated CVCs. Pediadrics R6 heparin bonded CVCs. Pediatrics R7 continuous quality improvement program. Pediatrics R8 CHG dressings CVCs and arterial catheters. Adults and pediatrics Prevention R 1-1 and 1-2 subclavian vs. Internal jugular vs. femoral. R 1-3 alc-CHG vs. alc-PVI. R 1-4 1 step vs. 4 steps desinfections. R 1-5 antiseptic and antibiotic impregnated catheters. R1-6 heparin bonded CVCs. R 1-7 CHG dressings vs. transparent dressings. R 1-8 dressing change frequencies. R 1-9 R1-10 R 1-11 R1-12 US and site of insertion. Surveillance R 2.1 R2.2 surveillance network. R 2-2 quality improvement program. R2-3 culture of catheters Catheter related infection R3-1 R3-4 blood culture. R3-2 R3-3a R3-3b persistent bacteraemia : R 3-5 R3-6 R3-7 R3-9 R3-15 Catheter removed. R3-8 R3-10 R3-12 R3-13 duration of antibiotics. R3-11 antifungal therapy R3-14 antibiotic therapy. [file 13613_2020_713_MOESM1_ESM.docx]

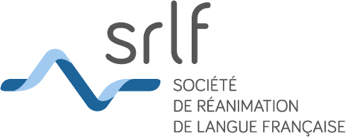


**Expert consensus-based clinical practice guidelines**

**Management of intravascular catheters in the intensive care unit**

**SRLF-sponsored CPG**

***Société de ICU de Langue Française***

**in collaboration with the GFRUP and the ADARPEF**

***Groupement Francophone de ICU et Urgences Pédiatriques***

***Association des Anesthésistes Réanimateurs Pédiatriques d’Expression Française***

**Authors:** Jean-François Timsit^1^, Julien Baleine^2^, Lyess Bernard^3^, Silvia Calvino-Gunther^4^, Michael Darmon^5^, Jean Dellamonica^6^, Eric Desruennes^7^, Marc Leone^8^, Alain Lepape^9^, Olivier Leroy^10^, Jean-Christophe Lucet^11^, Zied Merchayes^12^, Olivier Mimoz^13^, Benoit Misset^14^, Jean-Jacques Parienti^15^, Jean-Pierre Quenot^16^, Antoine Roch^17^, Matthieu Schmidt^18^, Michel Slama^19^, Bertrand Souweine^20^, Jean-Ralph Zahar^21^, Walter Zingg^22^, Laetitia Bodet-Contentin^23^, Virginie Maxime^24^.

**Corresponding author:**

**Virginie Maxime** [virginie.maxime@aphp.fr](mailto:virginie.maxime@aphp.fr)

ICU médicochirurgicale Hôpital Raymond Poincaré 9230 Garches

**ADDITIONAL MATERIALS**

Pediatrics R2-4 US and site of insertion 3

Pediatrics R2 and R4 US guided pediatrics 6

Pediatrics R3 arterial vs femoral access 9

Pediatrics R5 impregnated CHG dressings 12

Pediatrics R7 continuous quality improvement program 17

R 1-1 and 1-2 subclavian vs Internal jugular vs femoral 18

R 1-3 alc-CHG vs alc-PVI 20

R 1-4 1 step vs 4 steps desinfections 21

R 1-5 antiseptic and antibiotic impregnated catheters 22

R 1-7 CHG dressings vs transparent dressings 24

R 1-8 dressing change frequencies 28

R 1-9 R1-10 R 1-11 PICO question US insertion adult 30

R 2.1 R2.2 tables 41

R 2-2 quality improvement program 48

R 3.5 Guidewire exchange RFE VDVD 57

| **Puncture adverse events** | | | | | | | | | | | | | | | | |
| --- | --- | --- | --- | --- | --- | --- | --- | --- | --- | --- | --- | --- | --- | --- | --- | --- |
| **Quality evaluation** | | | | | | | | **Number of patients** | |  |  |  | **Effect** | | **Quality** | **Importance** |
| **Number of studies** | **Type of study** | **Risk of bias** | **Inconcistencies** | | **Indirect data** | **Imprecision** | **publication bias** | **brachiocephalic vein** | **other sites of insertion** | **internal jugular** | **sub clavian** | **femoral** |  |  |  |  |
| **Sucess first puncture** | |  |  | |  |  |  |  |  |  |  |  |  |  |  |  |
| Lu 2006 | observational | no | no | | serious^a,b^ | no | no | 38/45 (84%) |  |  | 36/46 (78%) |  | RR 1,07 [0,88-1,31] | NS | ⊕⭘⭘⭘ **VERY LOW** | moderate |
| Byon 2013 | prospective randomised | no | no | | serious^a^ | no | no | 32/49 (65%) |  |  | 24/49 (49%) |  | RR 1,33 [0,94-1,89] | p 0,10 | ⊕⊕⊕⭘ **MODERATE** | moderate |
| Oulego 2016 | prospective observational | no | no | | no | no | no | 16/22 (73%) |  | 9/24 (27%) |  |  | RR 1,93 [1,09-3,45] | p<0,05 | ⊕⊕⭘⭘ **LOW** | moderate |
| **Arterial puncture** |  |  |  | |  |  |  |  |  |  |  |  |  |  |  |  |
| Casado-Flores 2001 | prospective observational | no | no | | no | serious^c^ | no |  |  | 0/10 | 12/235 | 4/63 |  | NS | ⊕⭘⭘⭘ **VERY LOW** | important |
| Lu 2006 | observational | no | no | | serious^a,b^ | no | no | 5/45 (11%) |  |  | 1/46 (2%) |  | RR 5,11 [0,6-42] | NS | ⊕⭘⭘⭘ **VERY LOW** | important |
| Karapinar 2007 | prospective observational | no | very serious d | | no | no | no |  |  | 6/84 (7%) | 17/119 (14%) | 10/166 (6%) | RR 2,23 [1,16-4,26] (subclav vs other sites) | p<0,05 | ⊕⭘⭘⭘ **VERY LOW** | important |
| Camkiran 2016 | prospective randomised | no | very serious d | | no | no | no |  |  | 11/139 (8%) | 3/141 (2%) |  | RR 3,72 [1,06-13,04] | p<0,05 | ⊕⊕⭘⭘ **LOW** | important |
| Habas 2018 | observational | no | no | | no | no | no | 2/147 (1,4%) | 8/110 (7,3%) |  |  |  | RR 0,18 [0,04-0,86] (VBC vs other sites) | p<0,01 | ⊕⊕⭘⭘ **LOW** | important |
| **Pneumothorax** |  |  |  | |  |  |  |  |  |  |  |  |  |  |  |  |
| Casado-Flores 2001 | prospective observational | serious | no | | no | serious^c^ | no |  |  | 0/10 | 6/235 | 0/63 |  |  |  | important |
| Karapinar 2007 | prospective observational | serious | no | | no | no | no |  |  | 0/84 | 3/119 | 0/166 | NS (subclavian vs other sites) | few events |  | important |
| Habas 2018 | observational | serious | no | | no | no | no | 0/147 | 1/110 (0,9%) |  |  |  | NS |  |  | important |
| **wrong route malposition** | |  |  | |  |  |  |  |  |  |  |  |  |  |  |  |
| Casado-Flores 2001 | prospective observational | no | no | | no | serious^c^ | no |  |  | 2/10 | 38/235 (16%) | 3/63 | RR 2,36 (subclavian vs other sites) [0,96-5,78] | p<0,05 | ⊕⊕⭘⭘ **LOW** | moderate |
| Karapinar 2007 | prospective observational | no | no | | no | no | no |  |  | 2/84 | 19/119 | 6/166 | RR 4,99 (subclavian vs other sites) [2,24-11,06] | p < 0,0001 | ⊕⊕⊕⊕ **HIGH** | moderate |
| Byon 2013 | prospective randomised | no | no | | serious^a^ | no | no | 0/49 |  |  | 10/49 |  | RR infinite | p < 0,001 | ⊕⊕⊕⊕ **HIGH** | moderate |
| Camkiran 2016 | prospective randomised | no | no | | no | no | no |  |  | 2/139 (1,4%) | 17/141 (12%) |  | RR 8,38 [1,97-35] | p < 0,001 | ⊕⊕⊕⊕ **HIGH** | moderate |
| Habas 2018 | observational | no | no | | no | no | no | 8/147 (5,4%) | 5/110 (4,5%) |  |  |  | RR 0,84 [0,28-2,48] | NS | ⊕⊕⭘⭘ **LOW** | moderate |
| **CLABSI** |  |  |  | |  |  |  |  |  |  |  |  |  |  |  |  |
| Casado-Flores 2001 | prospective observational | no | no | | no | serious^c^ | no |  |  | 0/10 | 10/235 | 3/63 | RR 1,17 [0,33-4,11] (femoral vs other sites) | NS | ⊕⭘⭘⭘ **VERY LOW** | critical |
| Reyes 2012 | observational | no | no | | no | no | no |  |  | 5,3/1000 days | 5,2/1000 days | 5,8/1000 days | NS |  | ⊕⊕⊕⭘ **MODERATE** | critical |
| Camkiran 2016 | prospective randomised | serious^f^ | no | | no | no | no |  |  | 6,9/1000 days | 0/1000 days |  | p < 0,001 |  | ⊕⊕⊕⭘ **MODERATE** | critical |
| Habas 2018 | observational | no | no | | no | no | no | 5/147 (3,4%), 2,8/1000 days | 10/110 (9,1%), 8,56/1000 days |  |  |  | RR 0,37 (VBC vs other sites), p=0,054 | expressed in catheter-days: p<0,001 | ⊕⊕⊕⭘ **MODERATE** | critical |
| **Thrombosis** |  |  |  | |  |  |  |  |  |  |  |  |  |  |  |  |
| Beck 1998 | prospective observational | serious^f^ | no | | no | no | no |  |  | 4/44 | 3/9 | 5/23 | NS |  | ⊕⭘⭘⭘ **VERY LOW** | critical |
| Casado-Flores 2001 | prospective observational | serious | no | | no | serious^c^ | no |  |  | 0/10 | 2/235 (0,9%) | 4/63 (6,3%) | RR 7,78 [1,46-41] (femoral vs other sites) | p<0,05 | ⊕⊕⭘⭘ **LOW** | critical |
| Male 2003 | prospective observational | serious^g,h^ | no | | serious^i^ | no | no |  |  | 7/35 (20%) | 22/50 (44%) |  | RR 2,2 [1,06-4,58] (SC vs IJV) | p<0,05 | ⊕⭘⭘⭘ **VERY LOW** | critical |
| Karapinar 2007 | prospective observational | very few events | no | | no | no | no |  |  | 1/84 | 3/119 | 4/166 | NS |  | ⊕⭘⭘⭘ **VERY LOW** | critical |
| Gray 2012 | observational | serious^h^ | no | | no | serious^h^ | no |  | IJV + S/C = 14/175 | 10/111 (9%) | 4/64 (6%) | 32/155 (21%) | RR 2,58 [1,43-4,65] (femoral vs other sites) | p < 0,001 | ⊕⊕⭘⭘ **LOW** | critical |
| Habas 2018 | observational | no | no | | no | no | no | 4/147 (2,7%) | 11/110 (10%) |  |  |  | RR 0,27 [0,09-0,83] VBC vs other sites) | p<0,05 | ⊕⊕⭘⭘ **LOW** | critical |
| **catheter obstruction** | |  |  | |  |  |  |  |  |  |  |  |  |  |  |  |
| Casado-Flores 2001 | prospective observational | no | no | | no | serious^c^ | no |  |  | 2/10 (20%) | 14/235 (6%) | 15/63 (24%) | RR 3,65 [1,91-6,97] (femoral vs other sites) | p<0,0001 | ⊕⊕⭘⭘ **LOW** | moderate |
| Karapinar 2007 | prospective observational | no | no | | no | no | no |  |  | 3/84 (3,6%) | 5/119 (4,2%) | 18/166 (10,8%) | RR 2,75 [1,23-6,17] (femoral vs other sites) | p<0,05 | ⊕⊕⊕⭘ **MODERATE** | moderate |
|  |  |  |  | |  |  |  |  |  |  |  |  |  |  |  |  |
| a: unique operator | |  |  | |  |  |  |  |  |  |  |  |  |  |  |  |
| b: no US guided |  |  |  | |  |  |  |  |  |  |  |  |  |  |  |  |
| c: few jugular accesses | |  |  | |  |  |  |  |  |  |  |  |  |  |  |  |
| d: heterogeneous results in 2 studies | | | |  |  |  |  |  |  |  |  |  |  |  |  |  |
| e: few events |  |  |  | |  |  |  |  |  |  |  |  |  |  |  |  |
| f: no ITT analysis (CLABSI) | |  |  | |  |  |  |  |  |  |  |  |  |  |  |  |
| g: small numbers |  |  |  | |  |  |  |  |  |  |  |  |  |  |  |  |
| h: mixt of puncture and cut-down | |  |  | |  |  |  |  |  |  |  |  |  |  |  |  |
| i: secondary analysis of a study on asparaginase and thrombosis | | | | |  |  |  |  |  |  |  |  |  |  |  |  |

|  | **US guided vs no paediatrics** | | |  |  |  |  |  |  |  |  |  |  |  |  |
| --- | --- | --- | --- | --- | --- | --- | --- | --- | --- | --- | --- | --- | --- | --- | --- |
|  |  |  |  |  |  |  |  |  |  |  |  |  |  |  |  |
|  | **Quality evaluation** | | | | | | | | | **Number of catheters** | | **Effect** | | **Quality** | **Importance** |
|  | **Number of studies** | **author** | **type of study** | **country** | **Plan experience / execution** | **inconsistencies** | **indirect data** | **Imprecision** | **Publication bias** | **US guided** | **control** | **RR(CI95%)** | **WMD** |  |  |
| **VEIN** | Mechanical complications (arterial puncture) | | | | | | | | | | | | | | |
|  | 8 | Lau and Camberlain | Meta-analysis |  |  | Y (I² = 64,5) |  |  |  | 367 | 393 | 0,36 (0,12-1,09) | 4,6% vs 16,8%) | **MODERATE** |  |
|  |  | Zanolla and al | RCT | Brazil | S | N | N | S | N | 23 | 28 |  | 4,3% vs 39,3% | **LOW** |  |
|  |  | Leyvi and al 2005 | retrospective | USA |  |  |  |  |  | 47 | 102 |  | 6,4% vs 4,9% (p NS) | **LOW** |  |
|  |  | Froehlich and al 2009 | prospective no randomised | USA |  |  |  |  |  | 119 | 92 | 0,39 | 8,5 vs 19,4 % (p=0,03) | **LOW** |  |
|  |  | Oulego-Erroz and al 2018 | prospective observational | Spain |  |  |  |  |  | 133 | 133 | 0,47 (0,24-0,91) | 12 vs 22,5% | **MODERATE** |  |
|  | Time required for cannulation | | | | | | | | | | | | | | |
|  | 4 | Lau and Camberlain | meta-analysis |  |  | Y (I² = 83,8) |  |  |  | 138 | 153 | -1,12 min (-2,6 to 0,35) | | **MODERATE** |  |
|  |  | Zanolla and al | RCT | Brazil | S | N | N | S | N | 23 | 28 |  | 108,5 s vs 246 s(p=0,002 | **LOW** |  |
|  |  | Froehlich and al 2009 | prospective no randomised | USA |  |  |  |  |  | 119 | 92 |  | 150 vs 269 s (p=0,14) | **LOW** |  |
|  |  | Oulego-Erroz and al 2018 | prospective observational | Spain |  |  |  |  |  | 133 | 133 | 90 (-51,+232) | 60 vs 90s | **MODERATE** |  |
|  | Success/failure (ou > 3 punctures) cannulation | | | | | | | | | | | | | | |
|  | 8 | Lau and Camberlain | meta-analysis |  |  | Y (I² = 83,4) | N |  |  | 367 | 393 | 1,32 (1,1-1,58) | 92% vs 68% | **MODERATE** |  |
|  |  | Zanolla et al | RCT | Brazil | S | N | N | S | N | 23 | 28 |  | 95,7% vs 71,4% p =0,031 | **LOW** |  |
|  |  | Leyvi and al 2005 | retrospective | USA |  |  |  |  |  | 47 | 102 |  | 91,5 vs 72,5 % (p=0,01) | **LOW** |  |
|  |  | Froehlich and al 2009 | prospective no randomised | USA |  |  |  |  |  | 119 | 92 |  | 79,8 vs 60,2% (p=0,32) | **LOW** |  |
|  |  | Oulego-Erroz and al 2018 | prospective observational | Spain |  |  |  |  |  | 133 | 133 | 1,64 (0,93-2,91) | 80,4 vs 71,4 % | **MODERATE** |  |
|  | Infection rate | | | | | | | | | | | | | | |
|  | not found |  |  |  |  |  |  |  |  |  |  |  |  |  |  |
|  |  |  |  |  |  |  |  |  |  |  |  |  |  |  |  |
|  | Duration cannulation | | | | | | | | | | | | | | |
|  | not found |  |  |  |  |  |  |  |  |  |  |  |  |  |  |
|  |  |  |  |  |  |  |  |  |  |  |  |  |  |  |  |
|  | Pain/comfort patient | | | | | | | | | | | | | | |
|  | not found |  |  |  |  |  |  |  |  |  | |  |  |  |  |
|  |  |  |  |  |  |  |  |  |  |  |  |  |  |  |  |
|  |  |  |  |  |  |  |  |  |  |  |  |  |  |  |  |
|  | Number of attempted cannulations | | | | | | | | | | | | | | |
|  | 5 | Lau and Camberlain | Meta-analysis |  |  | Y (I² = 66,7) |  |  |  | 226 | 228 | -1,26 (-1,71 to -0,81) | | **MODERATE** |  |
|  |  | Zanolla and al | RCT | Brazil | S | N | N | S | N | 23 | 28 |  | 1 vs 3 (p < 0,001) | **LOW** |  |
|  |  | Oulego-Erroz and al 2018 | prospective observational | Spain |  |  |  |  |  | 133 | 133 | -0,51 (-1,01,-0,03) | 2 vs 2 | **MODERATE** |  |
| **ARTERY** | Mechanical complications | | | | | | | | | | | | | | |
|  |  | Anantasit and al 2017 | RCT | Thailand | N | N | Y | N | N | 41 | 43 |  | 12,2 vs 53,5 % (p<0,001) | **MODERATE** | compl = haematoma |
|  |  | Siddik-Sayyid and al 2016 | RCT | Liban | N | N | Y | N | N | 53 | 53 |  | 0 vs 0 |  |  |
|  | 2 | Aouad-Maroun and al 2016 | Meta-analysis |  |  | I² = 0% |  | Y | Y | 111 | 111 | 0,2 (0,07-0,6) | 3,1 vs 15,3 % | **MODERATE** |  |
|  | Time required for cannulation | | | | | | | | | | | | | | |
|  |  | Anantasit and al 2017 | RCT | Thailand | N | N | Y | N | N | 41 | 43 | 3,26 (1,8-5,89 | 3,3 vs 10,4 min (p<0,001) | **MODERATE** |  |
|  |  | Siddik-Sayyid and al 2016 | RCT | Liban | N | N | Y | N | N | 53 | 53 | 119 s(26-212) | 301 vs 420 s (p=0,012) | **MODERATE** |  |
|  |  | Schwemmer and al 2006 | RCT | Germany | Y |  |  | Y |  | 15 | 15 |  | 65 vs 151 s (p < 0,05) | **MODERATE** | Small workforce, few events, drawing of lot |
|  | Success/failure (ou > 3 punctures) cannulation | | | | | | | | | | | | | | |
|  |  | Anantasit and al 2017 | RCT | Thailand | N | N | Y | N | N | 41 | 43 | 2,03 (1,13-3,64) | 80,5 vs 39,5% (p<0,01) | **MODERATE** |  |
|  |  | Siddik-Sayyid and al 2016 | RCT | Liban | N | N | Y | N | N | 53 | 53 | 2,18 (0,95-5,01) | 75 vs 58% (p=0,06) | **MODERATE** |  |
|  | 2 | Aouad-Maroun and al 2016 | Meta-analysis |  |  |  |  | Y | Y | 67 | 67 | 1,78 (1,25-2,51) | 61,6 vs 35,8% (p=0,002) | **MODERATE** | downgraded for small number of events and performance bias |
|  | Number of punctures | | | | | | | | | | | | | | |
|  |  | Siddik-Sayyid and al 2016 | RCT | Liban | N | N | Y | N | N | 53 | 53 | -1(-1,72,-0,28) | 1 vs 2 (p=0,003) | **MODERATE** |  |
|  |  | Schwemmer and al 2006 | RCT | Germany | Y |  |  | Y |  | 15 | 15 |  | 1,3 vs 2,3 (p<0,05) | **MODERATE** |  |
|  |  | Ishii and al 2013 | RCT |  |  |  |  |  |  |  |  |  | 1 vs 2 (p<0,01) | |  |
|  | Success 1st puncture | | | | | | | | | | | | | | |
|  |  | Anantasit and al 2017 | RCT | Thailand | N | N | Y | N | N | 41 | 43 | N.S | 60,6 vs 29,4% (p=0,072) | **MODERATE** |  |
|  | 4 | Aouad-Maroun and al 2016 | Meta-analysis |  |  | I² = 23% |  | Y | Y | 198 | 206 | 1,96 (1,34-2,85) | 40,9 vs 20,9% | **MODERATE** | downgraded for small number of events and performance bias and selective reporting bias |
|  |  | Siddik-Sayyid and al 2016 | RCT | Liban | N | N | Y | N | N | 53 | 53 | 2,54 (1,11-5,82) | 45 vs 25% (p=0,025) | **MODERATE** |  |

| **arterial vs femoral catheters** | |  |  |  |  |  |  |  |  |  |  |  |  |  |  |
| --- | --- | --- | --- | --- | --- | --- | --- | --- | --- | --- | --- | --- | --- | --- | --- |
|  |  |  |  |  |  |  |  | **Number of patients** | |  | **Effects** | | **Quality** | **Importance** | comments |
| **Authors** | **type of study** | **study plan** | **inconsistencies** | **imprecision** | **risk of bias** | **publication bias** | **Complexity** | **Radial** | **Femoral** | **BP radial** | **BP femoral** | **P** |  |  |  |
| **Fiability of arterial pressure measurement** | | |  |  |  |  |  |  |  |  |  |  |  |  |  |
| Cho^2^ | retrospective |  |  |  |  |  |  | 121 | 121 | no diff in BPM measurement |  | NS | **⨁◯◯◯ Very low** | **important** | BPM : No significant difference between the 2 catheters apart from aortic clamping (pre clamping AO and post op in CPICU), lower radial BPM during clamping |
| Shin^3^ | prospective | poor | No | Yes | Yes | No |  | 32 | 32 | no diff in BPM, systolic BP and diastolic BP measurements |  | NS | **⨁◯◯◯ Very low** | **important** | BPM, BPS, BPD no difference before start of the transplant |
| Cetin^4^ | prospective | poor | No | No | Yes | No | No | 45 | 45 | no diff in BPM, systolic BP and diastolic BP measurements |  | <0,001 | **⨁◯◯◯ Very high** | **important** | BPM, BPS, BPD perfectly correlated with all stages of cardiac surgery with a femoral radial gradient most often < 5mmHg |
|  |  |  |  |  |  |  |  |  |  |  |  |  |  |  |  |
|  |  |  |  |  |  |  |  |  |  |  |  |  |  |  |  |
|  |  |  |  |  |  |  |  |  | |  |  | |  |  |  |
| **Authors** | **type of study** | **study plan** | **inconsistencies** | **imprecision** | **risk of bias** | **publication bias** | **Complexity** | **Radial** | **Femoral** | **Radial rate** | **Femoral rate** | **P** |  |  |  |
| **Ischemia or thrombosis** | |  |  |  |  |  |  |  |  |  |  |  |  |  |  |
| Cho^2^ | retrospective |  |  |  |  |  |  | 121 | 121 | 0,00% | 0,00% | NS | **⨁◯◯◯ Very low** | **no important** | KTA duration in place not specified, no systematic search for thrombosis, no blinding, no randomization |
| Brotschi^5^ | prospective observational | poor | No | No | Yes | No | No | 418 | 137 | 0,00% | 13,13% | <0,05 | **⨁⨁◯◯ Low** | **Crucial** | FR femoral thrombosis: low flow, NN, high Hct, low weight in univariate and multivariate only age is significant; doppler syst in femoral KTA but not in radial KTA |
| **Malfunction** |  |  |  |  |  |  |  |  |  |  |  |  |  |  |  |
| Shin^3^ | prospective | poor | No | Yes | Yes | No | No | 32 | 32 | 8,57% | 0% |  | **⨁⨁◯◯ Low** | **important** | dysfunction KTA radial < 8kg et <13 month |
| **Catheter related infections** | |  |  |  |  |  |  |  |  |  |  |  |  |  |  |
| Cho^2^ | retrospective |  |  |  |  |  |  | 121 | 121 | 0,00% | 0,00% |  | **⨁◯◯◯ Very low** | **not important** |  |
| **references** |  |  |  |  |  |  |  |  |  |  |  |  |  |  |  |
| 3 | Shin YH, Kim HY, Kim YR, Yoon JS, Ko JS, Gwak MS, et al. The Comparison of Femoral and Radial Arterial Blood Pressures During Pediatric Liver Transplantation. Transplant Proc. 2013 Jun;45(5):1924–7. | | | | | | | | | | |  |  |  |  |
| 4 | Cetin S, Pirat A, Kundakci A, Camkiran A, Zeyneloglu P, Ozkan M, et al. Radial Mean Arterial Pressure Reliably Reflects Femoral Mean Arterial Pressure in Uncomplicated Pediatric Cardiac Surgery. J Cardiothorac Vasc Anesth. 2014 Feb;28(1):76–83. | | | | | | | | | |  |  |  |  |  |
| 5 | Brotschi B, Hug MI, Latal B, Neuhaus D, Buerki C, Kroiss S, et al. Incidence and predictors of indwelling arterial catheter-related thrombosis in children: Arterial thrombosis in children. J Thromb Haemost. 2011 Jun;9(6):1157–62. | | | | | | | | | |  |  |  |  |  |
| 2 | Cho HJ, Lee SH, Jeong IS, Yoon NS, Ma JS, Ahn BH. Differences in perioperative femoral and radial arterial blood pressure in neonates and infants undergoing cardiac surgery requiring cardiopulmonary bypass. J Pediatr (Rio J). 2018 Jan;94(1):76–81. | | | | | | | | | |  |  |  |  |  |

**Bibliography**:

1. Düzkaya, DS. et al. Chlorhexidine-Impregnated Dressings and Prevention of Catheter-Associated Bloodstream Infections in a Pediatric Intensive Care Unit. *Crit Care Nurse*. 2016 Dec;36(6):e1-e7
2. Gerçeker, GÖ. et al. Randomized controlled trial of care bundles with chlorhexidine dressing and advanced dressings to prevent catheter-related bloodstream infections in pediatric hematology-oncology patients. *Eur J Oncol Nurs*. 2017 Jun;28:14-20.
3. Levy, I. et al. Chlorhexidine-impregnated dressing for prevention of colonization of central venous catheters in infants and children: a randomized controlled study. *Pediatr Infect Dis J*. 2005 Aug;24(8):676-9.
4. Garland, JS. et al. A randomized trial comparing povidone-iodine to a chlorhexidine gluconate-impregnated dressing for prevention of central venous catheter infections in neonates. *Pediatrics*. 2001 Jun;107(6):1431-6.
5. Hatle,r C. et al. Evaluating central venous catheter care in a pediatric intensive care unit. *Am J Crit Care*. 2009 Nov;18(6):514-20
6. Onder, AM. et al. Controlling exit site infections: does it decrease the incidence of catheter-related bacteremia in children on chronic hemodialysis? *Hemodial Int*. 2009 Jan;13(1):11-8.
7. Ruiz, RR. et al. Efficacy of a chlorhexidine-gluconate impregnated patch for prevention of catheter-related infections in pediatric patients: systematic review and meta-analysis. *Bol Med Hosp Infant Mex* 2011;68(5):349-355.
8. Rijnders, BA. et al. Catheter-tip colonization as a surrogate end point in clinical studies on catheter-related bloodstream infection: how strong is the evidence? *Clin Infect Dis*. 2002 Nov 1;35(9):1053-8.

| **Chlorhexidine-impregnated dressings compared to standard dressings of CVC in children** | | | | | | | | | | | | | | | | | |
| --- | --- | --- | --- | --- | --- | --- | --- | --- | --- | --- | --- | --- | --- | --- | --- | --- | --- |
| Quality evaluation | | | | | | | | | Number of patients | | Effects | | | | | Quality of evidence | Importance |
| Author | Age | Population | Type of study | Study plan/evaluation | Inconsistencies | Imprecision | Risk of bias | Complexity | Study | Control | Rate study | Rate control | p | RR | CI 95% |  |  |
| **Incidence of bacteremic infections linked or associated with the cathete**r (ILC) | | | | | | | | | | | | | | | | | |
| Düzkaya | 1 month-18 years | PICU | RCT | Well | N | O ^1-2^ | N | N | 50 | 50 | 2,00% | 10,00% | NS (>0,05) |  |  | Low | Important |
| Gerceker | 2 month-18 years | Hemato-onco | RCT | Poor | N | O ^1^ | 0 | N | 14 | 13 | 3,9/1000 Days CVC | 4,4/1000 Days CVC | NS (>0,05) |  |  | Very low | No important |
| Hatler | 0-21 years | PICU | Observational | Poor | N | O ^1^ | O | N | 21 | 18 | 0 | 0 | NS (>0,05) |  |  | Very low | No important |
| Ruiz | 0-18 years | PICU, PCICU | MA* |  |  |  |  |  | 409 | 441 | 3,91% | 3,40% | 0,71 | 1,14 | [0,57- 2,28] | Moderate | Important |
| Onder |  | Haemodialysis | Retrospective |  |  |  |  |  |  |  | 4,2/1000 Days CVC | 5,5/1000 Days CVC | NS |  |  | Very low | No important |
|  | | | | | | | | | | | | | | | | | |
| **incidence of local infections linked to the catheter (ILC)** | | | | | | | | | | | | | | | | | |
| Düzkaya | 1 month-18 years | PICU | RCT | Well | N | O ^1-2^ | N | N | 50 | 50 | 2,00% | 4,00% | NS (>0,05) |  |  | Low | Important |
| Gerceker | 2 month-18 years | Hemato-onco | RCT | Poor ^3^ | N | O ^1^ | N | N | 14 | 13 | 0,00% | 14,40% | 0,22 |  |  | Low | Important |
| Onder |  | Haemodialysis | Retrospective |  |  |  |  |  |  |  | 1,1/1000 Days CVC | 0,2/1000 Days CVC | < 0,05 |  |  |  |  |
|  | | | | | | | | | | | | | | | | | |
| **CVC colonization rate** | | | | | | | | | | | | | | | | | |
| Düzkaya | 1 month-18 years | PICU | RCT | Well | N | O ^1-2^ | N | N | 50 | 50 | 2,00% | 8,00%4% | NS (>0,05) |  |  | Moderate | Important |
| Ruiz | 0-18 years | PICU, PCICU | MA* |  |  |  |  |  | 409 | 441 | 14,18% | 23,35% | 0,0007 | 0,6 | [0,45- 0,81] | Elevated | Crucial |
|  | | | | | | | | | | | | | | | | | |
| **Skin irritation** | | | | | | | | | | | | | | | | | |
| Gerceker | 2 month-18 years | Hemato-onco | RCT | Poor ^3^ | N | O ^1^ | N | N | 14 | 13 | 0,00% | 14,40% | 0,22 |  |  | Low | Important |
| Ruiz | 0-18 years | PICU, PCICU | MA* |  |  |  |  |  | 409 | 441 | 5,60% | 0,20% | 0,04 | 8,17 | [1,19- 5,6 | Elevated | Crucial |

**Légende**

O = yes N = no

NE = no study 1-effectifs réduits

NS = no significative 2-nbr of days CVC unspecified

NR = no specified 3- premature shutdown due to lack of equipment

RCT = Randomised Controlled Trial 4-very low duration

MA = Meta-analysis 5-no randomisation

PICU = pediatric intensive care unit * Levy 2005 PIDJ - Garland 2001 Pediatrics

PCICU pediatric cardiac intensive care unit

**Funnel and Forrest Plot from Ruiz’s meta-analysis ^7^**

| **quality improvement program in children to decrease the rate of catheter-related infections vs no paediatrics** | | | | |  |  |  |  |  |  |  |  |  |  |  |
| --- | --- | --- | --- | --- | --- | --- | --- | --- | --- | --- | --- | --- | --- | --- | --- |
|  |  |  |  |  |  |  |  |  |  |  |  |  |  |  |  |
| **Quality evaluation** | | | | | | | | | **Number of catheters** | | **Effects** | | **Quality** | **Importance** |  |
| **Number of study** | **Author** | **Type of study** | **Country** | **Plan experience / execution** | **Incoherence** | **Indirect data** | **Imprecision** | **Publication bias** | **bundle** | **control** | **IRR(CI95%)** | **DI (/1000j KT) with vs without** |  |  |  |
| Infection rate(DI CLABSI/1000 j KT) | | | | | | | | | | | | | | |  |
| 14 | Ista and al 2016 | Meta-analysis |  |  | I² = 0% |  |  |  |  |  | 0,58 (0,48-0,71) | 0 à 16,5 vs 2,6 à 31,1/1000 Days catheter | **MODERATE** |  | 79 PICU, retrospective study ; before/after;  no RCT |
|  | Biasucci and al 2017 | observational |  |  |  |  |  |  |  |  |  | 1,5 vs 15/1000 Days catheter | **LOW** |  |  |

| **First author** | **Journal** | **Date** | **Type** | **Population** | **Unit** | **Number** | **Primary outcome** | **CRBSI** | **Colonization** | **Thrombosis** | **Mechanical complication** | **Remark** | **PMID** |
| --- | --- | --- | --- | --- | --- | --- | --- | --- | --- | --- | --- | --- | --- |
| **Randomized Controlled trials** | |  |  |  |  |  |  |  |  |  |  |  |  |
| Parienti | NEJM | 2015 | RCT | adult | ICU | 3027 | Composite: CRBI + Thrombosis hazard ratio, 1.3; 95% CI, 0.8 to 2.1; P=0.30 | 11.8 vs. 13.8 |  | 0.9 vs. 1.42.4 [1.0-5.6] 0.04 | Pneumothorax : 0.5% | Major complication and use of ultrasound 1185 0.14 [0.03-0.71] | 26398070 |
| Nakae | Artif Organs | 2010 | RCT | adult | ICU | 56 |  | No site effect: 0 vs. 4 | Not affected by site | NR | NR | No time effect, no systematic change | 20447037 |
| Parienti | JAMA | 2008 | RCT | adult | ICU | 750 |  | incidence per 1000 catheter-days, 1.5; 95% CI, 0.1-6.4) and in 5 of 313 patients (1.6%) with jugular catheters (incidence per 1000 catheter-days, 2.3; 95% CI, 0.3-7.7). This difference was not significant by Poisson regression (P=.42) | Incidence of 40.8 vs 35.7 per 1000 catheter-days; hazard ratio [HR], 0.85; 95% confidence interval [CI], 0.62-1.16; P = .31 | 0.5% vs 0.5% | 3.6 vs. 1.1% | Jugular better in high BMI | 18505951 |
| Male | Thromb Haemost | 2005 | RCT ? | Children | General paediatric | 158 | Thrombosis |  |  | Phlebo: 32 vs 8% |  | No time effect |  |
| Durbec | CCM | 1997 | RCT | adult | ICU | 61 | Thrombosis |  |  | 16% vs. 40%Clinical: 3%vs 6.6% |  |  |  |
| Trottier | CCM | 1995 | RCT |  |  |  | Thrombosis |  |  | 0 vs. 25% |  |  |  |
| **Meta-analysis** |  |  |  |  |  |  |  |  |  |  |  |  |  |
|  |  |  |  |  |  |  |  |  |  |  |  |  |  |
| Arvaniti | CCM | 2017 | 20 studies |  |  | 18554 catheters |  | relative risk, 0.55 [95% CI, 0.34-0.89]; I = 61% - No difference after exclusion of observational studies |  |  |  |  |  |
| Timsit | AJRCCM | 2013 | 2 studies | adult | ICU | 2527 catheters |  | [HR], 0.63 [0.25-1.63]; P = 0.34 | internal jugular 11.6 vs. femoral 12.9 per 1,000 catheter-days; HR, 0.80 [0.25-1.63]; P = 0.15 | NR |  | No benefit before day 5 | 24127770 |
| Marik | CCM | 2012 | 2 RCT & 8 Observational |  |  | 3230 catheters |  | No difference in RCT & risk ratio 1.90; 95% confidence interval 1.21-2.97, p=.005, I²=35% |  | RCT: No difference |  |  |  |
|  |  |  |  |  |  |  |  |  |  |  |  |  |  |
|  |  |  |  |  |  |  |  |  |  |  |  |  |  |
| Ge | Cochrane | 2012 | 4 studies |  |  | 736 catheters |  | RR: 0.58 (0.14 to 2.4) | RR 1.04 (0.8 to 1.36) (fvsj) | Symptomatic RR: 0.99 (0.14 to 6.98) | Total: RR 0.51 (0.29 to 0.88) |  | 22419292 |
|  |  |  |  |  |  |  |  |  | Highest BMIRR 1.69 (1.08 to 2.65) (fvsj) | Thrombosis RR 0.46 (0.21 to 1.01) | Major RR 0.33 (0.03 to 3.16) |  |  |

| **CHG Alc vs PVI Alc** | | | | | | | | | | | | |
| --- | --- | --- | --- | --- | --- | --- | --- | --- | --- | --- | --- | --- |
|  | | | | | | | **Number of patients** | | **Effects** | | **Quality** | **Importance** |
| **study** | **type of study** | **risk of bias** | **inconsistences** | **indirect data** | **imprecision** | **publication bias** | **CHG** | **PVI** | **statistics** | **WMD** |  |  |
| **Catheter related infections** | | | | | | | | | | | | |
| Mimoz 2015 | RCT | Possible | No | No | No | Possible | 0,28/1000 j | 1,77/1000 j | HR 0,15 (0,05-0,41) 0,0002 |  | **HIGH** | 9 (out of 10) |
| Pages 2016 | cohort QE | Possible | No | No | No | No | 2/1000 J | 4,5/1000 j | A HR 0,51 (0,29-8-0,96) 0,037 |  | **MODERATE** | 8 out of 10 |
|  |  |  |  |  |  |  |  |  |  |  |  |  |
| **secondary cutaneous effects** | |  |  |  |  |  |  |  |  |  |  |  |
| Mimoz | RCT | Possible | No | No | Possible | Possible | 27/1181 | 7/1168 | 0,0017 |  | **MODERATE** |  |

| **Central vein and arterial catheters** | | |  |  |  |  |  |  |  |  |  |  |  |
| --- | --- | --- | --- | --- | --- | --- | --- | --- | --- | --- | --- | --- | --- |
| **Disinfection after detersion (4 steps) of without detersion (1 step)** | | | | | | | | | | | | | |
|  |  |  |  |  |  |  |  |  |  |  |  |  |  |
|  | | | | | | | | **Number of catheters** | | **Effects** | | **quality** | **Importance** |
| **Study** | **type of study** | **risk of bias** | **inconsistencies** | **indirect calculations** | **imprecision** | **publication bias** | **disinfection** | **4 steps** | **1 step** | **Stat** |  |  |  |
| ***Catheter colonization*** | |  |  |  |  |  |  |  |  |  |  |  |  |
| Mimoz 2015 | RCT | low | No | No | No | Not detected | PVI-alc | 1286 | 1326 |  |  |  |  |
|  |  |  |  |  |  |  | Chlorexidine alc 2% | 1270 | 1277 |  |  |  |  |
|  |  |  |  |  |  |  | total | 2556 | 2603 | p= 0,8887 |  | **high** | **high** |
|  | | | | | | | | **Number of catheters** | | **Effects** | | **Quality** | **Importance** |
| **Study** | **type of study** | **risk of bias** | **inconsistencies** | **indirect calculations** | **imprecision** | **publication bias** | desinfection | **4 steps** | **1 step** | **Stat** |  |  |  |
| ***Catheter related infection*** | | | | | | | | | | | | | |
| Mimoz 2015 | RCT | average | No | No | No | Not detected | PVI-alc | 1286 | 1326 |  |  |  |  |
|  |  |  |  |  |  |  | Chlorexidine alc 2% | 1270 | 1277 |  |  |  |  |
|  |  |  |  |  |  |  | total | 2556 | 2603 | p= 0,174 |  | **high** | **high** |
|  | | | | | | | | **Number of catheters** | | **Effects** | | **Quality** | **Importance** |
| **Study** | **type of study** | **risk of bias** | **inconsistencies** | **indirect calculations** | **imprecision** | **publication bias** | disinfection | **4 steps** | **1 step** | **Stat** |  |  |  |
| ***CRBSI*** |  |  |  |  |  |  |  |  |  |  |  |  |  |
| Mimoz 2015 | RCT | average | No | No | No | Not detected | PVI-alc | 1286 | 1326 |  |  |  |  |
|  |  |  |  |  |  |  | Chlorexidine alc 2% | 1270 | 1277 |  |  |  |  |
|  |  |  |  |  |  |  | total | 2556 | 2603 | p= 0,1645 |  | **high** | **high** |
| ***Cutaneous toxicity*** | |  |  |  |  |  |  |  |  |  |  |  |  |
| Mimoz 2015 | RCT | Yes | No | No | Possible | Not detected | total | 2556 | 2603 |  |  | **Average** |  |

|  |  |  |  | **quality assessment** | **Number of patients** | | **Effect** | **Quality** | **Importance** | **Commentary** |
| --- | --- | --- | --- | --- | --- | --- | --- | --- | --- | --- |
| **Reference** | **Type of study** | **Population** | **catheter type** | **risk of bias** | **Intervention** | **control** | **RR (CI95%)** |  |  |  |
|  |  |  |  |  |  |  |  |  |  |  |
| Chlorhexidine silver sulfadiazine (CRBSI) | | | | | | | | | |  |
| **Cochrane 2016** (19 study) | Meta-analysis-RCT | Adults | CVC | low | **2403** | **2483** | **0.73 (0.57-0.94)** | high | High | Heterogeneity (Chi2 0.20) I2=21% with test Z =0.016 |
| Chlorhexidine silver sulfadiazine (CRBSI/1000 catheter days) | | | | | | | | | |  |
| **Cochrane 2016** (6 study) | Meta-analysis-RCT | Adults | CVC | low | **759** | **820** | **1.20 (0.70-2.06)** | high | High | Heterogeneity (Chi2 0.29) I2=19% with test Z =0.51 |
| **Lorente AJIC 2014** | Observational, monocenter | Adults | CVC | High | **245** | **391** | **0.02 (infini-0.276)** | Low | High | Incidence density of CRBSI 0 vs 5.04 CRBSI per 1000 days/catheters |
|  | Chlorhexidine silver sulfadiazine (infection locale) | | | | | | | | |  |
| **Cohrane 2016** (7 study) | Meta-analysis-RCT | Adults | CVC | High | **739** | **808** | **0.93 (0.72-1.21)** | Average | High | Heterogeneity (Chi2 0.86) I2=0% with test Z =0.59 |
|  |  |  |  |  |  |  |  |  |  |  |
| Minocyclin-Rifampin (CRBSI) | | | | | | | | | |  |
| **Cochrane 2016** (4 study) | Meta-analysis-RCT | Adults | CVC | low | **685** | **650** | **0.26 (0.13-0.49)** | high | High | Heterogeneity (Chi2 0.35) I2=9% with test Z =0.00003 |
| **Weber JBCR 2012** | historical cohort | Paediatric burn | CVC | High | **75** | **66** | ND | Low | Low | incomplete abstract |
| **Youssif AJIC 2016** | Observational monocenter | Adults | PiccLine | High | **65** | **94** | ND | Low | Low | 1.7 CRBSI rate in the control group vs 0 CRBSI in the interventional group (p=0.066) |
| **Gilbert Lancet 2016** | RCT | Children | CVC | low | **486** | **502** | **0.43 (0.20-0.96)** | high | High | This study includes also a heparin group. CRBSI was a secondary objective. Risk reduction of 57% in favour of antibiotic impregnated catheters (18 CRBSI vs 7 CRBSI) |
| Minocyclin-Rifampin (CRBSI/1000 catheter days) | | | | | | | | | |  |
| **Cochrane 2016** (3 study) | Meta-analysis-RCT | Adults | CVC | low | **483** | **462** | **0.39 (0.11-1.12)** |  |  |  |
|  | Minocyclin-Rifampin (local infection) | | | | | | | | |  |
| **Leon 2004** | RCT | Adults | CVC | low | **187** | **180** | **1.16 (0.36-3.72)** | moderated | High |  |
|  |  |  |  |  |  |  |  |  |  |  |
|  |  |  |  |  |  |  |  |  |  |  |
| **Ref** | **Type of study** | **Population** | **catheter type** | **risk of bias** | **Intervention** | **control** | **RR (CI95%)** |  |  |  |
| **Cochrane 2014** (2 study) | Meta-analysis RCT | Children |  | low | **144** | **143** | **0.34 (0.01-7.68)** | moderated |  | Heterogeneity (Chi2 0.03) I2=80% with test Z =0.49 |

|  |  | **Y: Yes; N: No; S: serious; VS: Very serious; RCT randomized controlled trial; MA: meta-analysis** | | | | | | | | | | | | | | | | | | |
| --- | --- | --- | --- | --- | --- | --- | --- | --- | --- | --- | --- | --- | --- | --- | --- | --- | --- | --- | --- | --- |
|  |  | **Antiseptic impregnated catheters** | | | | | | | | | |  | | **Effect** | |  |  |  | **Quality** | **Importance** |
| ref |  | **Number of studies** | **Type of studies** | **Countries** | **Plan/nature of experiments** | **objective** | **risk of bias** | **Consistencies** | **indirect data** | **inconsistencies** | **publication bias** | **number of patients** |  | **Antiseptic dressings** | **control arm** | **Relative effect OR(95% CI)** | **Absolute effect (95% CI)** | **Mean difference** |  |  |
|  |  |  |  |  |  |  |  |  |  |  |  |  |  |  |  |  |  |  |  |  |
|  |  | colonisation | | | | | | | | | | | | | | | | | | |
| 1 | Roberts and al. 1998 | 1 | RCT | Australia | To assess the effect of chlorhexidine gluconate–impregnated sponge to be placed over the CV catheter insertion site on the rate of catheter colonization in adult ICU patients | colonisation | S |  | N | VS | not reported | 33 |  | 0 of 17 | 1 of 16 | NS |  |  | VERY LOW |  |
| 2 | Levy et and. 2005 | 1 | RCT | Israel | To assess the effect of chlorhexidine gluconate–impregnated sponge to be placed over the CV catheter insertion site on the rate of catheter colonization in paediatric ICU patients | colonisation | S |  | N | S | not reported | 145 patients- 1 paediatric ICU | | 14.8% of patients | 29% of patients | 0.62 (0.37-1.02) | |  | LOW |  |
| 3 | Timsit and al. 2009 | 1 | RCT | France | To assess the effect of chlorhexidine gluconate–impregnated sponge to be placed over the CV or arterial catheter insertion site on the rate of catheter colonization in adult ICU patients | colonisation | S |  | N | N | S- Ethicon Inc donated the Biopatch dressings used in the study. | 1636-7 adult ICUs | | 6.3 per 1000 catheter-days | 15.8 per 1000 catheter-days | 0.36 (0.28-0.46) | |  | HIGH |  |
| 4 | Timsit and al. 2012 | 1 | RCT | France | To assess the effect of chlorhexidine impregnated gel dressing to be placed over the CV or arterial catheter insertion site on the rate of catheter colonisation in adult ICU patients | colonisation | S |  | N | N | VS - An unrestricted research grant was obtained by University Grenoble 1/Albert Michallon University Hospital from 3M Company | 1879-12 adult ICUs | | 4.3 per 1000 catheter-days | 10.9 per 1000 catheter-days | 0.41 (0.31–0.56) | |  | HIGH |  |
| 5 | Arvaniti and al. 2012 | 1 | RCT | Greece | To assess the effect of chlorhexidine gluconate–impregnated sponge to be placed over the CV catheter insertion site on the rate of catheter colonization in adult ICU patients | colonisation | S |  | N | S | not reported | 465-five adult ICUs | | 19.9 per 1000 catheter-days | 20.9 per 1000 catheter-days | 1.21 (0.56–2.61) | |  | MODERATE |  |
| 6 | Ho and al. 2006 | 5 | MA | Australia | Contents neonat and out of ICU-2/5 study in ICU | colonisation |  | include neonates and out of ICU | | heterogeneous and precise | | |  | 1174 patients | 1272 patients | 0.47 | 0.34-0.65 |  |  |  |
| 7 | Safdar and al. 2014 | 7 | MA | USA | contents neonat and out of ICU-include timsit x2-5/ study in ICU | colonisation |  | include neonates and out of ICU | | heterogeneous and precise | | |  | 5281 KT | 5200 KT | 0.52 | 0.43-0.64 |  |  |  |
| 8 | Ullman and al. 2016 | 6 | MA | Australia | 4 study with 3 ICU et 1 hematoonco | colonisation |  | include neonates and out of ICU | | heterogeneous and precise | | |  | 2591 patients | 1840 patients | 0.58 | 0.47-0.73 |  |  |  |
| ref |  | **Number of studies** | **Type of studies** | **Countries** | **Plan/nature of experiments** | **objective** | **risk of bias** | **Consistencies** | **indirect data** | **inconsistencies** | **publication bias** | **number of patients** |  | **Antiseptic dressings** | **control arm** | **Relative effect OR(95% CI)** | **Absolute effect (95% CI)** | **Mean difference** |  |  |
|  |  | infection |  |  |  |  |  |  |  |  |  |  |  |  |  |  |  |  |  |  |
| 2 | Levy and al. 2005 | 1 | RCT | Israel | To assess the effect of chlorhexidine gluconate–impregnated sponge to be placed over the CV catheter insertion site on the rate of CLABSI in paediatric ICU patients | CLABSI | S |  | N | S | not reported | 145 patients- 1 paediatric ICU | | 5.4% of patients | 4.2% of patients | NA | NA |  | LOW |  |
| 3 | Timsit and al. 2009 | 1 | RCT | France | To assess the effect of chlorhexidine gluconate–impregnated sponge to be placed over the CV or arterial catheter insertion site on the rate of major catheter-related infection (catheter-related clinical sepsis or CRBSI) in adult ICU patients | MCRI | S |  | N | N | S- Ethicon Inc donated the Biopatch dressings used in the study. | 1636-7 adult ICUs | | 0.60 per 1000 catheter-days | 1.40 per 1000 catheter-days | 0.39 (0.17-0.93) | 1 infection prevented for 117 catheters (86-1020) | | HIGH |  |
| 3 | Timsit and al. 2009 | 1 | RCT | France | To assess the effect of chlorhexidine gluconate–impregnated sponge to be placed over the CV or arterial catheter insertion site on the rate of CRBSI in adult ICU patients | CRBSI | S |  | N | N | S- Ethicon Inc donated the Biopatch dressings used in the study. | 1636-7 adult ICUs | | 0.4 per 1000 catheter-days | 1.3 per 1000 catheter-days | 0.24 (0.09-0.65) | |  | HIGH |  |
| 4 | Timsit and al. 2012 | 1 | RCT | France | To assess the effect of chlorhexidine impregnated gel dressing to be placed over the CV or arterial catheter insertion site on the rate of major catheter-related infection (catheter-related clinical sepsis or CRBSI) in adult ICU patients | MCRI | S |  | N | N | VS - An unrestricted research grant was obtained by University Grenoble 1/Albert Michallon University Hospital from 3M Company | 1879-12 adult ICUs | | 0.7 per 1000 catheter-days | 2.1 per 1000 catheter-days | 0.328 (0.17–0.62) | 1 infection prevented for 71 catheters (57-125) | | HIGH |  |
| 4 | Timsit and al. 2012 | 1 | RCT | France | To assess the effect of chlorhexidine impregnated gel dressing to be placed over the CV or arterial catheter insertion site on the rate of CRBSI in adult ICU patients | CRBSI | S |  | N | N | VS- An unrestricted research grant was obtained by University Grenoble 1/Albert Michallon University Hospital from 3M Company | 1879-12 adult ICUs | | 0.5 per 1000 catheter-days | 1.3 per 1000 catheter-days | 0.40 (0.19–0.87) | |  | HIGH |  |
| 5 | Arvaniti and al. 2012 | 1 | RCT | Grèce | To assess the effect of chlorhexidine gluconate–impregnated sponge to be placed over the CV catheter insertion site on the rate of CRBSI in adult ICU patients | CRBSI | S |  | N | N | not reported | 465-five adult ICUs | | 2.84 per 1000 catheter-days | 1.4 per 1000 catheter-days | 1.65 (0.27–10.01) | |  | MODERATE |  |
| 5 | Arvaniti and al. 2012 | 1 | RCT | Grèce | To assess the effect of chlorhexidine gluconate–impregnated sponge to be placed over the CV catheter insertion site on the rate of catheter related infection in adult ICU patients | CRI | S |  | N | N | not reported | 465-five adult ICUs | | 5.69 per 1000 catheter-days | 7.83 per 1000 catheter-days | 0.65 (0.23–1.85) | |  | MODERATE |  |
| 6 | Ho and al. 2006 | 5 | MA | Australia |  | CRBSI |  | include neonates and out of ICU | | heterogeneous and unspecified | | |  | 1149 patients | 1247 patients | 0.61 | 0.30-1.26 |  |  |  |
| 7 | Safdar and al. 2014 | 9 | MA | USA |  | CRBSI |  | include neonates and out of ICU | | heterogeneous and unspecified | | |  | 5281 KT | 5200 KT | 0.6 | 0.41-0.88 |  |  |  |
| 8 | Ullman and al. 2016 | 4 | MA | Australia |  | CRBSI |  | include neonates and out of ICU | | detailed and homogeneous | | |  | 2486 patients | 1735 patients | 0.51 | 0.33-0.78 |  |  |  |
|  |  |  |  |  |  |  |  |  |  |  |  |  |  |  |  |  |  |  |  |  |
| ref |  | **Number of studies** | **Type of studies** | **Countries** | **Plan/nature of experiments** | **objective** | **risk of bias** | **Consistencies** | **indirect data** | **inconsistencies** | **publication bias** | **number of patients** |  | **Antiseptic dressings** | **control arm** | **Relative effect OR(95% CI)** | **Absolute effect (95% CI)** | **Mean difference** |  |  |
|  |  | Skin tolerance | | | | | | | | | | | | | | | | | | |
| 2 | Levy and al. 2005 | 1 | RCT | Israel | To assess the effect of chlorhexidine gluconate–impregnated sponge to be placed over the CV catheter insertion site on the rate of skin local redness in paediatric ICU patients | | | | N |  | not reported | 145 patients- 1 paediatric ICU | | 4/75 patients | 1/71 patients |  |  |  | LOW |  |
| 3 | Timsit and al. 2009 | 1 | RCT | France | To asess the effect of chlorhexidine gluconate–impregnated sponge to be placed over the CV or arterial catheter insertion site on the rate of rate of abnormal scores according to the International Contact Dermatitis Research Group system | | | | N |  | S- Ethicon Inc donated the Biopatch dressings used in the study. | 1636-7 adult ICUs | | 100/6720 (1.49%) | 63/5875 (1.07%) | p=0.02 |  |  | HIGH |  |
| 4 | Timsit and al. 2012 | 1 | RCT | France | To assess the effect of chlorhexidine impregnated gel dressing to be placed over the CV or arterial catheter insertion site on the rate of rate of abnormal scores according to the International Contact Dermatitis Research Group system | | | | N |  | VS- An unrestricted research grant was obtained by University Grenoble 1/Albert Michallon University Hospital from 3M Company | | | 2.3% | 1% | P < 0.0001 |  |  | HIGH |  |
|  |  |  |  |  |  |  |  |  |  |  |  |  |  |  |  |  |  |  |  |  |
| ref |  | **Number of studies** | **Type of studies** | **Countries** | **Plan/nature of experiments** | **objective** | **risk of bias** | **Consistencies** | **indirect data** | **inconsistencies** | **publication bias** | **number of patients** |  | **Antiseptic dressings** | **control arm** | **Relative effect OR(95% CI)** | **Absolute effect (95% CI)** | **Mean difference** |  |  |
|  |  | Costs | | | | | | | | | | | | | | | | | | |
| 11 | Crawford and al. 2004 | 1 | Medico-economic data on public health data | | | |  |  | N | N | not reported | |  | economy 327 to 964 USD per patient | |  |  |  |  |  |
| 12 | Ye and al. 2011 | 1 | Medico-economic data on public health data public | | To assess the effect of chlorhexidine gluconate–impregnated sponge to be placed over the CV or arterial catheter insertion site on the expected cost by catheter (obtained by multiplying the overall cost of the strategy by the probability of CRBSI in ICU patients | | | | N | N | not reported | |  | Economy 290 USD per catheter | |  |  |  |  |  |
| 13 | Schwebel and al. 2012 | 1 | RCT- Ancillary study de Timsit and al. 2009 | France | To assess the effect of chlorhexidine gluconate–impregnated sponge to be placed over the CV or arterial catheter insertion site on the expected cost by catheter (obtained by multiplying the overall cost of the strategy by the probability of major catheter-related infection (catheter-related clinical sepsis or CRBSI)) in adult ICU patients | | | | N | N | S- Ethicon Inc donated the Biopatch dressings used in the study. | 1636-7 adult ICUs | | economy 133 to 197 USD per catheter | |  |  |  |  |  |
| 14 | Maunoury and al. 2015 | 1 | RCT-ancillary of Timsit and al. 2012 | | To assess the effect of chlorhexidine impregnated gel dressing to be placed over the CV or arterial catheter insertion site on the expected cost by catheter (obtained by multiplying the overall cost of the strategy by the probability of major catheter-related infection (catheter-related clinical sepsis or CRBSI)) in adult ICU patients | | | | N | N | VS- An unrestricted research grant was obtained by University Grenoble 1/Albert Michallon University Hospital from 3M Company | | | economy 141 euros (CI -975 to 1258 euros) | | |  |  |  |  |
| 15 | Thokala and al. 2016 | 1 | Médico-economic on public health data | | To assess the effect of chlorhexidine impregnated gel dressing to be placed over the CV insertion site on the expected cost by patient (obtained by multiplying the overall cost of the strategy by the probability of CRBSI) in ICU patients | | | | N | N | N |  |  | economy 77 pounds per patient | |  |  |  |  |  |

| **Short (2 - 5 days) vs long (5-15 days) time intervals between dressing changes** | | | | | |  |  |  |  |  |  |  |  |
| --- | --- | --- | --- | --- | --- | --- | --- | --- | --- | --- | --- | --- | --- |
| **Evaluation quality** | | | | | | | **Number of patients** | | **Effects** | | **Quality** | **Importance** | **Comments** |
| **Ref** | **Type of study** | **risk of bias** | **inconsistencies** | **indirect data** | **imprecision** | **publication bias** | **intervention** | **control** | **Relative risk (CI95%)** | **absolute Risk** |  |  |  |
| Catheter-related bloodstream infection | | | | | | | | | | | | |  |
| Benhamou 2002 | Monocenter RCT | Serious | Serious | Serious | Serious | Undetected | 56 | 56 |  | 1.8 vs 1.8, p=ns | Low | Important | Defined as the same species isolated from skin insertion site culture and blood culture |
| Timsit 2009 | multicenter, 2x2 factorial, RCT | Not serious | Not serious | No | Not serious | Undetected | 825 | 828 | 1,26 (0,47-3,34, p=0,65 |  | Good | Important | Defined as a combination of ≥ 1 positive peripheral blood cultures sampled immediately before or within 48 h after catheter removal; a quantitative catheter-tip culture testing positive for the same micro-organism or a differential time to positivity of blood cultures ≥ 2 h; no other infectious focus explaining the positive blood culture result |
|  |  |  |  |  |  |  |  |  |  |  |  |  |  |
| Catheter-related infection with bacteraemia or no | | | | | | | | | | | | |  |
| Timsit 2009 | multicentre RCT | Not serious | Not serious | No | Not serious | Undetected | 825 | 828 | 1,16 (0,50-2,69), p=0,74 |  | Good | Important | CRBSI or Catheter-related clinical sepsis without bloodstream infection defined as fever ≥ 38.5°C or ≤ 36.5°C; catheter tip culture yielding ≥ 10³ CFU/ml; pus at the insertion site or resolution of clinical sepsis after catheter removal; absence of any other infectious focus |
|  |  |  |  |  |  |  |  |  |  |  |  |  |  |
| Local infections | | | | | | | | | | | | |  |
| Engervall 1995 | monocentre RCT | Serious | Serious | Serious | Serious | Undetected | 16 | 16 |  | 11/14 (78.6%) vs 2/9 (22.2%) catheters in culture, p=0.02 | very low | not important | > 15 CFU at catheter tip culture |
| Benhamou 2002 | monocentre RCT | Serious | Serious | Serious | Serious | Undetected | 56 | 56 |  | 15/56 (27%) vs 13/56 (23%) cathéters in culture, p=ns | low | not important | Positive culture of skin insertion site |
| Timsit 2009 | multicentre, 2x2 factorial, RCT | Not serious | Not serious | No | No | Undetected | 825 | 828 | 0,99 (0,77-1,28), p=0,95 |  | high | not important | Catheter colonisation defined as catheter tip culture yielding ≥ 10³ CFU/ml |
|  |  |  |  |  |  |  |  |  |  |  |  |  |  |
| Skin lesions | | | | | | | | | | | | |  |
| Benhamou 2002 | monocentre RCT | Serious | might not exist | Serious | Serious | Undetected | 56 | 56 |  | 37%/48%/11%/2%/2% VS 18%/39%/34%/7%/2%, p=0,001 | good | important | 0: healthy skin; 1: slightly inflamed skin; 2: minor cutaneous lesions, dressing difficult to remove; 3: lesions reaching periphery of the dressing; grade 4: cutaneous lesions to such and extent that the usual dressing could no longer be used |
| Vokurka 2009 | multicentre RCT | Serious | might not exist | Serious | Serious | Undetected | 39 | 42 |  | 67%/15%/18%/0%/0% vs 60%/26%/12%/2%/0%, p=ns | good | important | 0: healthy skin, 1: erythema, 2: erythema with itching or dry desquamation, 3: moist desquamation, exfoliation, 4: deep ulceration, necrosis |
| Timsit 2009 | multicentre, 2x2 factorial, RCT | Not serious | might not exist | No | Not serious | Undetected | 825 | 828 |  | 90%/9%/0%/1%/0% vs 91%/8%/0%/1%/0%, p=ns | good | important | 0: normal skin; 1: mild redness only; 2: red and slightly thickened skin; 3: intense redness and swelling with coalesced large blisters or spreading reaction |
|  |  |  |  |  |  |  |  |  |  |  |  |  |  |
| Mortality from all causes | | | | | | | | | | | | |  |
| Engervall 1995 | monocenter RCT | Not serious | No | Serious | Serious | Undetected | 16 | 16 |  | 37.5% vs 37.5%, p=ns | low | important |  |
| Benhamou 2002 | monocenter RCT | Not serious | No | Serious | Serious | Undetected | 56 | 56 |  | 0% vs 3.6%, p =ns | low | important |  |
| Timsit 2009 | multicenter, 2x2 factorial, RCT | Not serious | No | No | Not serious | Undetected | 825 | 828 |  | 40,5% vs 38,4%, p=ns | good | important |  |
| Engervall 1995 | Adult, neoplasia or severe aplasia - CVC- Change dressing : 1/week vs 2/week | | | | | |  |  |  |  |  |  |  |
| Benhamou 2002 | Children (median 6, range 1-22 years) with neoplasia requiring graft MO and chemotherapy IV -CVC - Change dressing : 15daysvs 4days | | | | | | | | | |  |  |  |
| Vokurka 2009 | Adult, leukemia myeloid requesting chemotherapy IV - CVC no tunnelised - Change dressing : 1/week vs 2/week | | | | | | | | |  |  |  |  |
| Timsit 2009 | Adult, ICU - CVC and CA - change dressing : 7daysvs 3d | | | |  |  |  |  |  |  |  |  |  |

| **Ultrasound guidance catheter insertion jugular vein** | | | | | | | | | | | | | | | | | | |
| --- | --- | --- | --- | --- | --- | --- | --- | --- | --- | --- | --- | --- | --- | --- | --- | --- | --- | --- |
| **study** | **Type of study** | **Population** | **country** | **Nb studies screened Meta -analysis)** | **Nb study used (Meta -analysis)** | **nb studies** | **nb patients** | **Nb control group** | **number group ultrasons** | **nb events control group** | **Number events US group** | **RR** | **risk of Bias** | **inconsistencies** | **Indirect data** | **Imprecisions** | **Quality overall** | **Importance** |
| Complications ultrasound vs landmark |  |  |  |  |  |  |  |  |  |  |  |  |  |  |  |  |  |  |
| Brass and al Cochrane meta-analysis (direct puncture)(adults) | Meta-analysis | General | France | 439 | 35 | 14 | 2406 | 1212 | 1194 | 161 | 48 | 0,29(0,17-0,52) p<0,0001 | yes | no | yes | no | high | high |
| Hind | Meta-analysis | General | UK | 1158 |  | 18 | 579 | 312 | 296 | not reported | not reported | 0,43(0,22-0,87 )p=0,02 | no | yes | yes | yes | moderate | moderate |
| Shime | Meta-analysis | paediatric | Singapore |  |  | 9 | 579 | not reported | not reported | not reported | not reported | not reported | no | yes | yes | yes | low | low |
| Mallory | Randomized prospective | ICU | US |  |  |  | 29 | not reported | not reported | not reported | not reported | not reported | no | no | no | yes | low | low |
| Slama | Randomized prospective | ICU | France |  |  |  | 79 | 42 | 37 | 5 | 5 | 1 | no | no | no | no | moderate | moderate |
| Teichgraber | randomized prospective | ICU | US |  |  |  | 100 | 50 | 50 | 28 | 6 | 0,54(0,41-0,71) p<0,0001 | no | no | no | yes | moderate | moderate |
| Karakitsos | randomized prospective | ICU | Greece |  |  |  | 700 | 450 | 450 | 105 | 7 | 0,066(0,03-0,14) p<0,0001 | no | no | no | no | high | high |
| Milling | randomized prospective | ICU | US |  |  |  | 129 | 69 | 60 | 8 | 2 | 0,15(0,03-0,68) p=0,014 | no | no | no | no | moderate | moderate |
| Palepu | randomized prospective | ICU | India |  |  |  | 399 | 194 | 205 | 19 | 10 | 0,54(0,27-1,12) p=0,099 | no | no | no | no | high | high |
| Agarwal | randomized prospective | ICU | India |  |  |  | 80 | 40 | 40 | 5 | 0 | 0,09(0,005-1,59) p=0,1 | no | no | no | yes | low | low |
| Airapetian | randomized prospective | ICU | France |  |  |  | 37 | 38 | 36 | 7 | 0 | 0,09(0,005-1,45) p=0,09 | no | no | no | yes | moderate | moderate |
| Rando | randomized prospective | ICU and Surgery | Uruguay |  |  |  | 257 | 134 | 123 | 20 | 10 | 0,54(0,26-1,11) p=0,097 | no | no | no | no | moderate | moderate |
| Arterial puncture ultrasound vs landmark |  |  |  |  |  |  |  |  |  |  |  |  |  |  |  |  |  |  |
| Brass and al Cochrane meta-analysis | Meta-analysis | General | France |  |  | 18 | 3920 |  |  |  |  | 0,26 (0,18-0,37) |  |  |  |  |  |  |
| Shime | Meta-analysis | paediatric | Singapore |  |  | 9 |  | Not found | Not found | Not found | Not found | 0,31(0,09-1,08)p=0,07 |  |  |  |  |  |  |
| Slama | randomized prospective | ICU | France |  |  |  | 79 | 42 | 37 | 5 | 5 | 1 |  |  |  |  |  |  |
| Teichgraber | randomized prospective | ICU | US |  |  |  | 100 | 50 | 50 | 12 | 0 | 0,04(0,002-0,65)p=0,02 |  |  |  |  |  |  |
| Karakitsos | randomized prospective | ICU | Greece |  |  |  | 700 | 450 | 450 | 48 | 5 | 0,19(0,07-0,46)p=0,0003 |  |  |  |  |  |  |
| Agarwal | randomized prospective | ICU | India |  |  |  | 80 | 40 | 40 | 4 | 0 | 0,11(0,0006-1,99)p=0,13 |  |  |  |  |  |  |
| Airapetian | randomized prospective | ICU | France |  |  |  | 74 | 38 | 36 | 5 | 0 | 0,098(0,005-1,71)p=0,11 |  |  |  |  |  |  |
| Milling | randomized prospective | ICU | US |  |  |  | 129 | 69 | 60 | 8 | 2 | 0,15(0,03-0,68)p=0,014 |  |  |  |  |  |  |
| Haematoma ultrasound vs landmark |  |  |  |  |  |  |  |  |  |  |  |  |  |  |  |  |  |  |
| Brass and al Cochrane meta analysis | Meta-analysis | General | France |  |  | 11 | 3047 |  |  |  |  | 0,23(0,12-0,44) |  |  |  |  |  |  |
| Teichgraber | randomized prospective | ICU | US |  |  |  | 100 | 50 | 50 | 10 | 2 | 0,2(0,02-1,65)p=0,14 |  |  |  |  |  |  |
| Karakitsos | randomized prospective | ICU | Greece |  |  |  | 700 | 450 | 450 | 38 | 2 | 0,097(0,23-0,4)p=0,0012 |  |  |  |  |  |  |
| Airapetian | randomized prospective | ICU | France |  |  |  | 74 | 38 | 36 | 6 | 0 | 0,08(0,004-1,142)p=0,08 |  |  |  |  |  |  |
| Milling | randomized prospective | ICU | US |  |  |  | 129 | 69 | 60 | 0 | 0 | 1 |  |  |  |  |  |  |

| **Ultrasound guidance catheter insertion femoral vein** | | | | |  |  |  |  |  |  |  |  |  |  |  |  |  |  |
| --- | --- | --- | --- | --- | --- | --- | --- | --- | --- | --- | --- | --- | --- | --- | --- | --- | --- | --- |
| **study** | **Type of study** | **Population** | **country** | **Nb studies screened Meta-analysis)** | **Nb study used (Meta-analysis)** | **nb studies** | **nb patients** | **Nb contro lgroup** | **number group ultrasons** | **nb events control group** | **number events US group** | **RR** | **risk of Bias** | **inconsistencies** | **Indirect data** | **Imprecisions** | **Quality overall** | **Importance** |
| Complications ultrasound vs landmark | | | | | | | |  |  |  |  |  |  |  |  |  |  |  |
| Brass | meta-analysis | miscelaneous | France | 210 | 4 |  |  |  |  |  |  |  | non | non | yes | yes | Very low | Low |
| Hilty | prospective randomised | ACR | US | 40 |  |  | 40 | 20 | 20 | 4 | 0 | 0,12(0,007-2,13)p=0,15 | non | yes | non | yes | Very low | Very low |
| Aouad | prospective randomised | Paediatric more 6 month (cardiac surgery) | Liban |  |  |  | 48 | 24 | 24 |  |  |  | non | non | non | yes | Very low | Very low |
| Sobolev* | meta-analysis | Cardiology | US | 248 | 4 |  | 4119 | 2295 | 1824 | 46 | 19 | 0,4(0,19-1,82) | non | non | yes | yes | low | low |
| Hilty | prospective randomised | ACR | US | 40 |  |  | 40 | 20 | 20 | 4 | 0 |  | non | non | non | yes | low | low |
| Summary of findings (ICU) |  |  |  |  |  |  | 4159 | 2315 | 1844 | 50 | 19 |  |  |  |  |  |  |  |
| Percentage |  |  |  |  |  |  |  |  |  | 0,02159827 | 0,01030369 |  |  |  |  |  |  |  |
| Arterial puncture ultrasound vs landmark |  |  |  |  |  |  |  |  |  |  |  |  |  |  |  |  |  |  |
| Brass | meta-analysis | miscelaneous | France | 210 | 4 | 4 | 311 |  |  |  |  | 0,4(0,14-1,16)p=0,09 |  |  |  |  | low |  |
| Hilty | prospective randomised | ACR | US | 40 |  |  | 40 | 20 | 20 | 4 | 0 | 0,12(0,007-2,13)p=0,15 |  |  |  |  |  |  |
| Yamagata | prospective randomised | Electrophysio study |  |  |  |  | 320 | 160 | 160 | 40 | 11 | 0,27(0,15-0,52)p=0,0001 |  |  |  |  |  |  |
| Aouad | prospective randomised | Paediatric more 6 month (cardiac surgery) | Liban |  |  |  | 48 | 24 | 24 | 1 | 3 | 3(0,33-27)p=0,32 |  |  |  |  |  |  |
| Sobolev* | meta-analysis | Cardiology | US | 248 | 4 |  | 382 | 170 | 212 | 25 | 11 | 0,26(0,05-1,34)p=0,11 |  |  |  |  |  |  |
| Haematoma ultrasound vs landmark |  |  |  |  |  |  |  |  |  |  |  |  |  |  |  |  |  |  |
| Other complications ultrasound vs landmark |  |  |  |  |  |  |  |  |  |  |  |  |  |  |  |  |  |  |
| Brass | meta-analysis | General | France | 210 | 4 | 4 | 311 |  |  |  |  | 0,49(0,11-2,12)p=0,34 |  |  |  |  | low |  |
| Summary of findings |  |  |  |  |  |  |  |  |  |  |  |  |  |  |  |  |  |  |
| percentage |  |  |  |  |  |  |  |  |  |  |  |  |  |  |  |  |  |  |
| Success ultrasound vs landmark |  |  |  |  |  |  |  |  |  |  |  |  |  |  |  |  |  |  |
| Brass | meta-analysis | general | France | 210 | 4 | 4 | 311 |  |  |  |  | 1,1(1-1,23)p=0,06 |  |  |  |  | moderated |  |
| Hilty | prospective randomised | ACR | US | 40 |  |  | 40 | 20 | 20 | 13 | 18 | 1,38(0,97-1,97)p=0,07 |  |  |  |  |  |  |
| Yamagata | prospective randomised | Electrophysio study |  |  |  |  | 320 | 160 | 160 | 138 | 159 | 22(3-161)p=0,0024 |  |  |  |  |  |  |
| Summary of findings |  |  |  |  |  |  |  |  |  |  |  |  |  |  |  |  |  |  |
| percentage |  |  |  |  |  |  |  |  |  |  |  |  |  |  |  |  |  |  |
| time to successful cannulation ultrasound vs landmark |  |  |  |  |  |  |  |  |  |  |  |  |  |  |  |  |  |  |
| Hilty | prospective randomised | ACR | US | 40 |  |  | 40 | 20 | 20 | 124 | 121 | NS |  |  |  |  |  |  |
| Yamagata | prospective randomised | Electrophysio study |  |  |  |  | 320 | 160 | 160 | 369 | 288 | p<0,05 |  |  |  |  |  |  |
| Aouad | prospective randomised | Paediatric more 6 month (cardiac surgery) | Liban |  |  |  | 48 | 24 | 24 | 380s | 155s | p=0,04 |  |  |  |  |  |  |
| Summary of findings |  |  |  |  |  |  |  |  |  |  |  |  |  |  |  |  |  |  |
| Success first attempt ultrasound vs landmark |  |  |  |  |  |  |  |  |  |  |  |  |  |  |  |  |  |  |
| Brass | meta-analysis | general | France | 210 | 4 | 3 | 224 |  |  |  |  | 1,73(1,34-2,22)p<0,0001 |  |  |  |  |  |  |
| Aouad | prospective randomised | Paediatric more 6 month (cardiac surgery) | Liban |  |  |  | 48 | 24 | 24 | 5 | 17 | 2,14(1,06-4,3)p=0,03 |  |  |  |  |  |  |
| Summary of findings |  |  |  |  |  |  |  |  |  |  |  |  |  |  |  |  |  |  |
| CVC blood stream infection ultrasound vs landmark |  |  |  |  |  |  |  |  |  |  |  |  |  |  |  |  |  |  |
| Summary of findings |  |  |  |  |  |  |  |  |  |  |  |  |  |  |  |  |  |  |
| *minor complications rr0,34(0,15-0,78) |  |  |  |  |  |  |  |  |  |  |  |  |  |  |  |  |  |  |

| **Ultrasound guidance catheter insertion subclavian/axillary vein** | | | | | | |  |  |  |  |  |  |  |  |  |  |  |  |
| --- | --- | --- | --- | --- | --- | --- | --- | --- | --- | --- | --- | --- | --- | --- | --- | --- | --- | --- |
| **study** | **Type of study** | **Population** | **country** | **Nb studies screened Meta-analysis)** | **Nb study used (Meta analysis)** | **nb studies** | **nb patients** | **Nb control group** | **Nb group ultrasons** | **Nb events control group** | **Nb events US group** | **RR** | **risk of Bias** | **inconsistencies** | **Indirect data** | **Imprecisions** | **Quality overall** | **Importance** |
| Complications ultrasound vs landmark | | | | |  |  |  |  |  |  |  |  |  |  |  |  |  |  |
| Brass | meta-analysis | not specified | France | 210 | 4 |  |  |  |  |  |  |  | non | non | yes | yes | low | low |
| Lalu | meta-analysis | not specified | Canada | 931 | 10 | 6 echography and 5 ICU neurosurgery | 638 | 324 | 310 | 97 | 33 | 0,298(0,439-0,202)p<0,0001 | non | non | yes | yes | low | low |
| Fragou | randomized prospective | ICU | Greece |  |  |  | 401 | 201 | 200 | 51 | 4 | 0,07(0,029-0,21)p<0,0001 | non | non | non | yes | moderated | moderated |
| Palepu | randomized prospective | ICU | India |  |  |  | 45 | 28 | 17 | 4 | 2 | 0,82(0,168-4,02)p=0,81 | non | non | non | yes | low | low |
| Gualtieri | randomized prospective | ICU | US |  |  |  | 33(55insertions) | 27 | 25 | 11 | 1 | 0,094(0,013-0,679)p=0,019 | non | non | non | yes | very low | very low |
| Kim (axillary) | randomized prospective | not well defined paediatrics | south Korea |  |  |  | 132 | 66 | 66 | 3 | 0 | 0,156(0,0082-2,97)p=0,21 | non | non | non | yes | low | low |
| Summary of findings (ICU) |  |  |  |  |  |  |  |  |  |  |  |  |  |  |  |  |  |  |
| Percentage |  |  |  |  |  |  |  |  |  |  |  |  |  |  |  |  |  |  |
| Arterial puncture ultrasound vs landmark |  |  |  |  |  |  |  |  |  |  |  |  |  |  |  |  |  |  |
| Brass | meta-analysis | not specified | France | 210 | 4 | 4 | 311 |  |  |  |  | 0,4(0,14-1,16)p=0,09 |  |  |  |  | low |  |
| Lalu | meta-analysis | not specified | Canada | 931 | 10 | 6 echography and 5 ICU neurosurgery | 638 | 324 | 310 | 18 | 4 | 0,278(0,651-0,119)p=0,003 |  |  |  |  |  |  |
| Fragou | randomized prospective | ICU | Greece |  |  |  | 401 | 201 | 200 | 11 | 1 | 0,09(0,011-0,7)p=0,021 |  |  |  |  |  |  |
| Kim (axillary) | randomized prospective | not well defined paediatrics (anaesthesia) | south Korea |  |  |  | 132 | 66 | 66 | 3 | 0 | 0,156(0,0082-2,97)p=0,21 |  |  |  |  |  |  |
| Gualtieri | randomized prospective | ICU |  |  |  |  | 33(55insertions) | 27 | 25 | 3 | 1 | 0,34(0,038-3,11)p=0,34 |  |  |  |  |  |  |
| Palepu | randomized prospective | ICU | India |  |  |  |  |  |  |  |  |  |  |  |  |  |  |  |
| Summary of findings |  |  |  |  |  |  |  |  |  |  |  |  |  |  |  |  |  |  |
| percentage |  |  |  |  |  |  |  |  |  |  |  |  |  |  |  |  |  |  |
| Haematoma ultrasound vs landmark |  |  |  |  |  |  |  |  |  |  |  |  |  |  |  |  |  |  |
| Lalu | meta-analysis | not specified | Canada | 931 | 10 | 6 echography and 5 ICU neurosurgery | 853 | 433 | 420 | 15 | 3 |  |  |  |  |  |  |  |
| Fragou | randomized prospective | ICU | Greece |  |  |  | 401 | 201 | 200 | 11 | 3 | 0,27(0,07-0,967)p=0,044 |  |  |  |  |  |  |
| Gualtieri | randomized prospective | ICU |  |  |  |  | 33(55insertions) | 27 | 25 | 5 | 0 | 0,094(0,005-1,62)p=0,10 |  |  |  |  |  |  |
| Other complications ultrasound vs landmark |  |  |  |  |  |  |  |  |  |  |  |  |  |  |  |  |  |  |
| Brass | meta-analysis | not specified | France | 210 | 4 | 4 | 311 |  |  |  |  | 0,49(0,11-2,12)p=0,34 |  |  |  |  | low |  |
| Lalu (pneumotorax) | meta-analysis | not specified | Canada | 931 | 10 | 6 echography and 5 ICU neurosurgery |  |  |  |  |  | 0,277(0,726-0,106)p=0,009 |  |  |  |  |  |  |
| Lalu (hemothorax) | meta-analysis | not specified | Canada | 931 | 10 | 6 echography and 5 ICU neurosurgery |  |  |  |  |  | 0,245(0,734-0,082)p=0,012 |  |  |  |  |  |  |
| Fragou | randomized prospective | ICU | Greece |  |  |  |  |  |  |  |  |  |  |  |  |  |  |  |
| Kim (axillary) | randomized prospective | paediatric poorly defined (anaesthetist) | Korea |  |  |  |  |  |  |  |  |  |  |  |  |  |  |  |
| Gualtieri | randomized prospective | ICU |  |  |  |  |  |  |  |  |  |  |  |  |  |  |  |  |
| Success ultrasound vs landmark |  |  |  |  |  |  |  |  |  |  |  |  |  |  |  |  |  |  |
| Brass | meta-analysis | not specified | France | 210 | 4 | 4 | 311 |  |  |  |  | 1,1(1-1,23)p=0,06 |  |  |  |  | moderated |  |
| Lalu | meta-analysis | not specified | Canada | 931 | 10 | 6 echography and 4 ICU |  |  |  |  |  |  |  |  |  |  |  |  |
| Hilty | randomized prospective | ACR | US | 40 |  |  |  |  |  |  |  |  |  |  |  |  |  |  |
| Fragou | randomized prospective | ICU | Greece |  |  |  |  |  |  |  |  |  |  |  |  |  |  |  |
| Kim (axillary) | randomized prospective | paediatric poorly defined (anaesthetist) | Korea |  |  |  |  |  |  |  |  |  |  |  |  |  |  |  |
| Gualtieri | randomized prospective | ICU |  |  |  |  |  |  |  |  |  |  |  |  |  |  |  |  |
| Palepu | randomized prospective | ICU | India |  |  |  |  |  |  |  |  |  |  |  |  |  |  |  |
| Summary of findings |  |  |  |  |  |  |  |  |  |  |  |  |  |  |  |  |  |  |
| percentage |  |  |  |  |  |  |  |  |  |  |  |  |  |  |  |  |  |  |
| time to successful cannulation ultrasound vs landmark |  |  |  |  |  |  |  |  |  |  |  |  |  |  |  |  |  |  |
|  |  |  |  |  |  |  |  |  |  |  |  |  |  |  |  |  |  |  |
| Fragou | randomized prospective | ICU | Greece |  |  |  | 401 | 201 | 200 | 45 | 27 | 0,6(0,39-0,93)p=0,0227 |  |  |  |  |  |  |
| Summary of findings |  |  |  |  |  |  |  |  |  |  |  |  |  |  |  |  |  |  |
| Success first attempt ultrasound vs landmark |  |  |  |  |  |  |  |  |  |  |  |  |  |  |  |  |  |  |
| Brass | meta-analysis | not specified | France | 210 | 4 | 3 | 224 |  |  |  |  | 1,73(1,34-2,22)p<0,0001 |  |  |  |  |  |  |
| Palepu | randomized prospective | ICU | India |  |  |  | 45 | 28 | 17 | 20 | 14 | 1,15(0,83-1,59)p=0,38 |  |  |  |  |  |  |
| Summary of findings |  |  |  |  |  |  |  |  |  |  |  |  |  |  |  |  |  |  |
| CVC blood stream infection ultrasound vs landmark |  |  |  |  |  |  |  |  |  |  |  |  |  |  |  |  |  |  |
| Summary of findings |  |  |  |  |  |  |  |  |  |  |  |  |  |  |  |  |  |  |

| **Ultrasound guidance catheter insertion central veins** | | |  |  |  |  |  |  |  |  |  |  |  |  |  |  |  |  |
| --- | --- | --- | --- | --- | --- | --- | --- | --- | --- | --- | --- | --- | --- | --- | --- | --- | --- | --- |
| **study** | **Type of study** | **Population** | **country** | **Nb studies screened Meta-analysis)** | **Nb study used (Meta-analysis)** | **nb studies** | **nb patients** | **Nb control group** | **Nb group ultrasons** | **nb events control group** | **Nb events US group** | **RR** | **risk of Bias** | **inconsistencies** | **Indirect data** | **Imprecisions** | **Quality overall** | **Importance** |
| Complications ultrasound vs landmark | | |  |  |  |  |  |  |  |  |  |  |  |  |  |  |  |  |
| Sazdov | prospective randomised | ICU | Macedonia |  |  |  | 400 | 200 | 200 | 43 | 10 | 0,255(0,13-0,48)p<0,0001 | no | no | no | yes | low | low |
| Lau | meta-analysis | Paediatric(4IJV,1 fem, 1 subclav)cardiac surgery ICU | US |  |  | 8 | 760 | 391 | 367 | 66 | 17 | 0,36(0,12-1,09)p=0,07 | no | no | ? | yes | moderate | moderate |
| Arterial puncture ultrasound vs landmark | | |  |  |  |  |  |  |  |  |  |  |  |  |  |  |  |  |
| Sazdov | prospective randomised | ICU | Macédoine |  |  |  | 400 | 200 | 200 | 16 | 2 | 0,125(0,29-0,536)p=0,0051 |  |  |  |  |  |  |
| Lau | meta-analysis | Paediatric(4IJV,1 fem, 1 subclav) | US |  |  | 8 | 760 | 391 | 367 | 66 | 17 | 0,36(0,12-1,09)p=0,07 |  |  |  |  |  |  |
| Haematoma ultrasound vs landmark | |  |  |  |  |  |  |  |  |  |  |  |  |  |  |  |  |  |
| Sazdov | prospective randomised | ICU | Macedonia |  |  |  | 400 | 200 | 200 | 20 | 8 | 0,4(0,18-0,88)p=0,02 |  |  |  |  |  |  |
| Other complications ultrasound vs landmark | | |  |  |  |  |  |  |  |  |  |  |  |  |  |  |  |  |
| Sazdov | prospective randomised | ICU | Macedonia |  |  |  | 400 | 200 | 200 | 7 | 0 | 0,06(0,0038-1,15)p=0,06 |  |  |  |  |  |  |
| Success ultrasound vs landmark | |  |  |  |  |  |  |  |  |  |  |  |  |  |  |  |  |  |
| Sazdov | prospective randomised | ICU | Macedonia |  |  |  | 400 | 200 | 200 | 181 | 196 | 1,08(1,03-1,13)p=0,0015 |  |  |  |  |  |  |
| Lau | meta-analysis | Paediatric(4IJV,1 fem, 1 subcla) | US |  |  | 8 | 760 | 391 | 367 | 268 | 338 | 1,32(1,1-1,58)p=0,003 |  |  |  |  |  |  |
| time to successful cannulation ultrasound vs landmark | | |  |  |  |  |  |  |  |  |  |  |  |  |  |  |  |  |
| Sazdov | prospective randomised | ICU | Macedonia |  |  |  | 400 | 200 | 200 | 20,1 mn | 13,6 mn | p<0,01 |  |  |  |  |  |  |
| Success first attempt ultrasound vs landmark | | |  |  |  |  |  |  |  |  |  |  |  |  |  |  |  |  |
| Sazdov | prospective randomised | ICU | Macedonia |  |  |  | 400 | 200 | 200 | 121 | 154 | 1,27(1,11-1,157)p=0,0005 |  |  |  |  |  |  |
| CVC blood stream infection ultrasound vs landmark | | |  |  |  |  |  |  |  |  |  |  |  |  |  |  |  |  |

| **Ultrasound guidance catheter insertion arteries** | | | | | | | | | | | | | | | | | | |
| --- | --- | --- | --- | --- | --- | --- | --- | --- | --- | --- | --- | --- | --- | --- | --- | --- | --- | --- |
| **study** | **Type of study** | **Population** | **country** | **Nb studies screened Meta-analysis)** | **Nb study used (Meta-analysis)** | **nb studies** | **nb patients** | **Nb control group** | **Nb group ultrasons** | **Nb events control group** | **Nb events US group** | **RR** | **risk of Bias** | **inconsistencies** | **Indirect data** | **Imprecisions** | **Quality overall** | **Importance** |
| Complications ultrasound vs landmark |  |  |  |  |  |  |  |  |  |  |  |  |  |  |  |  |  |  |
| Aouad-Maroun(cochrane 2016) | Meta-analysis(arterial) | paediatric |  | 296 | 5 | 2 | 222 |  |  |  |  | 0,2(0,07-0,6)p=0,004 | non | non | non | non | moderate | moderate |
| Gu (chest 2016) | Meta-analysis | all (no data in ICU), operative room most of time | China | 493 |  | 12 | 1992 |  |  |  |  |  | non | non | non | yes | moderate | moderate |
| Gu(crit care 2014) | Meta-analysis | all (no data in ICU), operative room most of time | China | 95 |  | 7 | 605 | 306 | 299 |  |  |  | non | non | non | yes | moderate | moderate |
| Gu(int care med 2014) | Meta-analysis | all (no data in ICU) | China |  |  | 5 | 370 | 219 | 210 |  |  |  | non | non | non | yes | moderate | moderate |
| Sobolev | Meta-analysis (femoral) |  | US | 1166 | 4 | 4 | 1422 | 703 | 719 | 34 | 16 | 0,51(0,28-0,9)p=0,02 | non | non | non | non | moderate | moderate |
| Gao | Meta-analysis(radial) | preoperative | China | 185 | 11 |  |  |  |  |  |  |  | non | non | non | yes | low | low |
| Li | Randomized controlled trial | ICU | China |  |  |  | 80 | 40 | 40 |  |  |  | non | non | non | yes | very low | very low |
| Arterial puncture ultrasound vs landmark |  |  |  |  |  |  |  |  |  |  |  |  |  |  |  |  |  |  |
|  |  |  |  |  |  |  |  |  |  |  |  |  |  |  |  |  |  |  |
| Haematoma ultrasound vs landmark |  |  |  |  |  |  |  |  |  |  |  |  |  |  |  |  |  |  |
| Sobolev | Meta-analysis (femoral) | all (no data in ICU) |  | 1166 | 4 | 4 | 1422 | 703 | 719 | 22 | 10 | 0,51(0,21-1,25)p=0,1) |  |  |  |  |  |  |
| Gu (chest 2016) | Meta-analysis | all (no data in ICU) | China |  |  |  |  |  |  |  |  | 0,39(0,16-0,95)p=0,04 |  |  |  |  |  |  |
| Gu(crit care 2014) | Meta-analysis | all (no data in ICU) | China |  |  |  |  |  |  |  |  | 0,17(0,07-0,41)p<0,0000 |  |  |  |  |  |  |
| Gu(int care med 2014) | Meta-analysis | all (no data in ICU) | China |  |  |  |  |  |  |  |  | 0,17(0,07-0,41)p<0,0001 |  |  |  |  |  |  |
| Li | prospective randomised | ICU | China |  |  |  | 80 | 40 | 40 | 8 | 1 | 0,125(0,016-0,95)p=0,045 |  |  |  |  |  |  |
| Anantasit | prospective randomised(radial) | paediatric | Thailand |  |  |  | 84 | 41 | 43 | 22 | 5 | 0,17(0,07-0,39)p<0,0001 |  |  |  |  |  |  |
| Other complications ultrasound vs landmark |  |  |  |  |  |  |  |  |  |  |  |  |  |  |  |  |  |  |
|  |  |  |  |  |  |  |  |  |  |  |  |  |  |  |  |  |  |  |
| Success ultrasound vs landmark |  |  |  |  |  |  |  |  |  |  |  |  |  |  |  |  |  |  |
| Aouad-Maroun(cochrane 2016) | Meta-analysis | general |  | 296 | 5 | 2 |  |  |  |  |  | 1,15(0,95-1,4)p=0,16 |  |  |  |  |  |  |
| Li | prospective randomised | ICU | China |  |  |  | 80 | 40 | 40 | 33 | 39 | 1,18(1,01-1,37)p=0,03 |  |  |  |  |  |  |
| Anantasit | prospective randomised(radial) | paediatric | Thailand |  |  |  | 84 | 41 | 43 | 16 | 26 | 4,18(1,57-11,4)p=0,004 |  |  |  |  |  |  |
| time to successful cannulation ultrasound vs landmark |  |  |  |  |  |  |  |  |  |  |  |  |  |  |  |  |  |  |
| Aouad-Maroun(cochrane 2016) | Meta-analysis | general |  | 296 | 5 | 4 |  |  |  | 151s | 65s |  |  |  |  |  |  |  |
| Sobolev | Meta-analysis (femoral) | General(any in ICU) |  | 1166 |  | 3 | 1322 | 656 | 666 | 373 | 327s | -25,6(-38;-13)p<0,0001 |  |  |  |  |  |  |
| Li | prospective randomised | ICU | China |  |  |  | 80 | 40 | 40 | 28 s | 9 s | p<0,01 |  |  |  |  |  |  |
| Anantasit | prospective randomised(radial) | paediatric | Thailand |  |  |  | 84 | 41 | 43 | 10,4 mn | 3,3 mn | 3,26(1,8-5,89)p<0,001 |  |  |  |  |  |  |
|  |  |  |  |  |  |  |  |  |  |  |  |  |  |  |  |  |  |  |
| Success first attempt ultrasound vs landmark |  |  |  |  |  |  |  |  |  |  |  |  |  |  |  |  |  |  |
| Aouad-Maroun(cochrane 2016) | Meta-analysis | general |  | 296 | 5 | 4 | 404 |  |  |  |  | 1,96(1,34-2,85)p=0,0005 |  |  |  |  |  |  |
| Gu (chest 2016) | Meta-analysis | General(aucune en ICU) | China |  |  |  | 2091 | 1049 | 1042 | 573 | 719 | 1,22(1,12-1,33)p<0,0001 |  |  |  |  |  |  |
| Gu(crit care 2014) | Meta-analysis | General(aucune en ICU) | China | 65 |  | 7 | 345 | 206 | 198 |  |  | 1,94(1,31-2,88)p=0,001 |  |  |  |  |  |  |
| Gu(int care med 2014) | Meta-analysis | General(aucune en ICU) | China |  |  | 5 | 370 | 219 | 210 |  |  | 1,84(1,47-2,32)p<0,0001 |  |  |  |  |  |  |
| Sobolev | Meta-analysis (femoral) | General(aucune en ICU) |  | 1166 |  | 3 | 1322 | 656 | 666 | 333 | 546 | 1,42(1,01-2)p=0,04 |  |  |  |  |  |  |
| Gao | Meta-analysis(radial) | anaesthesia | China | 185 | 11 |  | 902 | 455 | 447 | 201 | 306 | 1,47(1,22-1,76)p<0,0001 |  |  |  |  |  |  |
| Ganesh | prospective randomised | paediatric | US |  |  |  | 152 | 80 | 72 | 53 | 50 | 1,03(0,82-1,28)p=0,76 |  |  |  |  |  |  |
| Anantasit | prospective randomised(radial) | paediatric | Thailand |  |  |  | 84 | 41 | 43 | 12 | 26 | 2,03(1,13-3,64)p=0,018 |  |  |  |  |  |  |
| CVC blood stream infection ultrasound vs landmark |  |  |  |  |  |  |  |  |  |  |  |  |  |  |  |  |  |  |

| **Adults** |  |  |  |  |  |  |  |  |  |  |  |  |  |  |  |  |  |  |  |  |  |  |  |  |  |
| --- | --- | --- | --- | --- | --- | --- | --- | --- | --- | --- | --- | --- | --- | --- | --- | --- | --- | --- | --- | --- | --- | --- | --- | --- | --- |
| **Study** | **Journal** | **Country** | **Pub-year** | **Study type** | **Population** | **Intervention** | **Additional interventions** | **Implementation** | **Control** | **Outcome** | **Outcome (type)** | **Effect (IRR)** | **95%CI** | **Risk of bias** | **Indirectness** | **heterogeneity** | **imprecision** | **publicaton bias** | **Large magnitude of effect** | **Dose-response** | **Initial level** | **Up-/downgrade** | **GRADE** | **Exclude** | **Comments** |
| allen | Am J Infect Control 2014;42:643 | usa | 2014 | NCBA | ICU | Best practice (insertion) | insertion kit | simulator | 2.0/1000 cd | 0.8/1000 cd | CLABSI/1000 device-days | nr | nr | yes | no | na | na | na | na | no | 2 | -1 | 1 | exclude | Significant effect only in MICU, not in SICU |
| apisarnthanarak | Am J Infect Control 2010;38:449 | thailand | 2010 | NCBA | ICU | Best practice (insertion/care) | ab-chg | education sessions; feedback; posters | 17.0/1000 cd | 7.1/1000 cd | CABSI/1000 device-days | nr | nr | yes | no | na | na | na | na | no | 2 | -1 | 1 | exclude |  |
| barsuk | Arch Intern Med 2009;169:1420 | usa | 2009 | CBA | ICU | Best practice (insertion) |  | simulation; competency assessments | 5.3/1000 cd | 0.5/1000 cd | CRBSI/1000 device-days | 0,16 | 0.05-0.44 | uncertain | no | na | no | na | yes | no | 2 | 1 | 3 |  | Not the same individuals before and after |
| berenholtz | Infect Control Hosp Epidemiol 2014;35:56 | usa/puerto rico | 2014 | NCBA | ICU | Best practice (insertion) |  | cusp (national "on the cusp programme) | 2.0/1000cd | 1.2/1000 cd | CLABSI/1000 device-days | 0,59 | 0.53-0.64 | yes | no | yes | no | na | no | no | 2 | -1 | 1 |  |  |
| bion | BMJ Qual Saf 2013;22:110 | england | 2013 | CBA | ICU | Best practice (insertion/care) |  | focus groups; tc; cusp (matching-Michigan) | 3.7/1000 cd | 1.5/1000 cd | CVC-BSI/1000 device-days | 0,47 | 0.35-0.64 | yes | no | yes | no | na | uncertain | no | 2 | 0 | 2 |  | Non-randomly clustered to detect secular trend |
| burrell | MJA 2011;194:583 | australia | 2011 | NCBA | ICU | Best practice (insertion/care) |  | peer improvement team | 3.0/1000 cd | 1.2/1000 cd | CLABSI/1000 device-days | 0,5 | 0.4-0.8 | yes | no | uncertain | yes | na | yes | uncertain | 2 | -1 | 1 |  |  |
| de palo | Qual Saf Health Care 2010;19:555 | usa | 2010 | NCBA | ICU | Best practice (insertion) |  | cusp; Statewide programme (Rhode Island) | 3.7/1000 cd | 1.8/1000 cd | CLABSI/1000 device-days | nr | p=0.03 | yes | no | uncertain | uncertain | na | no | no | 2 | -1 | 1 |  |  |
| duane | Am Surg 2009;75:1166 | usa | 2009 | NCBA | ICU | Best practice (insertion) | line cart | (nurse) checklist | 16.5/1000 cd | 7.7/1000 cd | BSI/1000 device-days | 0,31 | 0.13-0.76 | uncertain | no | na | yes | na | yes | no | 2 | 0 | 2 |  |  |
| exline | Critical Care 2013;17:R41 | usa | 2013 | NCBA | ICU | Best practice (insertion) |  | multimodal; positive reinforcement | 2.7/1000 cd | 1.2/1000 cd | CLABSI/1000 device-days | 0,47 | 0.25-0.88 | uncertain | no | na | yes | na | no | no | 2 | 0 | 2 |  |  |
| galpern | Surgery 2008;144:492 | usa | 2008 | NCBA | ICU | Best practice (insertion) | line cart; ab-chg | multidisciplinary team | 5.0/1000 cd | 0.9/1000 cd | CLABSI/1000 device-days | nr | p<0.001 | uncertain | no | na | uncertain | na | uncertain | no | 2 | -1 | 1 |  |  |
| guerin | Am J Infect Control 2010;38:430 | usa | 2010 | NCBA | ICU | Best practice (care) |  | practical workshops | 5.7/1000 cd | 1.1/1000 cd | CLABSI/1000 device-days | 0,19 | 0.06-0.63 | uncertain | no | no | yes | na | yes | no | 2 | 0 | 2 |  |  |
| hansen | J Hosp Infect 2014;87:220 | germany | 2014 | ITS | ICU | Best practice (insertion/care) |  | Train-the-trainer; repetitive lectures | 2.3/1000 cd | 1.6/1000 cd | CLABSI/1000 device-days | 0,72 | 0.58-0.88 | no | no | uncertain | no | na | no | no | 2 | 0 | 2 |  | Voluntary participation of hospitals with baseline > German average |
| hocking | Intensive Crit Care Nurse 2013;29:137 | new zealand | 2013 | NCBA | ICU | Best practice (insertion/care) |  | checklist; reminders | 6.4/1000 cd | 1.5/1000 cd | CLABSI/1000 device-days | nr | p=0.02 | uncertain | no | no | uncertain | na | uncertain | no | 2 | -1 | 1 |  |  |
| hong | J Healthc Qual 2013;35:78 | usa | 2013 | NCBA | ICU | Best practice (insertion/care) |  | multi-disciplinary teams; pdca | 4.9/1000 cd | 2.2/1000 cd | CLABSI/1000 device-days | 0,53 | 0.45-0.61 | uncertain | no | uncertain | no | na | no | no | 2 | 0 | 2 |  |  |
| jaggi | Int J Infect Dis 2013;17:e1218 | india | 2013 | NCBA | ICU | Best practice (insertion/care) | ab-chg | outcome and performance feedback | 6.4/1000 cd | 3.9/1000 cd | CLABSI/1000 device-days | 0,47 | 0.31-0.70 | yes | no | yes | yes | na | yes | no | 2 | -1 | 1 |  |  |
| khalid | Am J Infect Control 2013;41:1209 | saudi arabia | 2013 | NCBA | ICU | Best practice (insertion/care) | ag-chg-coated CVCs; chg body wash | education sessions; audits of bundle compliance | 6.9/1000 cd | 1.1/1000 cd | CLABSI/1000 device-days | 0,15 | 0.05-0.44 | yes | yes | no | yes | na | yes | no | 2 | -1 | 1 | exclude | indirect evidence due to other interventions (chg body wash, impregnated catheters) |
| khouli | Chest 2011;139:80 | usa | 2011 | RCT | ICU | Best practice (insertion): simulator vs. conventional training of interns | | | 3.4/1000 cd | 1.0/1000 cd | CRBSI/1000 device-days | 0,3 | 0.10-0.91 | no | yes | no | yes | na | yes | no | 4 | -1 | 3 |  | indirect evidence because the intervention was not on the quality itself but the implementation |
| Kim | Am J Infect Control 2011;39:640 | usa | 2011 | NCBA | ICU | Best practice (insertion/care) |  | checklists; feedback | 9.0/1000 cd | 2.7/1000 cd | CRBSI/1000 device-days | nr | p<0.001 | uncertain | no | no | uncertain | na | uncertain | no | 2 | -1 | 1 |  |  |
| klintworth | Am J Infect Control 2014;42:685 | australia | 2014 | NCBA | ICU | Best practice (insertion/care) | ab-chg; chg body wash | education and feedback | 2.3/1000 cd | 0.9/1000 cd | CLABSI/1000 device-days | 0,39 | 0.20-0.72 | uncertain | yes | no | yes | na | yes | no | 2 | -1 | 1 | exclude | Sub-analysis data of a hospital wide programme; indirect evidence du to chg body wash |
| latif | Infect Control Hosp Epidemiol 2015;36:816 | usa | 2015 | NCBA | ICU/PICU/NICU | Best practice (insertion/care) |  | cusp; webinars; external change agents | 2.6/1000 cd | 1.8/1000 cd | CLABSI/1000 device-days | 0,62 | 0.46-0.83 | yes | uncertain | yes | no | na | no | no | 2 | -1 | 1 |  |  |
| leblebicioglu | Ann Clin Microbiol Antimicrob 2013;12:10 | turkey | 2013 | NCBA | ICU | Best practice (insertion/care) | ab-chg | outcome and performance feedback | 22.7/1000 cd | 12.0/1000 cd | CLABSI/1000 device-days | 0,61 | 0.43-0.87 | yes | no | uncertain | yes | na | no | no | 2 | -1 | 1 |  |  |
| marra | Am J Infect Control 2010;38:434 | brazil | 2010 | NCBA | ICU | Best practice (insertion) | ab-chg | leadership engagement; random audits | 6.4/1000 cd | 3.2/1000 cd | CLABSI/1000 device-days | nr | p<0.001 | uncertain | no | no | uncertain | na | uncertain | no | 2 | -1 | 1 |  |  |
| marsteller | Crit Care Med 2012;40:2933 | usa | 2012 | RCT | ICU | Best practice (insertion) | ab-chg | checklist; cusp | 2.2/1000 cd | 1.3/1000 cd | CLABSI/1000 device-days | 0,19 | 0.06-0.57 | no | no | no | yes | na | yes | no | 4 | 0 | 4 |  |  |
| mc laws | Crit Care Med 2012;40:388 | australia | 2012 | NCBA | ICU | Best practice (insertion) | ab-chg | peer improvement team | 3.8/1000 cd | 1.6/1000 cd | CLABSI/1000 device-days | 0,43 | 0.25-0.74 | uncertain | no | uncertain | yes | na | yes | uncertain | 2 | 0 | 2 |  |  |
| mueller | AM J Med Qual 2014;29:191 | usa | 2014 | NCBA | ICU | Best practice (insertion/care) |  | no info | 1.2/1000 cd | 0.8/1000 cd | CLABSI/1000 device-days | 0,69 | 0.51-0.93 | uncertain | no | no | yes | na | no | no | 2 | -1 | 1 |  |  |
| palomar | Crit Care Med 2013;41:2364 | spain | 2013 | NCBA | ICU | Best practice (insertion/care) | ab-chg | cusp (matching-Michigan) | 3.1/1000 cd | 1.1/1000 cd | CLABSI/1000 device-days | 0,5 | 0.39-0.63 | uncertain | no | uncertain | no | na | yes | no | 2 | 0 | 2 |  |  |
| peredo | Eur J Clin Microbiol Infect Dis 2010;29:1173 | spain | 2010 | NCBA | ICU | Best practice (insertion) |  | educational sessions; checklist | 6.7/1000 cd | 2.4/1000 cd | CRBSI/1000 device-days | 0,36 | 0.16-0.80 | uncertain | no | no | yes | na | yes | no | 2 | -1 | 1 |  |  |
| perez-parra | Infect Control Hosp Epidemiol 2010;31:964 | spain | 2010 | NCBA | ICU | Best practice (insertion/care) |  | single education; knowledge test | 4.2/1000 cd | 2.9/1000 cd | CLABSI/1000 device-days | 0,69 | 0.44-1.08 | no | no | no | yes | na | no | no | 2 | -1 | 1 |  |  |
| pronovost | N Engl J Med 2006;355:2725 | usa | 2006 | NCBA | ICU | Best practice (insertion) | ab-chg | cusp | 7.7/1000 cd | 1.4/1000 cd | CRBSI/1000 device-days | 0,34 | 0.23-0.50 | uncertain | no | uncertain | no | na | uncertain | no | 2 | -1 | 1 |  | IRR only reported for quarters compared to baseline |
| render | BMJ Qual Saf 2011;20:725 | usa | 2011 | NCBA | ICU | Best practice (insertion) | ab-chg | "inpatient evaluation center" | 3.8/1000 cd | 1.8/1000 cd | CLABSI/1000 device-days | 0,47 | 0.40-0.55 | uncertain | no | uncertain | no | na | yes | yes | 2 | 0 | 2 |  |  |
| rosenthal | Infect Control Hosp Epidemiol 2010;31:1264 | inicc | 2010 | NCBA | ICU | Best practice (insertion/care) | ab-chg | performance audits | 14.4/1000 cd | 9.7/1000 cd | CLABSI/1000 device-days | 0,67 | 0.58-0.77 | uncertain | no | uncertain | no | na | no | no | 2 | -1 | 1 |  |  |
| sacks | Am J Surg 2014;207:817 | usa | 2014 | CBA | ICU | Best practice (insertion) | ab-chg | focus groups; pdca; checklists | 5.0/1000 cd | 1.6/1000 cd | CLABSI/1000 device-days | 0,32 | 0.08-0.99 | no | no | no | yes | na | yes | no | 2 | 0 | 2 |  | control group; however, the difference not statistically tested |
| santana | Infect Control Hosp Epidemiol 2008;29:1171 | brazil | 2008 | NCBA | ICU | Best practice (insertion) | ab-chg | audits; lectures | 9.5/1000 cd | 5.4/1000 cd | CLABSI/1000 device-days | 0,46 | 0.21-1.02 | uncertain | no | no | yes | na | yes | no | 2 | -1 | 1 |  |  |
| tang | BMC Infectious Diseases 2014;14:356 | taiwan | 2014 | NCBA | ICU | Best practice (insertion/care) | ab-chg | lectures; videos | 1.7/1000 cd | 0.6/1000 cd | CLABSI/1000 device-days | nr | p=0.04 | uncertain | no | no | uncertain | na | uncertain | no | 2 | -1 | 1 |  | retrospective baseline |
| thom | Am J Infect Control 2014;42:129 | usa | 2014 | NCBA | ICU | Best practice (insertion/care) |  | unit-based quality nurse | 5.0/1000 cd | 1.5/1000 cd | CLABSI/1000 device-days | nr | p=0.005 | yes | no | no | uncertain | na | uncertain | no | 2 | -1 | 1 |  | the intervention was the presence of a new quality-nurse in the ICU; also other ICUs reduced CLABSI |
| van der kooi | Intensive Care Med 2018;44:48 | europe | 2018 | RCT | ICU | Best practice (insertion) | ab-chg | centralised workshops | 2.4/1000 cd | 0.9/1000 cd | CRBSI/1000 device-days | 0,39 | 0.32-0.48 | no | no | yes | no | na | yes | yes | 4 | 0 | 4 |  |  |
| venkatram | J Crit Care 2010;25:174e11 | usa | 2010 | NCBA | ICU | Best practice (insertion) | ab-chg | collaborative; leadership engagement; workshops | 10.8/1000 cd | 1.7/1000 cd | CRBSI/1000 device-days | 0,16 | 0.13-0.18 | uncertain | no | no | no | na | yes | no | 2 | 0 | 2 |  |  |
| warren | Infect Control Hosp Epidemiol 2006;27:662 | usa | 2006 | NCBA | ICU | Best practice (insertion) |  | lectures; self-study | 11.2/1000 cd | 8.9/1000 cd | CABSI/1000 device-days | 0,79 | 0.76-0.93 | uncertain | no | uncertain | no | na | no | no | 2 | -1 | 1 |  |  |
| zingg | Crit Care Med 2009;37:2167 | switzerland | 2009 | NCBA | ICU | Best practice (care) |  | focus groups; bedside teaching | 3.9/1000 cd | 1.0/1000 cd | CRBSI/1000 device-days | 0,25 | 0.11-0.58 | no | no | no | yes | na | yes | no | 2 | 0 | 2 |  |  |
|  |  |  |  |  |  |  |  |  |  |  |  |  |  |  |  |  |  |  |  |  |  |  |  |  |  |
| Summary |  |  |  |  |  |  |  |  |  |  |  |  |  |  |  |  |  |  |  |  |  |  |  |  |  |
| **Studies (N)** | **Journal** | **Country** | **Pub-year** | **Study type** | **Population** | **Intervention** | **Additional interventions** | **Implementation** | **Control** | **Outcome** | **Outcome (type)** | **Effect (IRR)** | **95%CI** | **Risk of bias** | **Indirectness** | **heterogeneity** | **imprecision** | **publication bias** | **Large magnitude of effect** | **Dose-response** | **Initial level** | **Up-/downgrade** | **GRADE** | **Exclude** | **Comments** |
| 33 | na | na | na | mixed | adult ICUs | mix of best practice interventions (bundles) | |  | no intervention (usually baseline in an NCBA) | | CLABSI/CRBSI/1000 device-days | 0.25-0.67 |  | uncertain | no | yes | yes | yes | yes | no | 2 | -1 | 1 |  |  |
|  |  |  |  |  |  |  |  |  |  |  |  |  |  |  |  |  |  |  |  |  |  |  |  |  |  |
| **legends: ICU intensive care unit; RCT: randomized controlled trial; Controlled Best pratice assessment; not controlled Best practice assessment; ab antibiotic ; chg: chlorhexidine;** | | | |  |  |  |  |  |  |  |  |  |  |  |  |  |  |  |  |  |  |  |  |  |  |

| **Children** | |  |  |  |  |  |  |  |  |  |  |  |  |  |  |  |  |  |  |  |  |  |  |  |  |
| --- | --- | --- | --- | --- | --- | --- | --- | --- | --- | --- | --- | --- | --- | --- | --- | --- | --- | --- | --- | --- | --- | --- | --- | --- | --- |
| **Study** | **Journal** | **Country** | **Pub-year** | **Study type** | **Population** | **Intervention** | **Additional interventions** | **Implementation** | **Control** | **Outcome** | **Outcome (type)** | **Effect (IRR)** | **95%CI** | **Risk of bias** | **Indirectness** | **heterogeneity** | **imprecision** | **publication bias** | **Large magnitude of effect** | **Dose-response** | **Initial level** | **Up-/downgrade** | **GRADE** | **Exclude** | **Comments** |
| abramczyk | Braz J Infect Dis 2011;15:573 | brazil | 2011 | NCBA | PICU | Best practice (insertion/care) | none | lectures (slides) | 23.1/1000 cd | 13.9/1000 cd | BSI/1000 device-days | 0,61 | 0.32-1.14 | yes | no | uncertain | yes | na | no | no | 2 | -1 | 1 |  |  |
| ahmed | Pediatr Crit Care Med 2012;13:e69 | usa | 2012 | NCBA | PICU | Best practice (insertion/care) | dedicated insertion cart; checklist | multidisciplinary team; vascular access team; bedside teaching | 7.9/1000 cd | 1.3/1000 cd | CABSI/1000 device-days | 0,56 | 0.36-0.87 | yes | no | uncertain | yes | na | uncertain | no | 2 | -1 | 1 |  |  |
| bion | BMJ Qual Saf 2013;22:110 | england | 2013 | CBA | PICU | Best practice (insertion/care) | | focus groups; tc | 5.7/1000 cd | 2.9/1000 cd | CVC-BSI/1000 device-days | nr | p=0.625 | yes | no | yes | uncertain | na | uncertain | no | 2 | -1 | 1 |  |  |
| costello | Pediatrics 2008;121:915 | usa | 2008 | ITS | PICU | Best practice (insertion/care) | ab-chg | feedback; IPC-staffing; goal sheets | 7.8-4.7/1000 cd | 2.3/1000 cd | CLABSI/1000 device-days | 0,54 | 0.31-0.94 | yes | no | uncertain | yes | na | no | yes | 2 | -1 | 1 |  | relocation between baseline and intervention |
| esteban | Pediatr Crit Care Med 2013;14:525 | spain | 2013 | NCBA | PICU | Best practice (insertion) | creation of IPC-team | education of hh; workshops | 8.1/1000 cd | 6.0/1000 cd | CABSI/1000 device-days | 0,57 | 0.24-1.37 | no | no | uncertain | yes | na | no | no | 2 | -1 | 1 | exclude |  |
| jeong | Am J Infect Control 2013;41:710 | south korea | 2013 | NCBA | children in adult ICUs | Best practice (insertion) | | checklist | 3.7/1000 cd | 0/1000 cd | CLABSI/1000 device-days | 0 | 0.00-0.84 | yes | yes | no | yes | na | no | no | 2 | -1 | 1 |  | Sub-analysis for children; no significant reduction among adults |
| latif | Infect Control Hosp Epidemiol 2015;36:816 | uae | 2015 | NCBA | ICU/PICU/NICU | Best practice (insertion/care) | | cusp; webinars; external change agents | 2.6/1000 cd | 1.8/1000 cd | CLABSI/1000 device-days | 0,62 | 0.46-0.83 | yes | uncertain | yes | no | na | no | no | 2 | -1 | 1 |  |  |
| miller | Pediatrics 2010;125:206 | usa | 2010 | ITS | PICU | Best practice (insertion/care) | ab-chg | workshops; champions | 5.4/1000 cd | 3.1/1000 cd | CABSI/1000 device-days | 0,57 | 0.45-0.74 | yes | no | uncertain | no | na | no | yes | 2 | -1 | 1 |  | compared to historical cohort |
| pageler | Pediatrics 2014;133:e738 | usa | 2014 | NCBA | PICU | checklist for best practice | | checklist | 2.6/1000 cd | 0.7/1000 cd | CLABSI/1000 device-days | nr | p=0.03 | yes | no | no | uncertain | na | uncertain | no | 2 | -1 | 1 |  | compared to historical cohort |
| rosenthal | Infection 2012;40:415 | inicc | 2012 | NCBA | PICU | Best practice (insertion/care) | ab-chg | education; performance feedback | 10.7/1000 cd | 5.2/1000 cd | CLABSI/1000 device-days | 0,48 | 0.29-0.94 | yes | no | yes | yes | na | yes | no | 2 | -1 | 1 |  | very long period; poorly defined time points |
|  |  |  |  |  |  |  |  |  |  |  |  |  |  |  |  |  |  |  |  |  |  |  |  |  |  |
| Summary |  |  |  |  |  |  |  |  |  |  |  |  |  |  |  |  |  |  |  |  |  |  |  |  |  |
| **Studies (N)** | **Journal** | **Country** | **Pub-year** | **Study type** | **Population** | **Intervention** | **Additional interventions** | **Implementation** | **Control** | **Outcome** | **Outcome (type)** | **Effect (IRR)** | **95%CI** | **Risk of bias** | **Indirectness** | **heterogeneity** | **imprecision** | **publication bias** | **Large magnitude of effect** | **Dose-response** | **Initial level** | **Up-/downgrade** | **GRADE** | **Exclude** | **Comments** |
| 9 | na | na | na | mixed | PICUs | mix of best practice interventions (bundles) | | |  | no intervention (usually baseline in an NCBA) | CLABSI/CRBSI/1000 device-days | 0.48-0.62 |  | yes | no | yes | yes | yes | no | no | 2 | -1 | 1 |  |  |

| **Adults** |  |  |  |  |  |  |  |  |  |  |  |  |  |  |  |  |  |  |  |  |  |  |  |  |  |
| --- | --- | --- | --- | --- | --- | --- | --- | --- | --- | --- | --- | --- | --- | --- | --- | --- | --- | --- | --- | --- | --- | --- | --- | --- | --- |
| **Study** | **Journal** | **Country** | **Pub-year** | **Study type** | **Population** | **Intervention** | **Additional interventions** | **Implementation** | **Control** | **Outcome** | **Outcome (type)** | **Effect (IRR)** | **95%CI** | **Risk of bias** | **Indirectness** | **heterogeneity** | **imprecision** | **publicaton bias** | **Large magnitude of effect** | **Dose-response** | **Initial level** | **Up-/downgrade** | **GRADE** | **Exclude** | **Comments** |
| allen | Am J Infect Control 2014;42:643 | usa | 2014 | NCBA | ICU | Best practice (insertion) | insertion kit | simulator | 2.0/1000 cd | 0.8/1000 cd | CLABSI/1000 device-days | nr | nr | yes | no | na | na | na | na | no | 2 | -1 | 1 | exclude | Significant effect only in MICU, not in SICU |
| apisarnthanarak | Am J Infect Control 2010;38:449 | thailand | 2010 | NCBA | ICU | Best practice (insertion/care) | ab-chg | education sessions; feedback; posters | 17.0/1000 cd | 7.1/1000 cd | CABSI/1000 device-days | nr | nr | yes | no | na | na | na | na | no | 2 | -1 | 1 | exclude |  |
| barsuk | Arch Intern Med 2009;169:1420 | usa | 2009 | CBA | ICU | Best practice (insertion) |  | simulation; competency assessments | 5.3/1000 cd | 0.5/1000 cd | CRBSI/1000 device-days | 0,16 | 0.05-0.44 | uncertain | no | na | no | na | yes | no | 2 | 1 | 3 |  | Not the same individuals before and after |
| berenholtz | Infect Control Hosp Epidemiol 2014;35:56 | usa/puerto rico | 2014 | NCBA | ICU | Best practice (insertion) |  | cusp (national "on the cusp programme) | 2.0/1000cd | 1.2/1000 cd | CLABSI/1000 device-days | 0,59 | 0.53-0.64 | yes | no | yes | no | na | no | no | 2 | -1 | 1 |  |  |
| bion | BMJ Qual Saf 2013;22:110 | england | 2013 | CBA | ICU | Best practice (insertion/care) |  | focus groups; tc; cusp (matching-Michigan) | 3.7/1000 cd | 1.5/1000 cd | CVC-BSI/1000 device-days | 0,47 | 0.35-0.64 | yes | no | yes | no | na | uncertain | no | 2 | 0 | 2 |  | Non-randomly clustered to detect secular trend |
| burrell | MJA 2011;194:583 | australia | 2011 | NCBA | ICU | Best practice (insertion/care) |  | peer improvement team | 3.0/1000 cd | 1.2/1000 cd | CLABSI/1000 device-days | 0,5 | 0.4-0.8 | yes | no | uncertain | yes | na | yes | uncertain | 2 | -1 | 1 |  |  |
| de palo | Qual Saf Health Care 2010;19:555 | usa | 2010 | NCBA | ICU | Best practice (insertion) |  | cusp; State wide programme (Rhode Island) | 3.7/1000 cd | 1.8/1000 cd | CLABSI/1000 device-days | nr | p=0.03 | yes | no | uncertain | uncertain | na | no | no | 2 | -1 | 1 |  |  |
| duane | Am Surg 2009;75:1166 | usa | 2009 | NCBA | ICU | Best practice (insertion) | line cart | (nurse) checklist | 16.5/1000 cd | 7.7/1000 cd | BSI/1000 device-days | 0,31 | 0.13-0.76 | uncertain | no | na | yes | na | yes | no | 2 | 0 | 2 |  |  |
| exline | Critical Care 2013;17:R41 | usa | 2013 | NCBA | ICU | Best practice (insertion) |  | multimodal; positive reinforcement | 2.7/1000 cd | 1.2/1000 cd | CLABSI/1000 device-days | 0,47 | 0.25-0.88 | uncertain | no | na | yes | na | no | no | 2 | 0 | 2 |  |  |
| galpern | Surgery 2008;144:492 | usa | 2008 | NCBA | ICU | Best practice (insertion) | line cart; ab-chg | multidisciplinary team | 5.0/1000 cd | 0.9/1000 cd | CLABSI/1000 device-days | nr | p<0.001 | uncertain | no | na | uncertain | na | uncertain | no | 2 | -1 | 1 |  |  |
| guerin | Am J Infect Control 2010;38:430 | usa | 2010 | NCBA | ICU | Best practice (care) |  | practical workshops | 5.7/1000 cd | 1.1/1000 cd | CLABSI/1000 device-days | 0,19 | 0.06-0.63 | uncertain | no | no | yes | na | yes | no | 2 | 0 | 2 |  |  |
| hansen | J Hosp Infect 2014;87:220 | germany | 2014 | ITS | ICU | Best practice (insertion/care) |  | Train-the-trainer; repetitive lectures | 2.3/1000 cd | 1.6/1000 cd | CLABSI/1000 device-days | 0,72 | 0.58-0.88 | no | no | uncertain | no | na | no | no | 2 | 0 | 2 |  | Voluntary participation of hospitals with baseline > German average |
| hocking | Intensive Crit Care Nurse 2013;29:137 | new zealand | 2013 | NCBA | ICU | Best practice (insertion/care) |  | checklist; reminders | 6.4/1000 cd | 1.5/1000 cd | CLABSI/1000 device-days | nr | p=0.02 | uncertain | no | no | uncertain | na | uncertain | no | 2 | -1 | 1 |  |  |
| hong | J Healthc Qual 2013;35:78 | usa | 2013 | NCBA | ICU | Best practice (insertion/care) |  | multi-disciplinary teams; pdca | 4.9/1000 cd | 2.2/1000 cd | CLABSI/1000 device-days | 0,53 | 0.45-0.61 | uncertain | no | uncertain | no | na | no | no | 2 | 0 | 2 |  |  |
| jaggi | Int J Infect Dis 2013;17:e1218 | india | 2013 | NCBA | ICU | Best practice (insertion/care) | ab-chg | outcome and performance feedback | 6.4/1000 cd | 3.9/1000 cd | CLABSI/1000 device-days | 0,47 | 0.31-0.70 | yes | no | yes | yes | na | yes | no | 2 | -1 | 1 |  |  |
| khalid | Am J Infect Control 2013;41:1209 | saudi arabia | 2013 | NCBA | ICU | Best practice (insertion/care) | ag-chg-coated CVCs; chg body wash | education sessions; audits of bundle compliance | 6.9/1000 cd | 1.1/1000 cd | CLABSI/1000 device-days | 0,15 | 0.05-0.44 | yes | yes | no | yes | na | yes | no | 2 | -1 | 1 | exclude | indirect evidence due to other interventions (chg body wash, impregnated catheters) |
| khouli | Chest 2011;139:80 | usa | 2011 | RCT | ICU | Best practice (insertion): simulator vs. conventional training of interns | | | 3.4/1000 cd | 1.0/1000 cd | CRBSI/1000 device-days | 0,3 | 0.10-0.91 | no | yes | no | yes | na | yes | no | 4 | -1 | 3 |  | indirect evidence because the intervention was not on the quality itself but the implementation |
| Kim | Am J Infect Control 2011;39:640 | usa | 2011 | NCBA | ICU | Best practice (insertion/care) |  | checklists; feedback | 9.0/1000 cd | 2.7/1000 cd | CRBSI/1000 device-days | nr | p<0.001 | uncertain | no | no | uncertain | na | uncertain | no | 2 | -1 | 1 |  |  |
| klintworth | Am J Infect Control 2014;42:685 | australia | 2014 | NCBA | ICU | Best practice (insertion/care) | ab-chg; chg body wash | education and feedback | 2.3/1000 cd | 0.9/1000 cd | CLABSI/1000 device-days | 0,39 | 0.20-0.72 | uncertain | yes | no | yes | na | yes | no | 2 | -1 | 1 | exclude | Sub analysis data of a hospital wide programme; indirect evidence du to chg body wash |
| latif | Infect Control Hosp Epidemiol 2015;36:816 | uae | 2015 | NCBA | ICU/PICU/NICU | Best practice (insertion/care) |  | cusp; webinars; external change agents | 2.6/1000 cd | 1.8/1000 cd | CLABSI/1000 device-days | 0,62 | 0.46-0.83 | yes | uncertain | yes | no | na | no | no | 2 | -1 | 1 |  |  |
| leblebicioglu | Ann Clin Microbiol Antimicrob 2013;12:10 | turkey | 2013 | NCBA | ICU | Best practice (insertion/care) | ab-chg | outcome and performance feedback | 22.7/1000 cd | 12.0/1000 cd | CLABSI/1000 device-days | 0,61 | 0.43-0.87 | yes | no | uncertain | yes | na | no | no | 2 | -1 | 1 |  |  |
| marra | Am J Infect Control 2010;38:434 | brazil | 2010 | NCBA | ICU | Best practice (insertion) | ab-chg | leadership engagement; random audits | 6.4/1000 cd | 3.2/1000 cd | CLABSI/1000 device-days | nr | p<0.001 | uncertain | no | no | uncertain | na | uncertain | no | 2 | -1 | 1 |  |  |
| marsteller | Crit Care Med 2012;40:2933 | usa | 2012 | RCT | ICU | Best practice (insertion) | ab-chg | checklist; cusp | 2.2/1000 cd | 1.3/1000 cd | CLABSI/1000 device-days | 0,19 | 0.06-0.57 | no | no | no | yes | na | yes | no | 4 | 0 | 4 |  |  |
| mc laws | Crit Care Med 2012;40:388 | australia | 2012 | NCBA | ICU | Best practice (insertion) | ab-chg | peer improvement team | 3.8/1000 cd | 1.6/1000 cd | CLABSI/1000 device-days | 0,43 | 0.25-0.74 | uncertain | no | uncertain | yes | na | yes | uncertain | 2 | 0 | 2 |  |  |
| mueller | AM J Med Qual 2014;29:191 | usa | 2014 | NCBA | ICU | Best practice (insertion/care) |  | no info | 1.2/1000 cd | 0.8/1000 cd | CLABSI/1000 device-days | 0,69 | 0.51-0.93 | uncertain | no | no | yes | na | no | no | 2 | -1 | 1 |  |  |
| palomar | Crit Care Med 2013;41:2364 | spain | 2013 | NCBA | ICU | Best practice (insertion/care) | ab-chg | cusp (matching-Michigan) | 3.1/1000 cd | 1.1/1000 cd | CLABSI/1000 device-days | 0,5 | 0.39-0.63 | uncertain | no | uncertain | no | na | yes | no | 2 | 0 | 2 |  |  |
| peredo | Eur J Clin Microbiol Infect Dis 2010;29:1173 | spain | 2010 | NCBA | ICU | Best practice (insertion) |  | educational sessions; checklist | 6.7/1000 cd | 2.4/1000 cd | CRBSI/1000 device-days | 0,36 | 0.16-0.80 | uncertain | no | no | yes | na | yes | no | 2 | -1 | 1 |  |  |
| perez-parra | Infect Control Hosp Epidemiol 2010;31:964 | spain | 2010 | NCBA | ICU | Best practice (insertion/care) |  | single education; knowledge test | 4.2/1000 cd | 2.9/1000 cd | CLABSI/1000 device-days | 0,69 | 0.44-1.08 | no | no | no | yes | na | no | no | 2 | -1 | 1 |  |  |
| pronovost | N Engl J Med 2006;355:2725 | usa | 2006 | NCBA | ICU | Best practice (insertion) | ab-chg | cusp | 7.7/1000 cd | 1.4/1000 cd | CRBSI/1000 device-days | 0,34 | 0.23-0.50 | uncertain | no | uncertain | no | na | uncertain | no | 2 | -1 | 1 |  | IRR only reported for quarters compared to baseline |
| render | BMJ Qual Saf 2011;20:725 | usa | 2011 | NCBA | ICU | Best practice (insertion) | ab-chg | "inpatient evaluation center" | 3.8/1000 cd | 1.8/1000 cd | CLABSI/1000 device-days | 0,47 | 0.40-0.55 | uncertain | no | uncertain | no | na | yes | yes | 2 | 0 | 2 |  |  |
| rosenthal | Infect Control Hosp Epidemiol 2010;31:1264 | inicc | 2010 | NCBA | ICU | Best practice (insertion/care) | ab-chg | performance audits | 14.4/1000 cd | 9.7/1000 cd | CLABSI/1000 device-days | 0,67 | 0.58-0.77 | uncertain | no | uncertain | no | na | no | no | 2 | -1 | 1 |  |  |
| sacks | Am J Surg 2014;207:817 | usa | 2014 | CBA | ICU | Best practice (insertion) | ab-chg | focus groups; pdca; checklists | 5.0/1000 cd | 1.6/1000 cd | CLABSI/1000 device-days | 0,32 | 0.08-0.99 | no | no | no | yes | na | yes | no | 2 | 0 | 2 |  | control group; however, the difference not statistically tested |
| santana | Infect Control Hosp Epidemiol 2008;29:1171 | brazil | 2008 | NCBA | ICU | Best practice (insertion) | ab-chg | audits; lectures | 9.5/1000 cd | 5.4/1000 cd | CLABSI/1000 device-days | 0,46 | 0.21-1.02 | uncertain | no | no | yes | na | yes | no | 2 | -1 | 1 |  |  |
| tang | BMC Infectious Diseases 2014;14:356 | taiwan | 2014 | NCBA | ICU | Best practice (insertion/care) | ab-chg | lectures; videos | 1.7/1000 cd | 0.6/1000 cd | CLABSI/1000 device-days | nr | p=0.04 | uncertain | no | no | uncertain | na | uncertain | no | 2 | -1 | 1 |  | retrospective baseline |
| thom | Am J Infect Control 2014;42:129 | usa | 2014 | NCBA | ICU | Best practice (insertion/care) |  | unit-based quality nurse | 5.0/1000 cd | 1.5/1000 cd | CLABSI/1000 device-days | nr | p=0.005 | yes | no | no | uncertain | na | uncertain | no | 2 | -1 | 1 |  | the intervention was the presence of a new quality-nurse in the ICU; also other ICUs reduced CLABSI |
| van der kooi | Intensive Care Med 2018;44:48 | europe | 2018 | RCT | ICU | Best practice (insertion) | ab-chg | centralised workshops | 2.4/1000 cd | 0.9/1000 cd | CRBSI/1000 device-days | 0,39 | 0.32-0.48 | no | no | yes | no | na | yes | yes | 4 | 0 | 4 |  |  |
| venkatram | J Crit Care 2010;25:174e11 | usa | 2010 | NCBA | ICU | Best practice (insertion) | ab-chg | collaborative; leadership engagement; workshops | 10.8/1000 cd | 1.7/1000 cd | CRBSI/1000 device-days | 0,16 | 0.13-0.18 | uncertain | no | no | no | na | yes | no | 2 | 0 | 2 |  |  |
| warren | Infect Control Hosp Epidemiol 2006;27:662 | usa | 2006 | NCBA | ICU | Best practice (insertion) |  | lectures; self-study | 11.2/1000 cd | 8.9/1000 cd | CABSI/1000 device-days | 0,79 | 0.76-0.93 | uncertain | no | uncertain | no | na | no | no | 2 | -1 | 1 |  |  |
| zingg | Crit Care Med 2009;37:2167 | switzerland | 2009 | NCBA | ICU | Best practice (care) |  | focus groups; bedside teaching | 3.9/1000 cd | 1.0/1000 cd | CRBSI/1000 device-days | 0,25 | 0.11-0.58 | no | no | no | yes | na | yes | no | 2 | 0 | 2 |  |  |
|  |  |  |  |  |  |  |  |  |  |  |  |  |  |  |  |  |  |  |  |  |  |  |  |  |  |
| Summary |  |  |  |  |  |  |  |  |  |  |  |  |  |  |  |  |  |  |  |  |  |  |  |  |  |
| **Studies (N)** | **Journal** | **Country** | **Pub-year** | **Study type** | **Population** | **Intervention** | **Additional interventions** | **Implementation** | **Control** | **Outcome** | **Outcome (type)** | **Effect (IRR)** | **95%CI** | **Risk of bias** | **Indirectness** | **heterogeneity** | **imprecision** | **publication bias** | **Large magnitude of effect** | **Dose-response** | **Initial level** | **Up-/downgrade** | **GRADE** | **Exclude** | **Comments** |
| 33 | na | na | na | mixed | adult ICUs | mix of best practice interventions (bundles) | |  | no intervention (usually baseline in an NCBA) | | CLABSI/CRBSI/1000 device-days | 0.25-0.67 |  | uncertain | no | yes | yes | yes | yes | no | 2 | -1 | 1 |  |  |

| **Children** | |  |  |  |  |  |  |  |  |  |  |  |  |  |  |  |  |  |  |  |  |  |  |  |  |
| --- | --- | --- | --- | --- | --- | --- | --- | --- | --- | --- | --- | --- | --- | --- | --- | --- | --- | --- | --- | --- | --- | --- | --- | --- | --- |
| **Study** | **Journal** | **Country** | **Pub-year** | **Study type** | **Population** | **Intervention** | **Additional interventions** | **Implementation** | **Control** | **Outcome** | **Outcome (type)** | **Effect (IRR)** | **95%CI** | **Risk of bias** | **Indirectness** | **heterogeneity** | **imprecision** | **publication bias** | **Large magnitude of effect** | **Dose-response** | **Initial level** | **Up-/downgrade** | **GRADE** | **Exclude** | **Comments** |
| abramczyk | Braz J Infect Dis 2011;15:573 | brazil | 2011 | NCBA | PICU | Best practice (insertion/care) | none | lectures (slides) | 23.1/1000 cd | 13.9/1000 cd | BSI/1000 device-days | 0,61 | 0.32-1.14 | yes | no | uncertain | yes | na | no | no | 2 | -1 | 1 |  |  |
| ahmed | Paediatric Crit Care Med 2012;13:e69 | usa | 2012 | NCBA | PICU | Best practice (insertion/care) | dedicated insertion cart; checklist | multidisciplinary team; vascular access team; bedside teaching | 7.9/1000 cd | 1.3/1000 cd | CABSI/1000 device-days | 0,56 | 0.36-0.87 | yes | no | uncertain | yes | na | uncertain | no | 2 | -1 | 1 |  |  |
| bion | BMJ Qual Saf 2013;22:110 | england | 2013 | CBA | PICU | Best practice (insertion/care) | | focus groups; tc | 5.7/1000 cd | 2.9/1000 cd | CVC-BSI/1000 device-days | nr | p=0.625 | yes | no | yes | uncertain | na | uncertain | no | 2 | -1 | 1 |  |  |
| costello | Paediatrics 2008;121:915 | usa | 2008 | ITS | PICU | Best practice (insertion/care) | ab-chg | feedback; IPC-staffing; goal sheets | 7.8-4.7/1000 cd | 2.3/1000 cd | CLABSI/1000 device-days | 0,54 | 0.31-0.94 | yes | no | uncertain | yes | na | no | yes | 2 | -1 | 1 |  | relocation between baseline and intervention |
| esteban | Pediatr Crit Care Med 2013;14:525 | spain | 2013 | NCBA | PICU | Best practice (insertion) | creation of IPC-team | education of hh; workshops | 8.1/1000 cd | 6.0/1000 cd | CABSI/1000 device-days | 0,57 | 0.24-1.37 | no | no | uncertain | yes | na | no | no | 2 | -1 | 1 | exclude |  |
| jeong | Am J Infect Control 2013;41:710 | south korea | 2013 | NCBA | children in adult ICUs | Best practice (insertion) | | checklist | 3.7/1000 cd | 0/1000 cd | CLABSI/1000 device-days | 0 | 0.00-0.84 | yes | yes | no | yes | na | no | no | 2 | -1 | 1 |  | Sub analysis for children; no significant reduction among adults |
| latif | Infect Control Hosp Epidemiol 2015;36:816 | uae | 2015 | NCBA | ICU/PICU/NICU | Best practice (insertion/care) | | cusp; webinars; external change agents | 2.6/1000 cd | 1.8/1000 cd | CLABSI/1000 device-days | 0,62 | 0.46-0.83 | yes | uncertain | yes | no | na | no | no | 2 | -1 | 1 |  |  |
| miller | Paediatrics 2010;125:206 | usa | 2010 | ITS | PICU | Best practice (insertion/care) | ab-chg | workshops; champions | 5.4/1000 cd | 3.1/1000 cd | CABSI/1000 device-days | 0,57 | 0.45-0.74 | yes | no | uncertain | no | na | no | yes | 2 | -1 | 1 |  | compared to historical cohort |
| pageler | Paediatrics 2014;133:e738 | usa | 2014 | NCBA | PICU | checklist for best practice | | checklist | 2.6/1000 cd | 0.7/1000 cd | CLABSI/1000 device-days | nr | p=0.03 | yes | no | no | uncertain | na | uncertain | no | 2 | -1 | 1 |  | compared to historical cohort |
| rosenthal | Infection 2012;40:415 | inicc | 2012 | NCBA | PICU | Best practice (insertion/care) | ab-chg | education; performance feedback | 10.7/1000 cd | 5.2/1000 cd | CLABSI/1000 device-days | 0,48 | 0.29-0.94 | yes | no | yes | yes | na | yes | no | 2 | -1 | 1 |  | very long period; poorly defined time points |
|  |  |  |  |  |  |  |  |  |  |  |  |  |  |  |  |  |  |  |  |  |  |  |  |  |  |
| Summary |  |  |  |  |  |  |  |  |  |  |  |  |  |  |  |  |  |  |  |  |  |  |  |  |  |
| **Studies (N)** | **Journal** | **Country** | **Pub-year** | **Study type** | **Population** | **Intervention** | **Additional interventions** | **Implementation** | **Control** | **Outcome** | **Outcome (type)** | **Effect (IRR)** | **95%CI** | **Risk of bias** | **Indirectness** | **heterogeneity** | **imprecision** | **publication bias** | **Large magnitude of effect** | **Dose-response** | **Initial level** | **Up-/downgrade** | **GRADE** | **Exclude** | **Comments** |
| 9 | na | na | na | mixed | PICUs | mix of best practice interventions (bundles) | | |  | no intervention (usually baseline in an NCBA) | CLABSI/CRBSI/1000 device-days | 0.48-0.62 |  | yes | no | yes | yes | yes | no | no | 2 | -1 | 1 |  |  |

*Guidewire exchange might be proposed, instead of new-site catheter insertion, in selected patients with catheter dysfunction or with suspected infection.*

Most RCTs and systematic reviews failed to demonstrate benefit of new-site catheter insertion over guidewire exchange in patients with proven or suspected infection and in patients with catheter dysfunction ^1-7^. These studies were however of intermediate or high risk of bias, had limited statistical power; and were performed in heterogeneous populations^1-8^. Although some studies reported conflicting results but were observational cohort failing to adjust for clustering effect^9^. When compared to guidewire exchange, new site insertion was associated with no benefits in term of new catheter-related blood stream infection, colonization rate, time to colonization or catheter exit-site infection^1,2,5,7^. Guidewire exchange was associated with lower rate of mechanical complication^2,4^, but also, when it comes to dialysis catheter, to a higher rate of dysfunction^1^. Guidewire exchange was advocated for management of catheter infection in patients with tunneled long-term catheter such cancer patients^4,10^ and patients with ESKD^5,8,11^.

Although additional adequately powered RCTs are needed, guidewire exchange may be proposed instead of new-site catheter insertion in patients with high hemorrhage risk^4,10^, to preserve vascular access^5,8,11^, or to decrease workload without delaying removal of a catheter suspect to be source of an infection^8^.

**REFERENCES**

1. Coupez, E. *et al.* Guidewire exchange vs new site placement for temporary dialysis catheter insertion in ICU patients: is there a greater risk of colonization or dysfunction? *Crit. Care Lond. Engl.* **20,** 230 (2016).

2. Cook, D. *et al.* Central venous catheter replacement strategies: a systematic review of the literature. *Crit. Care Med.* **25,** 1417–1424 (1997).

3. Chaftari, A.-M. *et al.* The use of minocycline-rifampin coated central venous catheters for exchange of catheters in the setting of staphylococcus aureus central line associated bloodstream infections. *BMC Infect. Dis.* **14,** 518 (2014).

4. Chaftari, A.-M. *et al.* Novel approach using antimicrobial catheters to improve the management of central line-associated bloodstream infections in cancer patients. *Cancer* **117,** 2551–2558 (2011).

5. Saleh, H. M., Tawfik, M. M. & Abouellail, H. Prospective, randomized study of long-term hemodialysis catheter removal versus guidewire exchange to treat catheter-related bloodstream infection. *J. Vasc. Surg.* **66,** 1427–1431.e1 (2017).

6. Chua, H.-R. *et al.* Initial and extended use of femoral versus nonfemoral double-lumen vascular catheters and catheter-related infection during continuous renal replacement therapy. *Am. J. Kidney Dis. Off. J. Natl. Kidney Found.* **64,** 909–917 (2014).

7. Parbat, N. *et al.* The microbiological and clinical outcome of guide wire exchanged versus newly inserted antimicrobial surface treated central venous catheters. *Crit. Care Lond. Engl.* **17,** R184 (2013).

8. Aslam, S., Vaida, F., Ritter, M. & Mehta, R. L. Systematic review and meta-analysis on management of hemodialysis catheter-related bacteremia. *J. Am. Soc. Nephrol. JASN* **25,** 2927–2941 (2014).

9. Garnacho-Montero, J. *et al.* Risk factors and prognosis of catheter-related bloodstream infection in critically ill patients: a multicenter study. *Intensive Care Med.* **34,** 2185–2193 (2008).

10. Martínez, E. *et al.* Central venous catheter exchange by guidewire for treatment of catheter-related bacteraemia in patients undergoing BMT or intensive chemotherapy. *Bone Marrow Transplant.* **23,** 41–44 (1999).

11. O’Horo, J. C., Silva, G. L. M. & Safdar, N. Anti-infective locks for treatment of central line-associated bloodstream infection: a systematic review and meta-analysis. *Am. J. Nephrol.* **34,** 415–422 (2011).

**Tableau 1.** Summary of evidences – Tunnelled or non-tunnelled central catheter

| **Certainty assessment** | | | | | | | | | | | | **№ of patients** | | **Effect** | | **Certainty** | **Importance** |
| --- | --- | --- | --- | --- | --- | --- | --- | --- | --- | --- | --- | --- | --- | --- | --- | --- | --- |
| **№ of studies** | **Study design** | | **Risk of bias** | | **Inconsistency** | | **Indirectness** | | **Imprecision** | | **Other considerations** | **Guidewire exchange** | **New site insertion** | **Relative (95% CI)** | **Absolute (95% CI)** |  |  |
| Risk of catheter colonisation - Cook D et al. CCM 1997 (follow up: median 7 days; assessed with: Culture) | | | | | | | | | | | | | | | | | |
| 12 | randomised trials | | serious ^a^ | | serious ^b^ | | not serious | | not serious | | all plausible residual confounding would reduce the demonstrated effect | 52/888 (5.9%) |  | **RR 1.26** (0.87 to 1.84) | **1 fewer per 1 000** (from 1 fewer to 2 fewer) | ⨁⨁⨁◯ MODERATE | CRITICAL |
| Risk of catheter colonisation or infection - Cook et al. CCM 1997 (follow up: median 7 days; assessed with: Culture) | | | | | | | | | | | | | | | | | |
| 9 | randomised trials | | serious ^a,b^ | | serious ^b^ | | not serious | | not serious | | all plausible residual confounding would reduce the demonstrated effect |  |  | **RR 1.72** (0.89 to 3.33) | **2 fewer per 1 000** (from 1 fewer to 3 fewer) | ⨁⨁⨁◯ MODERATE | CRITICAL |
| Feasibility - Hemato or BMT patients - Martinez et al.BMT 1999 (follow up: range 4 days to 135 days) | | | | | | | | | | | | | | | | | |
| 1 | observational studies | | serious ^c^ | | not serious | | not serious | | not serious | | all plausible residual confounding would reduce the demonstrated effect |  | - | - | - | ⨁⨁◯◯ LOW | NOT IMPORTANT |
| Antibiotic lock vs. Guidewire exchange - O'Horo et al. Am J Nephrol 2011 | | | | | | | | | | | | | | | | | |
| 2 | observational studies | | serious ^a,b^ | | not serious | | not serious | | serious | | all plausible residual confounding would reduce the demonstrated effect | 111 | 116 | - | mean **63 days higher** (54 higher to 73 higher) | ⨁◯◯◯ VERY LOW | IMPORTANT |
| Coated catheter guidewire Exchange vs. retained or removed - Chaftari et al. BMC Infect dis 2014 (follow up: 3 months) | | | | | | | | | | | | | | | | | |
| 1 | observational studies | | serious ^d^ | | not serious | | not serious | | not serious | | all plausible residual confounding would suggest spurious effect, while no effect was observed | 0/8 (0.0%) | 12/32 (37.5%) | not estimable |  | ⨁⨁◯◯ LOW | NOT IMPORTANT |
| Coated catheter Guidewire exchange vs. removal in patients with infection - Chaftari et al. Cancer 2011 (timing of exposure: mean 7 days; assessed with: Composite score) | | | | | | | | | | | | | | | | | |
| 1 | observational studies | | serious ^e^ | | not serious | | not serious | | not serious | | all plausible residual confounding would reduce the demonstrated effect | 38 cases 2 controls | | **RR 1.24** (1.08 to 1.43) | - | ⨁⨁◯◯ LOW | IMPORTANT |
|  |  |  |  |  |  |  |  |  |  |  |  | - | 76.0% |  | **182 more per 1 000** (from 61 more to 327 more) |  |  |
| Risk of Catheter related bacterial infection with dedicated focus for Guidewire exchange- Garnacho-Montero et al. Intensive Care Med 2008 (Culture: mean 7 days; assessed with: Composite score) | | | | | | | | | | | | | | | | | |
| observational studies | | serious ^e^ | | not serious | | not serious | | not serious | | all plausible residual confounding would reduce the demonstrated effect | | 61 cases 1537 controls  Risk reported as risk of CR-BSI with Guidewire exchange | | **RR 4.15** (2.02 to 8.5) | - | ⨁⨁◯◯ LOW | IMPORTANT |

**Tableau 2.** Summary of evidences – Tunnelled or non-tunnelled dialysis catheter

| **Certainty assessment** | | | | | | | **№ of patients** | | **Effect** | | **Certainty** | **Importance** |
| --- | --- | --- | --- | --- | --- | --- | --- | --- | --- | --- | --- | --- |
| **№ of studies** | **Study design** | **Risk of bias** | **Inconsistency** | **Indirectness** | **Imprecision** | **Other considerations** | **Catheter guidewire exchange** | **newly inserted catheter** | **Relative (95% CI)** | **Absolute (95% CI)** |  |  |
| Cure rate when compared to systemic ATBx in catheter infection -Cuffed tunneled catheter- Aslam et al. JASN 2014 (follow up: median 45 days; assessed with: clinical resolution with no recurrent bac- teremia within $3 weeks of follow-up from treatment initiation) | | | | | | | | | | | | |
| 28 | observational studies | very serious ^a^ | very serious ^b^ | serious ^c^ | not serious | strong association all plausible residual confounding would reduce the demonstrated effect | 237/353 (67.1%) | 317/697 (45.5%) | **OR 2.88** (1.82 to 4.55) | **251 more per 1 000** (from 148 more to 337 more) | ⨁◯◯◯ VERY LOW | IMPORTANT |
| Cure rate when compared to Catheter removal - Long term dialysis catheter - Saleh et al. J Vasc Surg 2017 (follow up: 45 days; assessed with: Lack of infection at day 45) | | | | | | | | | | | | |
| 1 | randomised trials | very serious ^d^ | very serious ^e^ | very serious ^b,c^ | not serious | none |  |  | **OR 0.88** (0.43 to 1.79) | **1 fewer per 1 000** (from 0 fewer to 2 fewer) | ⨁◯◯◯ VERY LOW | NOT IMPORTANT |
| Rate of colonisation or infection (guidewire exchange vs. puncture) - Chua et al. Am J Kidney Dis 2014 (follow up: median 7 days; assessed with: CRCOL et CRBSI) | | | | | | | | | | | | |
| 1 | observational studies | serious ^a^ | not serious | not serious | not serious | all plausible residual confounding would reduce the demonstrated effect | 5/53 (9.4%) | 3/53 (5.7%) | **OR 1.21** (0.51 to 2.90) | **11 more per 1 000** (from 27 fewer to 92 more) | ⨁⨁◯◯ LOW | IMPORTANT |
| Rate of colonisation or infection (Guidewire exchange vs. Puncture) - Coupez et al. Crit Care 2016 (follow up: median 5 days; assessed with: Culture) | | | | | | | | | | | | |
| 1 | observational studies | serious ^a^ | serious ^f^ | not serious | not serious | all plausible residual confounding would reduce the demonstrated effect | 10/178 (5.6%) | 3.0% | **HR 4.11** (0.14 to 122.30) | **88 more per 1 000** (from 26 fewer to 946 more) | ⨁◯◯◯ VERY LOW | IMPORTANT |
|  |  |  |  |  |  |  |  | 5.6% |  | **155 more per 1 000** (from 48 fewer to 943 more) |  |  |
|  |  |  |  |  |  |  |  | 10.0% |  | **251 more per 1 000** (from 85 fewer to 900 more) |  |  |
| Rate of dysfunction (Guidewire vs. puncture)- Coupez et al. Crit Care 2016 (follow up: median 5 days; assessed with: by investigator on predefined criteria) | | | | | | | | | | | | |
| 1 | observational studies | serious ^a^ | not serious | not serious | not serious | strong association dose response gradient | 67/178 (37.6%) | 10.7% | **HR 3.67** (2.07 to 6.49) | **233 more per 1 000** (from 102 more to 413 more) | ⨁⨁⨁◯ MODERATE | CRITICAL |
|  |  |  |  |  |  |  |  | 15.7% |  | **309 more per 1 000** (from 141 more to 513 more) |  |  |
|  |  |  |  |  |  |  |  | 20.7% |  | **366 more per 1 000** (from 174 more to 571 more) |  |  |
| Rate of colonization (Guidewire exchange vs. new puncture) - Parbat et al. Critical Care 2013 (follow up: median 5 days; assessed with: Culture) | | | | | | | | | | | | |
| 1 | observational studies | serious ^g^ | serious ^f^ | serious ^f^ | not serious | all plausible residual confounding would reduce the demonstrated effect | 5/81 (6.2%) | 3.3% | **RR 0.98** (0.31 to 3.10) | **1 fewer per 1 000** (from 23 fewer to 69 more) | ⨁◯◯◯ VERY LOW | IMPORTANT |
|  |  |  |  |  |  |  |  | 6.3% |  | **1 fewer per 1 000** (from 43 fewer to 132 more) |  |  |
|  |  |  |  |  |  |  |  | 9.3% |  | **2 fewer per 1 000** (from 64 fewer to 195 more) |  |  |
| Rate of colonization (Guidewire exchange vs. new puncture) - Parbat et al. Critical Care 2013 (follow up: median 5 days; assessed with: CRBSI) | | | | | | | | | | | | |
| 1 | observational studies | serious ^g^ | not serious | not serious | not serious | none | 2/145 (1.4%) | 0.8% | **RR 0.73** (0.13 to 4.41) | **2 fewer per 1 000** (from 7 fewer to 27 more) | ⨁◯◯◯ VERY LOW | IMPORTANT |
|  |  |  |  |  |  |  |  | 1.8% |  | **5 fewer per 1 000** (from 16 fewer to 61 more) |  |  |
|  |  |  |  |  |  |  |  | 2.8% |  | **8 fewer per 1 000** (from 24 fewer to 95 more) |  |  |

**CI:** Confidence interval; **OR:** Odds ratio; **HR:** Hazard Ratio; **RR:** Risk ratio

#### Explanations

a. Incomplete adjustment for confounders, including clustering effect

b. Not ICU patients

c. Cuffed catheter

d. Not following equator and lot of missing information (including randomization process)

e. Lack of full results including cure rate per arm

f. Colonization rather than infection

g. Incomplete adjustment and matching limited to day of catheter change
